# Rapid and Efficient Access to Novel Bio-Inspired 3-Dimensional Tricyclic SpiroLactams as Privileged Structures via Meyers’ Lactamization

**DOI:** 10.3390/ph16030413

**Published:** 2023-03-08

**Authors:** Salia Tangara, Léo Faïon, Catherine Piveteau, Frédéric Capet, Romain Godelier, Marion Michel, Marion Flipo, Benoit Deprez, Nicolas Willand, Baptiste Villemagne

**Affiliations:** 1 Univ. Lille, Inserm, Institut Pasteur de Lille, U1177—Drugs and Molecules for Living Systems, F-59000 Lille, France; 2 Univ. Lille, CNRS, Centrale Lille, ENSCL, Univ. Artois, UMR 8181—UCCS—Unité de Catalyse et Chimie du Solide, F-59000 Lille, France

**Keywords:** focused chemical library, natural-like products, tricyclic spirolactams, Fsp^3^-rich compounds, privileged scaffolds

## Abstract

The concept of privileged structure has been used as a fruitful approach for the discovery of novel biologically active molecules. A privileged structure is defined as a semi-rigid scaffold able to display substituents in multiple spatial directions and capable of providing potent and selective ligands for different biological targets through the modification of those substituents. On average, these backbones tend to exhibit improved drug-like properties and therefore represent attractive starting points for hit-to-lead optimization programs. This article promotes the rapid, reliable, and efficient synthesis of novel, highly 3-dimensional, and easily functionalized bio-inspired tricyclic spirolactams, as well as an analysis of their drug-like properties.

## 1. Introduction

Privileged structures are described in the literature as molecular patterns used to provide useful ligands targeting more than one receptor [1]. They are often defined as a semi-rigid scaffold displaying substituents in multiple directions, whose derivatization can lead to potent and selective ligands for various biological targets. Privileged structures usually exhibit good drug-like properties, making them very attractive candidates to enrich libraries of drug-like compounds for screening [2].

For decades, considerable efforts have been made to identify the structural and physico-chemical properties that make small molecules more likely to become drugs. These characteristics would then be helpful to improve the drug discovery success rate through the design and synthesis of focused drug-like molecule libraries. More recently, Lovering et al. reported that the clinical success of many drugs and drug candidates is correlated to their molecular complexity [3]. They proposed two descriptors to measure molecular complexity. The first one is the carbon bond saturation represented by Fsp^3^, defined as the ratio between the number of sp^3^-hybridized carbon atoms and the total carbon count. The second one is the total number of chiral carbons in the molecule. The authors demonstrated that molecules displaying a high Fsp^3^ and numerous stereocenters tend to exhibit good drug-like properties such as high solubility and low promiscuity and have a lower rate of attrition during each stage of the drug discovery process [4]. Moreover, these compounds present an enhanced three-dimensional structure due to their saturated carbon atoms (C-sp^3^), which allows access to different chemical spaces compared to flat, sp^2^-rich compounds. In addition, several research groups have succeeded in improving the drug properties of lead compounds by increasing their Fsp^3^ value [5]. Despite these efforts, commercial chemical libraries for drug discovery screening programs are still dominated by sp^2^-rich compounds due to the lack of synthetic routes for stereoselective construction of complex sp^3^-rich molecules.

Drugs issued from natural products typically possess a higher Fsp^3^ value (>0.5) and a greater number of stereogenic centers compared to drugs from completely synthetic origins [6]. In addition, the core scaffolds of natural products are often considered ‘’privileged structures” due to their presence in many bioactive molecules [7] and constitute an important source of inspiration in drug design and development [8]. Several studies have revealed that the acquisition of a diversity of scaffolds inspired by natural products can be a powerful tool to explore new chemical spaces and identify lead compounds in the search for new drug candidates [9,10,11,12]. However, libraries of natural products are still scarce due to their highly complex structures and synthetic pathways that usually require multiple steps with very low yields [13].

**Figure 1:**
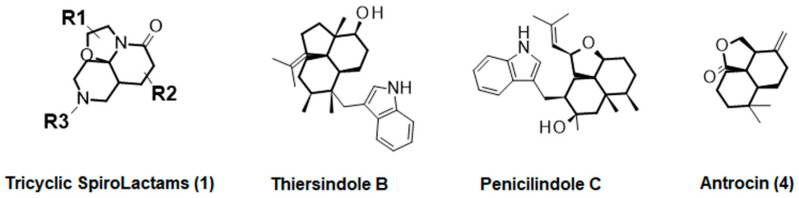
Structure of natural products Thiersindole B, Penicilindole C, Antrocin, and target Tricyclic SpiroLactams containing fused tricyclic spiro-ring systems.

As an alternative, easily accessible synthetic molecules exhibiting the structural features of natural products and privileged structures are highly desirable. To address these issues, we report here the design of and facile access to a focused library of novel Fsp^3^-rich tricyclic spirolactams (**1**) (Figure 1). This family displays structural complexity similar to that found in natural products [14], particularly in indole diterpenoids (Thiersindole B [15] and Penicilindole C [16]), and a sesquiterpene γ-lactone Antrocin. Thiersindole B (**2**) and Penicilindole C (**3**) were isolated from *Penicillium thiersii* and *Eupenicillium* sp. HJ002, respectively. Antrocin (**4**) was isolated from *Antrodia camphorate* [17] and can be synthesized in 11 steps from commercially available 3-methyl-2-cyclohexenone [18]. Antrocin has shown significant activity against the proliferation of metastatic breast cancer cell lines (MDA-MB-231) [19] and has potential as a therapeutic agent for the prevention of Alzheimer’s disease [20].

In addition to possessing the structural hallmarks of natural products, these novel tricyclic spirolactams (**1**) display a rigid scaffold with three functionalization points (R1, R2, and R3) (Figure 1), spread out in three distinct vectors, allowing the synthesis of a large number of analogues to probe new chemical spaces and explore additional relevant biological space areas [21,22]. These novel bio-inspired sp^3^-rich tricyclic spirolactams (**1**) can therefore be considered “privileged structures”, and libraries of such molecules have the potential to provide high-quality hit compounds with significant interest in drug-discovery screening programs.

## 2. Results and Discussion

**Scheme 1:**
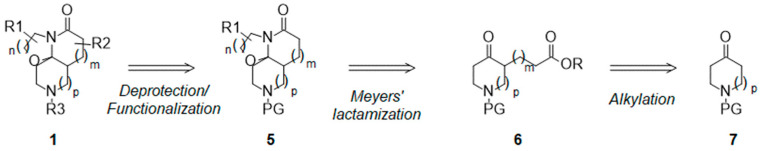
Retrosynthetic analysis of Tricyclic SpiroLactams **1**.

Over the past decade, our team has been interested in the development of rapid and efficient synthetic routes to access unprecedented 3-dimensional privileged structures and fragments for drug discovery applications [23,24,25,26]. As a continuation of our efforts to enrich our screening libraries with novel privileged scaffolds, we designed here an original and concise synthetic approach that allows rapid and efficient access to new tricyclic spirolactams from commercially available *N*-protected cyclic ketones. The three-dimensional core of these molecules (**5**) can be obtained through Meyers’ lactamization between amino-alcohols and the keto-ester or acid (**6**), which is derived from *N*-protected cyclic ketone (**7**) through alkylation (Scheme 1).

Meyers’ lactamization reaction has been widely used for the stereoselective synthesis of bicyclic lactams, which may be utilized as chiral building blocks for the stereoselective construction of five- and six-membered nitrogen-containing heterocycles [27,28]. It is a well-known tool for the synthesis of natural products (especially alkaloids) from phenylglycinol-derived bi- and tricyclic lactams [29,30,31]. However, to the best of our knowledge, this methodology has not been extended to derive 4-piperidone δ–keto-ester until the work of our group [32]. Herein, we aim to extend Meyers’ lactamization reaction to piperidones, pyrrolidinones, azapanones, aminocyclohexanone-derived keto-acids and keto-esters, and original amino-alcohols in order to rapidly generate a collection of novel tricyclic spirolactams with a diversity of scaffolds that have similar complexity to natural products.

### 2.1. Optimization of Meyers’ Lactamization Reaction

We have previously described the synthesis of bicyclic and tricyclic lactams from keto-acids by this methodology using water or a water/methanol mix as solvent [32]. However, the reported yield for the reaction between 3-(1-benzyl-4-oxo-3-piperidyl) propanoic acid chlorhydrate (**8**) and phenylglycinol remained moderate (65%). Meyers’ lactamization is usually performed in toluene under reflux with or without a Dean-Stark apparatus [33,34,35]. Using toluene as the solvent, a conversion of 66% was obtained (Table 1, entry 1). Next, the reaction was investigated under microwave irradiations (µW) [36] in order to reduce the reaction time and to allow for heating at higher temperatures (150 °C) (Table 1, entry 2). Under these conditions, the conversion rate was improved to 76%. We hypothesized that the low solubility of keto-acid **8** in toluene could be responsible for the incomplete conversion and that using a less polar (and therefore more soluble in toluene) analog such as keto-ester **9** could improve conversion. Levacher and collaborators have reported that Meyers’ lactamization was more efficient with a di-aryl keto-ester than with its keto-acid analog. In addition, they described the synthesis of various lactams by treatment of diverse di-aryl keto-esters with phenylglycinol using pivalic acid (*t*-BuCO_2_H) catalysis under microwave irradiations [37]. Interestingly, complete conversion was obtained when these conditions were applied to δ–keto-ester **9** (Table 1, entry 3). In addition, the reaction was completely stereoselective, and only the expected lactam **10** was formed during the reaction.

### 2.2. Scope of Meyers’ Lactamization Reaction

#### 2.2.1. Synthesis of Lactams with Octahydrooxazolo [2,3-j][1,6]naphthyridin-5-ones Core ([**5**.**6**.**6**] Ring System) from Benzyl-Keto-Ester

To explore the scope of these optimized reaction conditions (Table 1, entry 3) to generate lactams with octahydrooxazolo [2,3-j][1,6]naphthyridin-5-ones core ([**5**.**6**.**6**] ring system), benzyl δ-keto-ester **9** was selected as the model substrate to react with several chiral β–amino-alcohols. Complete conversion of starting material **9** was observed when reacted with L-alaninol, L-valinol, and L-*tert*-leucinol (Table 2, entries 1–3). The stereoselectivity of the reaction was found to be correlated to the steric hindrance of the substituent in position 2 of the amino alcohol. The expected products **11**–**13** [30,32], with *trans* configuration for ring junction carbons (7a and 11a) of the tricyclic core, were obtained with moderate to good yields and good to excellent diastereoisomeric excesses (Table 2, entries 1–3).

#### 2.2.2. Synthesis of Lactams with Decahydro-[1,3]oxazino[2,3-j][1,6]naphthyridin-6-one core ([6,6,6] Ring System)

**Scheme 2:**
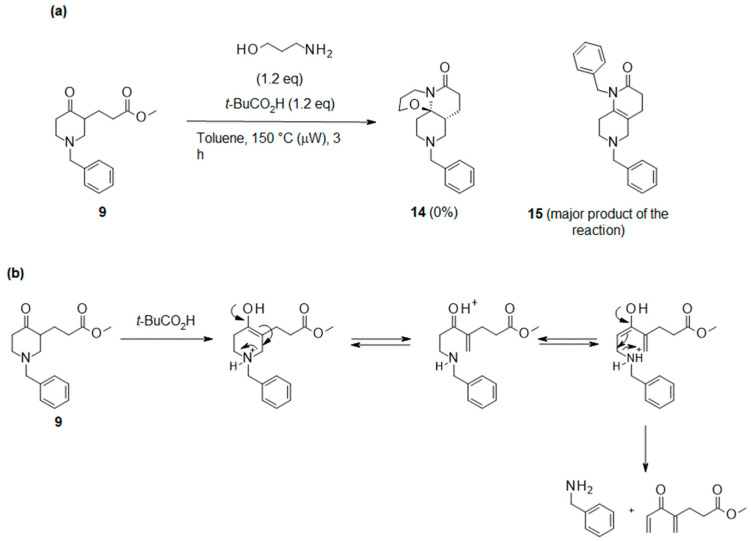

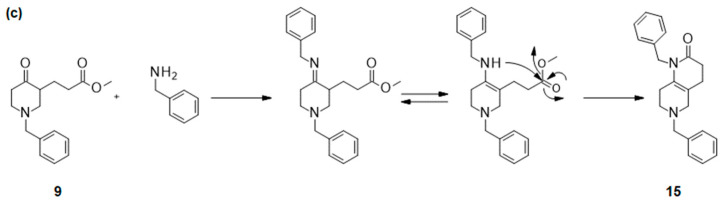
(**a**) Meyers’ lactamization of Benzyl-keto-ester **9** with 3-aminopropanol. Possible pathway for the formation of by-product **15** from keto-ester **9** and benzylamine: (**b**) Hoffmann-type elimination; (**c**) Double condensation of benzylamine on keto-ester **9**.

We next tried to extend this procedure to γ-amino-alcohols to produce derivatives with a decahydro-[1,3]oxazino[2,3-j][1,6]naphthyridin-6-one core ([6,6,6] ring system), in which the previous oxazolidine ring is expanded to an 1,3-oxazinane ring. Benzyl-keto-ester **9** was thus reacted with 3-aminopropanol under the optimized conditions (Scheme 2a).

A complete conversion of **9** was obtained, but only traces of the desired product **14** were observed. Instead, by-product **15** was found to be the major product of the reaction. We propose that **15** is obtained through a double condensation between benzyl-keto-ester **9** and benzylamine (Scheme 2c), which arises from **9** via a double Hoffmann-type elimination in acidic conditions (Scheme 2b) [38].

**Scheme 3:**
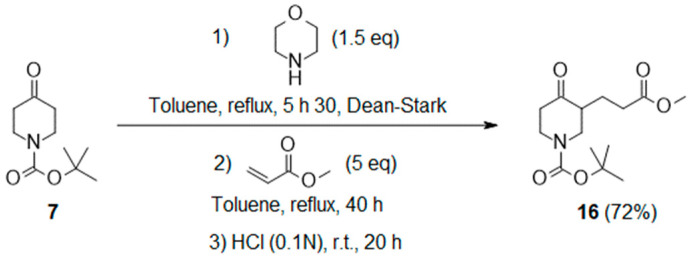
One-pot synthesis of Boc-protected δ-keto-ester **16** via Stork-enamine alkylation.

To limit the formation of by-product **15**, we looked for a non-basic analog of **9** in order to prevent the release of benzylamine by Hoffmann elimination. To this purpose, Boc-protected δ-keto-ester **16** (Scheme 3) was synthesized from commercially available Boc-4-piperidone **7** by Stork-enamine alkylation via a one-pot, three-step process [39,40]. The first step in the sequence is the formation of the enamine by condensation between morpholine and 4-piperidone under toluene reflux using Dean-Stark conditions. Then, the intermediate enamine was alkylated with methyl acrylate, followed by hydrolysis to afford Boc-protected δ-keto-ester **16** in a 72% yield as a racemic mixture.

In contrast to its benzyl analog **9**, Boc-protected δ-keto-ester **16** reacted very efficiently with 3-aminopropanol to produce lactam **17** (Table 3, entry 1). It also reacted efficiently with other C-2 and C-3 substituted aminopropanol derivatives to generate various lactams functionalized on the oxazinane ring with small alkyl groups (compounds **17**–**22**, Table 3, entries 1–6) and spirocycles (compounds **23**–**24**, Table 3, entries 7–8).

Compounds (**17**–**24**) were obtained with good yields ranging from 63% to 98% (Table 3, entries 1, 3–8) except for compound **18,** which was only obtained with an 18% yield (Table 3, entry 2), certainly because of the high steric hindrance of the isopropyl moiety. As previously noticed with compounds **11**–**13** (Table 2, entries 1–3), the stereoselectivity of the reaction was correlated to the steric hindrance of the substituent in the alpha or beta position of the nitrogen. As expected, products were obtained as racemic mixtures when the reaction was performed with non-chiral or racemic amino-alcohols.

#### 2.2.3. Application of Meyers’ Lactamization Reaction to Other Boc-Keto-Esters and Amino-Alcohols to Produce Tricyclic Spirolactams with Original Scaffolds

As shown previously, Meyers’ lactamization of γ-amino-alcohols is more efficient with the Boc-protected δ-keto-ester **16** than with its benzyl analog **9**. This result was confirmed with several β-amino-alcohols. For instance, **16** was reacted with L-valinol using the optimized conditions (Table 4, entry 1), leading to lactam **25** with a higher yield (84%), as compared to lactam **12** obtained from benzyl analog **9** (75%, Table 2, entry 2). However, the stereoselectivity of the reaction was slightly decreased from 100:0 to 90:10 but remained excellent (Table 4, entry 1). Interestingly, we also observed that this reaction can be scaled up to 7 mmol of **16** and can be carried out under reflux of toluene in thermal heating instead of microwave irradiations. A similar yield (88%) of **25** was obtained when the keto-ester **16** was reacted with L-valinol under refluxing toluene (Table 4, entry 1). Therefore, the following examples were performed under these conditions.

Boc-protected δ-keto-ester **16** was then engaged in Meyers’ lactamization reaction with several C-2 substituted β-amino-alcohols. The reaction was also efficient and highly stereoselective, leading to tricyclic spirolactams functionalized on the oxazolidine ring with a benzyl ethyl ether and CF_3_ group (Table 4, entries 2 and 3). The expected products (**26** and **27**) were isolated in moderate to good yields with excellent diastereoisomeric ratios (d.r. > 90:10). Using the optimized conditions, tetracyclic lactam **28** was also obtained from 2-aminophenol with a quantitative yield (Table 4, entry 4).

Interestingly, the scope of the reaction could also be extended to aminobutanol to generate product **29** (Table 4, entry 5) with a dodecahydro-[1,3]oxazepino[2,3-j][1,6]naphthyridine core ([**7**.**6**.**6**] ring system) in 81% yield.

To further explore the scope of the reaction and produce a diversity of tricyclic spirolactams with new scaffolds, we attempted to extend this methodology to different Boc-protected keto-esters. Boc-protected δ-keto-esters **30**, **32**, and **34** (Table 4, entries 6–8) were firstly prepared by Stork-enamine alkylation of *N*-Boc piperidin-4-one, *N*-Boc pyrrolidin-3-one, and *N*-Boc azapan-4-one, respectively, following the protocol previously used for the synthesis of **16** (see Materials and Methods, Section 3.2.1). γ-keto-ester **36** (Table 4, entry 9) was obtained from *N*-Boc piperidin-4-one and methyl-2-bromoacetate using the same strategy. These keto-esters (**30**, **32**, **34**, and **36**) reacted with L-valinol under the optimized conditions. Interestingly, this reaction was efficient, highly stereoselective, and led to novel tricyclic spirolactams **31**, **33**, **35**, and **37** with a decahydrooxazolo[2,3-e][1,5]naphthyridine ([**5**.**6**.**6**] ring system), octahydro-1H-oxazolo[3,2-a]pyrrolo[3,2-b]pyridine ([**5**.**6**.**5**]), decahydro-2H-oxazolo[3′,2′:1,2]pyrido[3,2-c]azepine ([**5**.**6**.**7**]), and octahydro-2H-oxazolo[3′,2′:1,2]pyrrolo[3,2-c]pyridine ([**5**.**5**.**6**]) core, respectively (Table 4, entries 6–9). Therefore, these results showed that this methodology is tractable and allows the modification of the size and substituents of each cycle of tricyclic lactams according to the structure of the keto ester and amino-alcohol used during Meyers’ lactamization reaction.

**Scheme 4:**
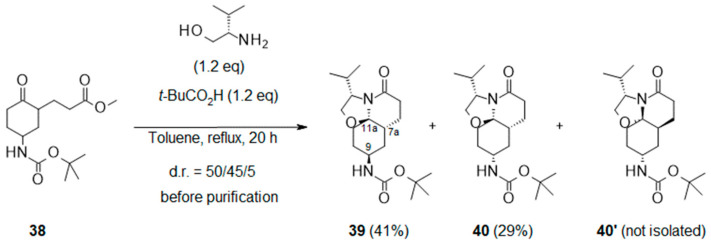
Synthesis of aminocyclohexyl derivative lactams **39** and **40** via Meyers’ lactamization methodology.

To further demonstrate the reliability and versatility of this methodology, Meyers’ lactamization was extended to aminocyclohexanone derivative **38** (Scheme 4), obtained as a mixture of four stereoisomers from *N*-4-Boc-aminocyclohexanone by the previously described Stork-enamine alkylation strategy in a good yield (85%) (see Materials and Methods, Section 3.2.1). As observed for previous substrates, Meyers’ lactamization of δ-ketoester **38** with L-valinol under optimized conditions was highly stereoselective, leading to two main diastereoisomers **39** and **40** (70% overall yield), sharing a *trans* configuration at the ring junction between carbons 7a and 11a, as observed for lactam **25**, but epimers at the C-9 position (Scheme 4).

**Figure 2:**
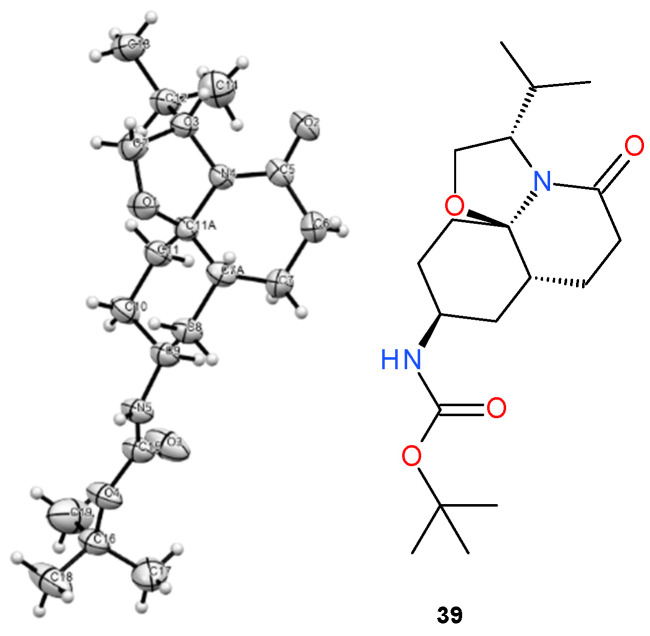
X-ray crystal structure of lactam **39**.

Luckily, **39** and **40** could be separated by flash chromatography. Interestingly, lactam **39** could be crystalized, and X-ray diffraction confirmed the expected configuration of all stereogenic centers and showed the enhanced three-dimensional character of these molecules (Figure 2). It also confirmed the “privileged structure” character of these fused lactams, as the diversification of substituents (R1, R2, and R3) in each ring provides chemical space exploration through three defined and divergent trajectories.

### 2.3. Functionalization of the Lactam Ring

**Scheme 5:**
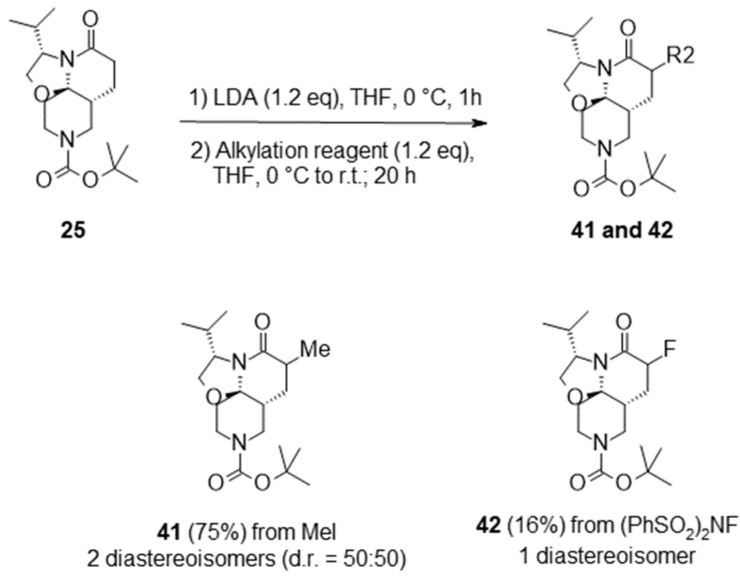
Functionalization of the lactam ring by alkylation reactions.

Having demonstrated the robustness of our methodology for the formation of several tricyclic spirolactam scaffolds with a large diversity of R1 substituents on the oxazolidine ring, we then looked into the functionalization of the remaining two points of diversity (R2 and R3). First, the alkylation of the lactam ring was performed by treating compound **25** with LDA in THF at 0 °C, followed by addition of alkylating reagents (Scheme 5). Use of methyl iodide as an alkylating agent gave methylated product **41** in a 75% yield as a mixture of two diastereoisomers (d.r. = 50:50), separable by flash chromatography. Interestingly, complete stereoselectivity was obtained when *N*-fluorobenzenesulfinimide (NFSI) was used as a fluorinating reagent (Scheme 5). However, the fluorinated analog **42** was only isolated with a 16% yield, due to the formation of a doubly fluorinated derivative that was difficult to separate from **42** by flash chromatography.

### 2.4. Synthesis and Drug-like Properties of Novel Tricyclic Spirolactams Obtained via R3 Position Functionalization

**Scheme 6:**
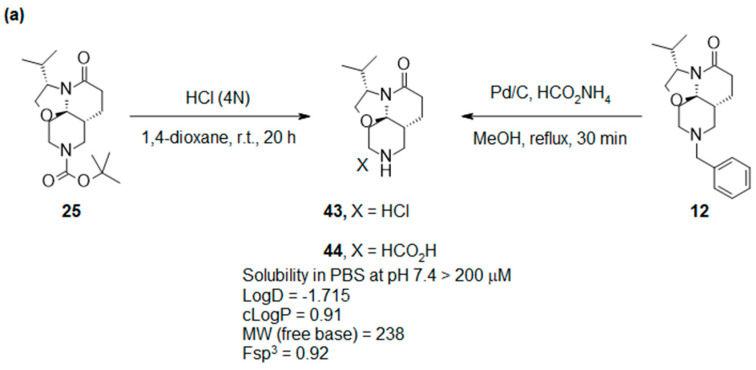

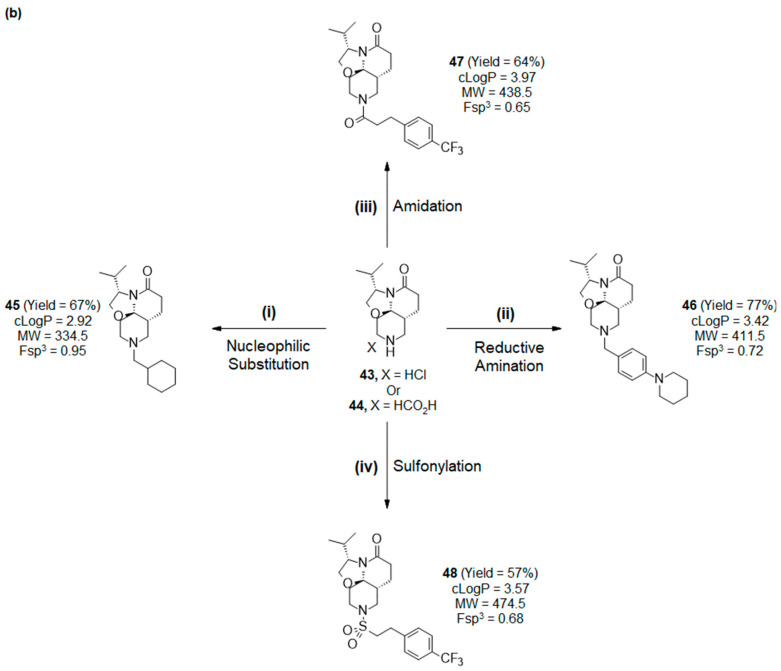
(**a**) Deprotection of lactams **12** and **25** and physico-chemical properties of deprotected compound **44**, (**b**) Selected examples of reaction for the derivatization of lactam **43** and **44** into a focused library of potential drug-like compounds (i) **44** (1 eq), bromomethylcyclohexane (1.1 eq), K_2_CO_3_ (3 eq), NaI (1.1 eq), MeCN, 80 °C, 60 h; (ii) **43** (1 eq), 4-(1-piperidyl)benzaldehyde (1.2 eq), DIEA (1 eq), NaBH(OAc)_3_ (2 eq), 1,2-dichloroethane, r.t., overnight; (iii) **43** (1 eq), 3-[4-(trifluoromethyl)phenyl]propanoic acid (1 eq), DIEA (3 eq), COMU (1.5 eq), ethyl acetate, r.t., 4 h; (iv) **43** (1 eq), 2-[4-(trifluoromethyl)phenyl]ethanesulfonyl chloride (1 eq), MeCN, r.t., overnight.

In order to functionalize the R3 position, the protecting groups (benzyl and Boc) of the piperidine nitrogen of lactams **12** and **25** were both readily removed using Pd-catalyzed hydrogenolysis and acidic conditions to generate the free secondary amines **43** and **44,** respectively (Scheme 6a). The oxazolidine and lactam rings were not altered under these conditions, demonstrating the robust nature of this scaffold.

Interestingly, lactam **44** shows a high aqueous solubility (>200 µM in PBS at pH 7.4) and logD = −1.715 (Scheme 6a). In addition, it exhibits a high fraction of sp^3^-hybridized carbon atoms (Fsp^3^ = 0.9), three contiguous chiral centers, a low molecular weight (<300 g.mol^−1^), and a functional nitrogen atom. Therefore, lactam **44** is an excellent platform for derivatization to produce a library of drug-like compounds for screening against various biological targets.

To this purpose, diverse functional groups were successfully introduced on the piperidine ring via various transformations such as alkylation (nucleophilic substitution, reductive amination), acylation, and sulfonylation. Scheme 6b shows selected examples of such functionalization, leading to compounds **45**, **46**, **47**, and **48**. These molecules are Lipinsky’s rule of five compliant [41] (cLogP calculated using DataWarrior [42]) (Scheme 6b). The Fsp^3^ values of all these compounds (Fsp^3^ > 0.6) are much higher than the average of completely synthetic drugs (Fsp^3^ = 0.37) [9] and are as high as those of natural product drugs (Fsp^3^ = 0.68) [9] (Scheme 6b). These results show the three-dimensionality and drug-like properties of these compounds, suggesting that a screening of our 3D tricyclic spirolactam library against diverse biological targets has the potential to identify hit compounds targeting a new biological space.

## 3. Materials and Methods

### 3.1. General Information

All reagent-grade chemicals and anhydrous solvents for synthesis, analysis, and purification were obtained from commercial suppliers and used as received without further purification.

Flash chromatography was performed using a Puriflash PF-430 with silica gel cartridges (Buchi Silica 40 µm). ELSD and UV detection (254 nm) were used to collect the desired product. Reverse flash chromatography was performed using a CombiFlash^®^ Rf200 with C18 cartridges (Buchi C18 40 µm). UV detection (215 and 254 nm) was used to collect the desired product.

^1^H NMR and ^13^C NMR spectra were recorded on a Bruker DRX-300 spectrometer. Chemical shifts (δ) are in parts per million (ppm). The ^1^H spectra were calibrated to signals from CD_2_Cl_2_ (δ 5.36 ppm) or CDCl_3_ (δ 7.26 ppm), and the ^13^C spectra from CD_2_Cl_2_ (δ 53.84 ppm) or CDCl_3_ (δ 77.16 ppm). ^1^H NMR spectra are reported as the following: chemical shift (ppm), multiplicity (s: singlet; brs: broad singlet; d: doublet; dd: doublet of doublet; t: triplet; td: triplet of doublet; m: multiplet), coupling constants in Hertz (Hz), and integration. Proton and carbon signal assignments were established using COSY, HSQC-DEPT, and HMBC spectra.

The LC-MS Waters system was equipped with a 2747 sample manager, a 2695 separation module, a 2996 photodiode array detector (200–400 nm), and a Micromass ZQ2000 detector (scan 100–800). XBridge C18 column (50 mm × 4.6 mm, 3.5 µm, Waters) was used. The injection volume was 20 µL. A mixture of water and acetonitrile was used as the mobile phase in gradient elution. The pH of the mobile phase was adjusted with HCOOH and NH_4_OH to form a buffer solution at pH 3.8. The analysis time was 5 min (at a flow rate of 2 mL/min), 10 min (at a flow rate of 1 mL/min), or 30 min (at a flow rate of 1 mL/min). Purity (%) was determined by reversed-phase HPLC, using UV detection (215 nm). All final compounds showed purity greater than 95%.

High-resolution mass spectra (HRMS) analysis was performed on a LC-MS system equipped with a LCT Premier XE mass spectrometer (Waters), using an XBridge C18 column (50 mm × 4.6 mm, 3.5 µm, Waters). A gradient starting from 98% H_2_O and 5 mM Ammonium Formate pH 3.8 and reaching 100% MeCN and 5 mM Ammonium Formate pH 3.8 within 3 min at a flow rate of 1 mL was used.

Single-crystal X-ray diffraction measurements were performed at room temperature. Data were collected on a Bruker Apex Duo diffractometer equipped with a Photon III C14 detector and an Incoatec microsource (λ_Cu_ = 1.54184 Å).

### 3.2. Chemistry

**3-(1-benzyl-4-oxo-3-piperidyl)propanoic acid (8):**

Compound **8** was prepared according to the procedure described by our team [32].

**Methyl 3-(1-benzyl-4-oxo-3-piperidyl)propanoate (9):**

To a solution of compound **8** (19.3 g, 73.9 mmol) in methanol (200 mL), SOCl_2_ (5.9 mL, 81.3 mmol) was added dropwise at room temperature. The mixture was then stirred at 55 °C for 1 h. The solvent was removed under vacuum, and the mixture was dissolved in 0.1 N HCl (100 mL) and stirred at room temperature for 1 h. A saturated aqueous solution of Na_2_CO_3_ was added until pH 10. The solution was extracted with ethyl acetate. The organic layer was dried over MgSO_4_, and the solvent was removed under reduced pressure to give the crude product, which was purified by silica gel chromatography (cyclohexane/ethyl acetate: 70/30 to 0/100) to afford **9** (6.73 g, 33%), as a colorless oil. ^1^H NMR (300 MHz, CD_2_Cl_2_): δ 7.36–7.25 (m, 5H), 3.64 (d, *J* = 13.1 Hz, 1H), 3.61 (s, 3H), 3.57 (d, *J* = 13.1 Hz, 1H), 3.08–2.97 (m, 2H), 2.62–2.11 (m, 7H), 2.08–1.95 (m, 1H), 1.53–1.42 (m, 1H) ppm. LCMS (ESI, *m*/*z*): [M+H]^+^ = 276. ^1^H NMR data matched those reported previously [43].

#### 3.2.1. General Protocol 1: Synthesis of Boc-Keto-Esters via Stork Enamine Alkylation

**Scheme 7:**
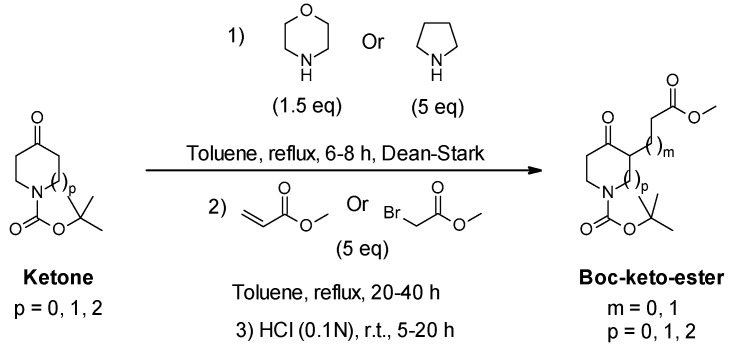
Synthesis of Boc-keto-esters via Stork enamine alkylation.

The appropriate ketone (1 eq) was dissolved in dry toluene (0.4 N), then morpholine (1.5 eq) or pyrrolidine (5 eq) was added. The flask was equipped with a Dean-Stark apparatus and a condenser. The solution was refluxed for 6–8 h. The mixture was cooled down to room temperature, and then methyl acrylate (2.5–5 eq) or methyl-2-bromoacetate (2.5–5 eq) was added. The solution was refluxed for 20–40 h. The mixture was evaporated until dry. The brown oil obtained was dissolved in HCl (10 eq) and stirred at room temperature for 5–20 h. The solution was extracted with ethyl acetate. The layers were separated. The organic layer was dried over MgSO_4_, filtered, and concentrated under reduced pressure to give the crude product, which was purified to afford the corresponding Boc-protected keto-ester, (see Scheme 7).

***Tert*** **-butyl 3-(3-methoxy-3-oxo-propyl)-4-oxo-piperidine-1-carboxylate (16):**

Compound **16** was obtained from *N*-Boc piperidin-4-one, morpholine, and methyl acrylate following the general protocol 1. The crude product was purified by reverse phase chromatography using H_2_O/MeOH (90/10 to 0/100) to afford compound **16** (yield = 72%) as a white solid. ^1^H NMR (300 MHz, CDCl_3_): δ 4.20–4.00 (m, 2H), 3.66 (s, 3H), 3.42–3.26 (m, 1H), 3.06–2.92 (m, 1H), 2.51–2.33 (m, 5H), 2.11–1.98 (m, 1H), 1.65–1.52 (m, 1H),1.48 (s, 9H) ppm. ^13^C (75 MHz, CDCl_3_): δ 209.1, 173.5, 154.6, 80.7, 51.8, 49.4, 48.4, 43.9, 40.9, 31.5, 28.5, 22.5 ppm. HRMS (ESI, *m*/*z*): [M+H]^+^ calcd. For C_14_H_24_NO_5_, 286.1654; found 286.1689.

***Tert*** **-*butyl* 2-(3-methoxy-3-oxo-propyl)-3-oxo-piperidine-1-carboxylate (30):**

Compound **30** was obtained from *N*-Boc piperidin-4-one, morpholine, and methyl-2-bromoacetate using general protocol 1. The crude product was purified by reverse phase chromatography using H_2_O/MeOH (90/10 to 0/100) to afford **30** (yield = 60%) as a colorless oil. ^1^H NMR (300 MHz, CDCl_3_): δ 3.65 (s, 3H), 3.29–3.20 (m, 2H), 2.63–2.55 (m, 2H), 2.49–2.33 (m, 3H), 2.09–1.90 (m, 5H), 1.44 (s, 9H) ppm. HRMS (ESI, *m*/*z*): [M+H]^+^ calcd. for C_14_H_24_NO_5_, 286.1654; found 286.1668.

***Tert*** **-butyl 3-(3-methoxy-3-oxo-propyl)-4-oxo-pyrrolidine-1-carboxylate (32):**

Compound **32** was obtained from *N*-Boc pyrrolidin-3-one, morpholine, and methyl acrylate following the general protocol 1. The crude product was purified by reverse-phase chromatography using H_2_O/MeOH (90/10 to 0/100) to afford **32** (yield = 43%) as a colorless oil. ^1^H NMR (300 MHz, CDCl_3_): δ 3.96–3.98 (m, 1H), 3.73–3.65 (m, 1H), 3.63 (s, 3H), 3.57–3.44 (m, 1H), 2.67–2.23 (m, 4H), 2.20–2.01 (m, 2H), 1.47 (s, 9H) ppm. LCMS (ESI, *m*/*z*): [M+H]^+^ = 272.

***Tert*** **-butyl 3-(3-methoxy-3-oxo-propyl)-4-oxo-azepane-1-carboxylate (34):**

Compound **34** was obtained from *N*-Boc azapan-4-one, pyrrolidine, and methyl acrylate using general protocol 1. The crude product was purified by reverse phase chromatography using H_2_O/MeOH (90/10 to 0/100) to afford keto-ester **34** (yield = 34%) as a colorless oil. ^1^H NMR (300 MHz, CDCl_3_): δ 4.02–3.79 (m, 2H), 3.64 (s, 3H), 3.11–2.93 (m, 2H), 2.91–2.73 (m, 1H), 2.72–2.55 (m, 2H), 2.52–2.42 (m, 1H), 2.40–2.24 (m, 2H), 1.82–1.61 (m, 3H), 1.42 (s, 9H) ppm. ^13^C (75 MHz, CDCl_3_): δ 212.9, 173.4, 154.5, 80.3, 77.3, 51.5, 49.3, 42.8, 40.8, 31.6, 28.4, 26.6, 25.8, 25.6, 24.5, 24.4, 23.5 ppm. HRMS (ESI, *m*/*z*): [M+H]^+^ calcd. For C_15_H_26_NO_5_, 300.1811; found 300.1827.

***Tert*** **-butyl 3-(2-methoxy-2-oxo-ethyl)-4-oxo-piperidine-1-carboxylate (36):**

Compound **36** was obtained from *N*-Boc piperidin-4-one, morpholine, and methyl-2-bromoacetate using general protocol 1. The crude product was purified by reverse phase chromatography using H_2_O/MeOH (90/10 to 0/100) to afford **36** (yield = 9%) as a colorless oil. ^1^H NMR (300 MHz, CDCl_3_): δ 4.40–4.24 (m, 2H), 3.70 (s, 3H), 3.25–3.211 (m, 1H), 3.02–2.84 (m, 2H), 2.77–2.64 (m, 1H), 2.62–3.37 (m, 2H), 2.34–2.22 (m, 1H),1.50 (s, 9H) ppm; ^13^C (75 MHz, CDCl_3_): δ 207.5, 172.1, 154.5, 80.8, 52.0, 48.1, 46.4, 43.8, 41.0, 31.3, 28.5 ppm. HRMS (ESI, *m*/*z*): [M+H]^+^ calcd. for C_13_H_22_NO_5_, 272.1498; found 272.1501.

**Methyl 3-[5-(tert-butoxycarbonylamino)-2-oxo-cyclohexyl]propanoate (38):**

Compound **38** was obtained from *N*-Boc cyclohexan-4-one, pyrrolidine, and methyl acrylate using general protocol 1. The crude product was purified by flash column chromatography over silica gel (cyclohexane/ethyl acetate 100/0 to 40/60) to afford the desired keto-ester **38** (yield = 85%) as a yellow oil. ^1^H NMR (300 MHz, CDCl_3_): δ 4.85–4.49 (1H), 4.00 (br s, 1H), 3.67 (s, 3H), 2.61–2.25 (m, 7H), 2.17–1.98 (m, 4H), 1.48–1.43 (m, 9H) ppm. LCMS (ESI, *m*/*z*): [M+H]^+^ = 300.

#### 3.2.2. General Protocol 2: Synthesis of Lactams via Meyers’ Lactamization of Keto-Esters with Amino-Alcohols

**Scheme 8:**
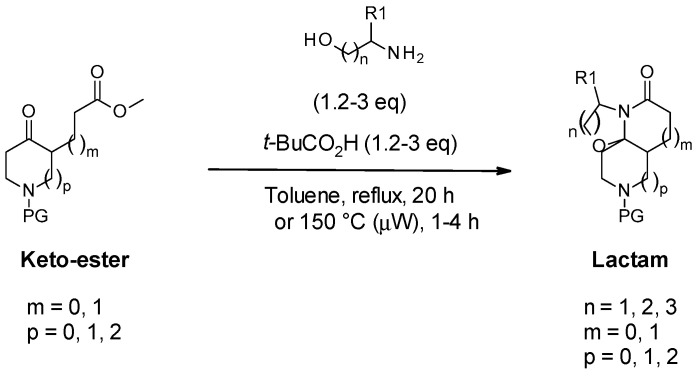
Synthesis of lactams via Meyers’ lactamization of keto-esters with amino-alcohols.

A solution of pivalic acid (1.2–3 eq) in toluene (0.2 N) was added to the appropriate keto-ester (1 eq). The appropriate amino-alcohol (1.2–3 eq) was added (when the amine is used as a chlorohydrate, DIEA (1.2–3 eq) was added). The mixture was refluxed (thermic for 20 h or 150 °C under microwave irradiations for 1–4 h). When the conversion of the keto-ester was judged complete by LC/MS, the solution was dissolved in H_2_O and extracted with ethyl acetate or dichloromethane. The layers were separated. The organic layer was dried over MgSO_4_, filtered, and concentrated under vacuum to give the crude product, which was purified to afford the desired product, (see Scheme 8).

(***3S,7aS,11aS*)-9-benzyl-3-methyl-2,3,6,7,7a,8,10,11-octahydrooxazolo[2,3-j][1,6]naphthyridin-5-one (11) and (*3S,7aS,11aS*)-9-benzyl-3-methyl-2,3,6,7,7a,8,10,11-octahydrooxazolo[2,3-j][1,6]naphthyridin-5-one (11′):**

The products **11** and **11′** were obtained from keto-ester **9** and (*2S*)-2-aminopropan-1-ol following the general protocol 2. The crude product was purified by preparative HPLC using H_2_O + 0.1% HCOOH/MeCN + 0.1% HCOOH (90/10 to 0/100) to afford 11 and **11′** (yield = 46%) as a yellow oil, as a mixture of two diastereoisomers (d.r. = 70:30). Data for the major diastereoisomer **11**: ^1^H NMR (300 MHz, CD_2_Cl_2_): δ 7.38–7.20 (m, 5H), 4.28–4.06 (m, 2H), 3.62 (dd, *J* = 13.0 Hz, *J* = 1.3 Hz, 1H), 3.53 (d, *J* = 13.4 Hz, 1H), 3.42 (d, *J* = 13.4 Hz, 1H), 2.78–2.63 (m, 2H), 2.58–2.15 (m, 5H), 1.99–1.60 (m, 5H), 1.29 (d, *J* = 6.1 Hz, 3H) ppm. ^13^C NMR (75 MHz, CD_2_Cl_2_): δ 168.9, 139.4, 129.0, 128.5, 127.3, 92.6, 69.7, 62.7, 55.2, 54.7, 51.7, 50.9, 40.7, 31.6, 31.1, 22.9, 20.2 ppm. HRMS (ESI, *m*/*z*): [M+H]^+^ calcd. for C_18_H_25_N_2_O_2_, 301.1916; found: 301.1914.

**(*3S,7aR,11aR*)-9-benzyl-3-isopropyl-2,3,6,7,7a,8,10,11-octahydrooxazolo[2,3-j][1,6]naphthyridin-5-one (12):**

Product **12** was obtained from keto-ester **9** and L-valinol using general protocol 2. The crude product was purified by flash column chromatography on silica gel (Cyclohexane/ethyl acetate: 100/0 to 0/100) to afford compound **12** (yield = 75%, white powder) as a single diastereoisomer. ^1^H NMR (300 MHz, CDCl_3_): δ 7.33–7.20 (m, 5H), 4.13–4.03 (m, 1H), 3.98 (dd, *J* = 8.6 Hz, *J* = 7.6 Hz, 1H), 3.77 (dd, *J* = 8.6 Hz, *J* = 6.2 Hz, 1H), 3.54 (d, *J* = 13.3 Hz, 1H), 3.41 (d, *J* = 13.1 Hz, 1H), 2.77–2.64 (m, 2H), 2.62–2.51 (m, 1H), 2.49–2.29 (m, 3H), 2.25–2.16 (m, 1H), 2.09–1.89 (m, 2H), 1.83–1.73 (m, 1H), 1.72–1.55 (m, 2H), 0.94 (d, *J* = 6.8 Hz, 3H), 0.90 (d, *J* = 6.8 Hz, 3H) ppm. ^13^C NMR (75 MHz, CDCl_3_): δ 170.2, 138.9, 128.7, 128.3, 127.0, 92.7, 66.2, 62.5, 61.2, 54.8, 50.7, 40.4, 32.4, 32.4, 30.8, 21.9, 19.9, 18.7 ppm. HRMS (ESI, *m*/*z*): [M+H]^+^ calculated for C_20_H_29_N_2_O_2_, 329.2229; found 329.2227.

**(*3S,7aR,11aR*)-9-benzyl-3-tert-butyl-2,3,6,7,7a,8,10,11-octahydrooxazolo[2,3-j][1,6]naphthyridin-5-one (13):**

Product **13** was obtained from keto-ester **9** and (*2S*)-2-amino-3,3-dimethyl-butan-1-ol using general protocol 2. The crude was purified by preparative HPLC using H_2_O + 0.1% HCOOH/MeCN + 0.1% HCOOH (100/0 to 0/100) to afford the desired product **13** (yield = 40%, yellow oil) as a single diastereoisomer. ^1^H NMR (300 MHz, CD_2_Cl_2_): δ 7.36–7.19 (m, 5H), 4.11 (t, *J* = 6.9 Hz, 1H), 3.85 (d, *J* = 6.5 Hz, 2H), 3.53 (d, *J* = 13.3 Hz, 1H), 3.41 (d, *J* = 13.3 Hz, 1H), 2.77–2.69 (m, 1H), 2.61–2.57 (m, 1H), 2.58–2.49 (m, 1H), 2.45–2.27 (m, 3H), 2.20 (dt, *J* = 12.0 Hz, *J* = 3.4 Hz, 1H), 1.98 (dt, *J* = 13.3 Hz, *J* = 4.68 Hz, 1H), 1.90–1.82 (m, 1H), 1.73–1.52 (m, 2H), 0.92 (s, 9H) ppm. ^13^C (75 MHz, CD_2_Cl_2_): δ 172.7, 139.5, 129.1, 128.5, 127.2, 94.3, 64.8, 64.5, 62.7, 54.9, 50.9, 40.2, 34.8, 32.7, 31.0, 27.8, 20.9 ppm. HRMS (ESI, *m*/*z*): [M+H]^+^ calcd. For C_21_H_31_N_2_O_2_, 343.2386; found: 343.2394.

***Tert*** **-butyl 6-oxo-3,4,7,8,8a,9,11,12-octahydro-2H-[1,3] oxazino[2,3-j][1,6]naphthyridine-10-carboxylate (17):**

Product **17** was obtained from keto-ester **16** and 3-aminopropan-1-ol using general protocol 2. The crude product was purified by flash column chromatography on silica gel (cyclohexane/ethyl acetate: 100/0 to 0/100) to afford **17** (yield = 79%, white powder) as a racemic mixture. ^1^H NMR (300 MHz, CDCl_3_): δ 4.61 (dd, *J* = 13.9 Hz, *J* = 4.9 Hz, 1H), 4.15–3.73 (m, 4H), 3.35–3.15 (m, 1H), 2.90–2.69 (m, 2H), 2.58 (d, *J* = 13.9 Hz, 1H), 2.51–2.42 (m, 2H), 1.98–1.66 (m, 4H), 1.64–1.50 (m, 2H), 1.42 (s, 9H) ppm. ^13^C (75 MHz, CDCl_3_): δ 168.2, 155.0, 85.8, 79.8, 59.6, 45.1, 41.7, 39.7, 34.2, 31.8, 28.5, 26.4, 25.2, 21.3 ppm. HRMS (ESI, *m*/*z*): [M+H]^+^ calculated for C_16_H_27_N_2_O_4_, 311.1971; found 311.1985.

***Tert*-butyl (*4R,8aR,12aR*)-4-isopropyl-6-oxo-3,4,7,8,8a,9,11,12-octahydro-2H-[1,3]oxazino[2,3-j][1,6]naphthyridine-10-carboxylate (18):**

Product **18** was obtained from keto-ester **16** and (*3R*)-3-amino-4-methyl-pentan-1-ol using general protocol 2. The crude product was purified by flash column chromatography on silica gel (cyclohexane/ethyl acetate: 100/0 to 0/100) to give compound **18** (yield = 18%, colorless oil) as a single diastereoisomer. ^1^H NMR (300 MHz, CDCl_3_): δ 4.64–4.54 (m, 1H), 4.10–3.76 (m, 3H), 3.73–3.63 (m, 1H), 3.40–3.22 (m, 1H), 2.92–2.73 (m, 1H), 2.62–2.30 (m, 3H), 2.04–1.59 (m, 7H), 1.44 (s, 9H), 0.98 (d, *J* = 6.3 Hz, 3H), 0.87 (d, *J* = 6.3 Hz, 3H) ppm. ^13^C (75 MHz, CDCl_3_): δ 169.7, 155.0, 86.3, 79.8, 56.3, 51.7, 45.5, 42.3, 39.9, 32.3, 31.7, 30.3, 28.5, 26.6, 21.2, 20.7, 19.6 ppm. LCMS (ESI, *m*/*z*): [M+H]^+^ = 353.

***Tert*** **-butyl (*4S,8aR,12aR*)-4-methyl-6-oxo-3,4,7,8,8a,9,11,12-octahydro-2H-[1,3]oxazino[2,3-j][1,6]naphthyridine-10-carboxylate (19) and tert-butyl (4S,8aS,12aS)-4-methyl-6-oxo-3,4,7,8,8a,9,11,12-octahydro-2H-[1,3]oxazino[2,3-j][1,6]naphthyridine-10-carboxylate (19′):**

Products **19** and **19**′ were obtained from keto-ester **16** and 3-aminobutan-1-ol using general protocol 2. The crude product was purified by flash column chromatography on silica gel (cyclohexane/ethyl acetate: 100/0 to 0/100) to give compound **19** + **19**′ (yield = 86%, colorless oil) as a racemic mixture of two diastereoisomers (d.r. = 50:50). Data for the mixture of diastereoisomers **19** and **19**′: ^1^H NMR (300 MHz, CDCl_3_): δ 4.88–4.76 (m, 1H), 4.53–4.42 (m, 1H), 4.21–3.85 (m, 5H), 3.76 (dd, *J* = 5.0 Hz, *J* = 2.5 Hz, 1H), 3.71 (dd, *J* = 5.1 Hz, *J* = 2.5 Hz, 1H), 3.38–3.18 (m, 2H), 2.91–2.69 (m, 2H), 2.53–2.37 (m, 5H), 2.29–1.97 (m, 3H), 1.89–1.61 (m, 9H), 1.43 (s, 9H), 1.42 (s, 9H), 1.31 (d, *J* = 6.6 Hz, 3H), 1.29 (d, *J* = 7.2 Hz, 3H), 1.21 (d, *J* = 9.2 Hz, 4H) ppm. ^13^C (75 MHz, CDCl_3_): δ 168.6, 168.2, 155.2, 155.0, 85.9, 85.3, 79.9, 79.8, 56.5, 55.7, 45.2, 44.7, 43.9, 41.9, 41.7, 40.7, 39.8, 32.0, 31.3, 30.8, 29.8, 29.5, 29.1, 28.5, 37.3, 26.7, 21.1, 20.8, 20.1, 19.1 ppm. HRMS (ESI, *m*/*z*): [M+H]^+^ calculated for C_17_H_29_N_2_O_5_, 325.2127; found 325.2139.

***Tert*** **-butyl (*3R,8aR,12aR*)-3-methyl-6-oxo-3,4,7,8,8a,9,11,12-octahydro-2H-[1,3]oxazino[2,3-j][1,6]naphthyridine-10-carboxylate (20):**

Product **20** was obtained from keto-ester **16** and 3-amino-2-methyl-propan-1-ol using general protocol 2. The crude product was purified by flash column chromatography on silica gel (cyclohexane/ethyl acetate: 100/0 to 0/100) to give compound **20** (yield = 63%, colorless oil) as a single diastereoisomer (in mixture with its enantiomer). ^1^H NMR (300 MHz, CDCl_3_): δ 4.60 (ddd, *J* = 13.7 Hz, *J* = 4.9 Hz, *J* = 1.8 Hz, 1H), 4.13–3.79 (m, 2H), 3.74 (ddd, *J* = 11.7 Hz, *J* = 4.9 Hz, *J* = 1.8 Hz, 1H), 3.49–3.20 (m, 2H), 2.90–2.68 (m, 1H), 2.62–2.37 (m, 4H), 1.94–1.67 (m, 4H), 1.57 (ddd, *J* = 13.7 Hz, *J* = 4.9 Hz, *J* = 1.5 Hz, 1H), 1.44 (s, 9H), 0.84 (d, *J* = 6.7 Hz, 3H) ppm. ^13^C (75 MHz, CDCl_3_): δ 168.1, 155.0, 85.2, 79.9, 65.9, 45.3, 41.7, 41.2, 39.7, 31.8, 30.0, 28.5, 26.2, 21.4, 14.4 ppm. HRMS (ESI, *m*/*z*): [M+H]^+^ calculated for C_17_H_29_N_2_O_5_, 325.2127; found 325.2136.

***Tert*** **-butyl (*3R,8aR,12aR*)-3-ethyl-6-oxo-3,4,7,8,8a,9,11,12-octahydro-2H-[1,3]oxazino[2,3-j][1,6]naphthyridine-10-carboxylate (21) and *Tert*-butyl (*3R,8aR,12aR*)-3-ethyl-6-oxo-3,4,7,8,8a,9,11,12-octahydro-2H-[1,3]oxazino[2,3-j][1,6]naphthyridine-10-carboxylate (21′):**

Products **21** and **21**′ were obtained from keto-ester **16** and 2-(aminomethyl)butan-1-ol using general protocol 2. The crude product was purified by flash column chromatography on silica gel (cyclohexane/ethyl acetate: 100/0 to 0/100) to give compounds **21** + **21**′ (yield = 89%, colorless oil) as a racemic mixture of two diastereoisomers (d.r. = 70:30). Data for the major compound **21**: ^1^H NMR (300 MHz, CDCl_3_): δ 4.65 (ddd, *J* = 13.6 Hz, *J* = 4.9 Hz, *J* = 1.8 Hz, 1H), 4.13–3.89 (m, 2H), 3.81 (ddd, *J* = 13.6 Hz, *J* = 4.9 Hz, *J* = 1.8 Hz, 1H), 3.51–3.40 (m, 1H), 3.38–3.19 (m, 1H), 2.80–2.67 (m, 1H), 2.63–2.37 (m, 5H), 1.87–1.63 (m, 4H), 1.62–1.54 (m, 1H), 1.43 (s, 9H), 1.27–1.14 (m, 2H), 0.92 (t, *J* = 7.2 Hz, 3H) ppm. ^13^C (75 MHz, CDCl_3_): δ 168.1, 155.0, 85.4, 79.8, 64.7, 45.3, 41.7, 39.7, 37.5, 36.4, 31.8, 28.5, 26.2, 23.0, 21.3, 11.0 ppm. HRMS (ESI, *m*/*z*): [M+H]^+^ calculated for C_18_H_31_N_2_O_4_, 339.2284; found 339.2312.

***Tert*** **-butyl 3-isopropyl-6-oxo-3,4,7,8,8a,9,11,12-octahydro-2H-[1,3]oxazino[2,3-j][1,6]naphthyridine-10-carboxylate (22):**

Product **22** was obtained from keto-ester **16** and 2-(aminomethyl)-3-methyl-butan-1-ol using general protocol 2. The crude product was purified by flash column chromatography on silica gel (cyclohexane/ethyl acetate: 100/0 to 0/100) to give compound **22** (Yield = 92%, colorless oil) as a racemic mixture of a single diastereoisomer. ^1^H NMR (300 MHz, CDCl_3_): δ 4.74–4.64 (m, 1H), 4.13–3.73 (m, 3H), 3.64–3.20 (m, 1H), 2.98–2.71 (m, 1H), 2.57–2.45 (m, 4H), 1.87–1.67 (m, 3H), 1.64–1.51 (m, 4H), 1.45 (s, 9H); 0.95 (d, *J* = 6.8 Hz, 3H), 0.89 (d, *J* = 6.8 Hz, 3H) ppm. ^13^C (75 MHz, CDCl_3_): δ 168.6, 155.1, 85.2, 79.9, 63.3, 61.8, 45.4, 41.7, 40.9, 38.8, 36.6, 31.9, 29.8, 28.5, 21.3, 20.8, 20.0, 19.9 ppm. HRMS (ESI, *m*/*z*): [M+H]^+^ calculated for C_19_H_33_N_2_O_4_, 353.2440; found 353.2439.

***Tert*** **-butyl (*8aR,12aR*)-6-oxospiro[2,4,7,8,8a,9,11,12-octahydro-[1,3]oxazino[2,3-j][1,6]naphthyridine-3,1′-cyclopropane]-10-carboxylate (23):**

Product **23** was obtained from keto-ester **16** and [1-(aminomethyl)cyclopropyl]methanol using general protocol 2. The crude product was purified by flash column chromatography on silica gel (cyclohexane/ethyl acetate: 100/0 to 0/100) to give compound **23** (yield = 88%, colorless oil) as a racemic mixture. ^1^H NMR (300 MHz, CDCl_3_): δ 4.20 (d, *J* = 11.7 Hz, 1H), 4.14–3.88 (m,1H), 3.84 (dd, *J* = 13.8 Hz, *J* =1.8 Hz, 1H), 3.38–3.29 (m, 1H), 3.24 (d, *J* = 13.7 Hz, 1H), 2.96 (dd, *J* = 11.7 Hz, *J* =1.8 Hz, 1H), 2.90–2.71 (m, 1H), 2.64 (d, *J* =14.6 Hz, 1H), 2.52–2.46 (m, 2H), 1.97–1.56 (m, 5H), 1.43 (s, 9H), 0.63–0.54 (m, 1H), 0.53–0.43 (m, 2H), 0.42–0.33 (m, 1H). ^13^C (75 MHz, CDCl_3_): δ 168.3, 155.0, 85.8, 79.9, 67.1, 45.3, 42.2, 41.6, 39.7, 31.7, 28.5, 26.5, 21.4, 17.7, 13.4, 6.6 ppm. HRMS (ESI, *m*/*z*): [M+H]^+^ calculated for C_18_H_29_N_2_O_4_, 337.2127; found 337.2142.

***Tert*** **-butyl (*8aR,12aR*)-6-oxospiro[2,4,7,8,8a,9,11,12-octahydro-[1,3]oxazino[2,3-j][1,6]naphthyridine-3,1′-cyclobutane]-10-carboxylate (24):**

Product **24** was obtained from keto-ester **16** and [1-(aminomethyl)cyclobutyl]methanol following the general protocol 2. The crude product was purified by flash column chromatography on silica gel (cyclohexane/ethyl acetate: 100/0 to 0/100) to give compound **24** (yield = 98%, white powder) as a racemic mixture. ^1^H NMR (300 MHz, CDCl_3_): δ 4.62 (dd, *J* = 13.5 Hz, *J* = 1.4 Hz, 1H), 4.11–3.74 (m, 2H), 3.70–3.59 (m, 2H), 3.35–3.19 (m, 1H), 2.65 (d, *J* = 13.5 Hz, 1H), 2.53–2.42 (m, 3H), 2.34–2.10 (m, 1H), 1.99–1.44 (m, 10H), 1.41 (s, 9H) ppm. ^13^C (75 MHz, CDCl_3_): δ 168.6, 154.9, 85.2, 79.7, 68.5, 45.2, 43.8, 41.5, 39.7, 37.4, 31.6, 29.7, 28.4, 26.3, 25.9, 21.2, 15.0 ppm. HRMS (ESI, *m*/*z*): [M+H]^+^ calculated for C_19_H_31_N_2_O_4_, 351.2284; found 351.2290.

***Tert*** **-butyl (*3S,7aR,11aR*)-3-isopropyl-5-oxo-2,3,6,7,7a,8,10,11-octahydrooxazolo[2,3-j][1,6]naphthyridine-9-carboxylate (25):**

Product **25** was obtained from keto-ester **16** and L-valinol using general protocol 2. The crude product was purified by flash column chromatography on silica gel (cyclohexane/ethyl acetate: 100/0 to 0/100) to give **25** (yield = 88%, white solid), as a single diastereoisomer. ^1^H NMR (300 MHz, CDCl_3_): δ 4.18–4.04 (m, 2H), 4.02–3.90 (m, 2H), 3.78 (dd, *J* = 8.7, 6.1 Hz, 1H), 3.30–3.11 (m, 1H), 2.97–2.78 (m, 1H), 2.62–2.50 (m, 1H), 2.39–2.34 (m, 1H), 2.09–1.93 (m, 1H), 1.89–1.54 (m, 5H), 1.44 (s, 9H), 0.92 (d, *J* = 6.9 Hz, 3H), 0.89 (d, *J* = 6.9 Hz, 3H) ppm. ^13^C (75 MHz, CDCl_3_): δ 169.8, 155.1, 92.4, 79.9, 66.4, 61.5, 45.5, 44.6, 40.2, 32.3, 31.9, 30.3, 28.5, 20.6, 19.8, 18.7 ppm. HRMS (ESI, *m*/*z*): [M+H]^+^ calculated for C_18_H_31_N_2_O_4_, 339.2284; found 329.2305.

***Tert*** **-butyl (*3R,7aR,11aR*)-3-[(1R)-1-benzyloxyethyl]-5-oxo-2,3,6,7,7a,8,10,11-octahydrooxazolo[2,3-j][1,6]naphthyridine-9-carboxylate (26):**

Product **26** was obtained from keto-ester **16** and (2R,3R)-2-amino-3-benzyloxy-butan-1-ol;hydrochloride using general protocol 2. The crude product was purified by flash column chromatography on silica gel (cyclohexane/ethyl acetate: 100/0 to 0/100) to give **26** (yield = 50%, colorless oil) as a mixture of two diastereoisomers (d.r. = 94:6). Data for the major compound **26**: ^1^H NMR (300 MHz, CDCl_3_): δ 7.31–7.18 (m, 5H), 4.57 (d, *J* = 11.8 Hz, 1H), 4.41 (d, *J* = 11.8 Hz, 1H), 4.44–4.34 (m, 1H), 4.07–3.83 (m, 5H), 3,23–3.04 (m, 1H), 2.86–2.67 (m, 1H), 2.54–2.30 (m, 2H), 1.86–1.49 (m, 5H), 1.38 (s, 9H), 1.10 (d, *J* = 6.5 Hz, 3H) ppm. ^13^C (75 MHz, CDCl_3_): δ 170.2, 155.0, 138.6, 128.4, 127.7, 92.9, 79.8, 74.2, 71.4, 64.3, 59.0, 45.4, 41.3, 40.4, 30.1, 28.5, 20.5, 15.4 ppm. HRMS (ESI, *m*/*z*): [M+H]^+^ calculated for C_24_H_35_N_2_O_5_, 431.2546; found 431.2551.

***Tert*** **-butyl (*3R,7aR,11aR*)-5-oxo-3-(trifluoromethyl)-2,3,6,7,7a,8,10,11-octahydrooxazolo[2,3-j][1,6]naphthyridine-9-carboxylate (27):**

Product **27** was obtained from keto-ester **16** and (*2R*)-2-amino-3,3,3-trifluoro-propan-1-ol;hydrochloride using general protocol 2. The crude product was purified by flash column chromatography on silica gel (cyclohexane/ethyl acetate: 100/0 to 0/100) to afford **27** (yield = 59%, colorless oil), as a single diastereoisomer. ^1^H NMR (300 MHz, CDCl_3_): δ 4.98–4.84 (m, 1H), 4.25–3.94 (m, 4H), 3.29–3.12 (m, 1H), 2.96–2.69 (m, 1H), 2.70–2.58 (m, 1H), 2.56–2.42 (m, 1H), 1.92–1.75 (m, 4H), 1.71–1.58 (m, 1H), 1.45 (m, 9H) ppm. ^13^C (75 MHz, CDCl_3_): δ 170.6, 155.1, 124.2 (q, *J* = 280.2 Hz), 95.0, 80.2, 63.0 (q, *J* = 2.0 Hz), 56.3 (q, *J* = 33.9 Hz), 45.4, 41.2, 40.4, 30.6, 30.4, 28.6, 20.5 ppm. LCMS (ESI, *m*/*z*): [M+H]^+^ = 365.

***Tert*** **-butyl 9-oxo-17-oxa-4,10-diazatetracyclo[8.7.0.01,6.011,16]heptadeca-11(16),12,14-triene-4-carboxylate (28):**

Product **28** was obtained from keto-ester **16** and 2-aminophenol using general protocol 2. The crude product was purified by flash column chromatography on silica gel (cyclohexane/ethyl acetate: 100/0 to 0/100) to afford **28** (yield = 98%, brown oil) as a racemic mixture. ^1^H NMR (300 MHz, CDCl_3_): δ 7.81 (dd, *J* = 7.5 Hz, *J* = 1.1 Hz, 1H), 7.03 (td, *J* = 7.7 Hz, *J* = 1.4 Hz, 1H), 6.90 (td, *J* = 7.7 Hz, *J* = 1.4 Hz, 1H), 6.86 (dd, *J* = 7.7 Hz, *J* = 1.1 Hz, 1H), 4.28–4.03 (m, 1H), 3.48–3.30 (m, 1H), 3.15–2.98 (m, 1H), 2.75–2.51 (m, 2H), 2.27–2.14 (m, 1H), 2.05–1.67 (m, 4H), 1.48 (s, 9H) ppm. ^13^C (75 MHz, CDCl_3_): δ 166.4, 155.1, 148.9, 129.7, 125.3, 121.9, 117.4, 109.7, 97.7, 80.3, 53.6, 45.41, 38.1, 32.0, 30.3, 28.6, 21.1 ppm. LCMS (ESI, *m*/*z*): [M+H]^+^ = 345.

***Tert*** **-butyl (*1R,6R*)-9-oxo-15-oxa-4,10-diazatricyclo[8.5.0.01,6]pentadecane-4-carboxylate (29):**

Product **29** was obtained from keto-ester **16** and [4-aminobutan-1-ol following the general protocol 2. The crude product was purified by flash column chromatography on silica gel (cyclohexane/ethyl acetate: 100/0 to 0/100) to give compound **29** (yield = 81%, colorless oil), as a racemic mixture. ^1^H NMR (300 MHz, CDCl_3_): δ 4.14–3.84 (m, 3H), 3.79–3.71 (m, 1H), 3.57–3.47 (m, 1H), 3.27–3.07 (m, 1H), 2.92 (dd, *J* = 11.0 Hz, *J* = 12.3 Hz, 1H), 2.56–2.37 (m, 2H), 1.87–1.59 (m, 10H), 1.44 (s, 9H) ppm. ^13^C (75 MHz, CDCl_3_): δ 69.7, 155.0, 88.5, 79.8, 62.6, 45.3, 41.2, 40.4, 37.0, 33.2, 31.7, 29.5, 28.5, 26.5, 21.7 ppm. HRMS (ESI, *m*/*z*): [M+H]^+^ calculated for C_17_H_29_N_2_O_4_, 325.2127; found 325.2139.

***Tert*** **-butyl (*3S,7aS,11aR*)-3-isopropyl-5-oxo-2,3,6,7,7a,9,10,11-octahydrooxazolo[2,3-e][1,5]naphthyridine-8-carboxylate (31):**

Compound **31** was obtained from keto-ester **30** and L-valinol using general protocol 2. The crude was purified by flash column chromatography on silica gel (cyclohexane/ethyl acetate: 100/0 to 0/100) to give product **31** (yield = 49%, colorless oil), as a single diastereoisomer. ^1^H NMR (300 MHz, CDCl_3_): δ 4.21–3.87 (m, 4H), 3.72 (dt, *J* = 5.5 Hz, *J* = 8.9 Hz, 1H), 2.89–2.38 (m, 3H), 2.14–1.86 (m, 3H), 1.81–1.67 (m, 3H), 1.59–1.49 (m, 1H), 1.42 (s, 9H), 0.91 (d, *J* = 7.1 Hz, 3H), 0.89–0.85 (m, 3H) ppm. ^13^C (75 MHz, CDCl_3_): δ 169.1, 155.0, 91.2, 90.2, 66.4, 61.9, 55.3, 54.1, 38.7, 37.5, 32.4, 30.3, 30.0, 28.4, 21.4, 19.7, 19.6, 18.9 ppm. HRMS (ESI, *m*/*z*): [M+H]^+^ calculated for C_18_H_31_N_2_O_4_, 339.2284; found 339.2299.

***Tert*** **-butyl (*1S,4S,9R*)-4-isopropyl-6-oxo-2-oxa-5,11-diazatricyclo[7.3.0.01,5]dodecane-11-carboxylate (33):**

Product **33** was obtained from keto-ester **32** with L-valinol following the general protocol 2. The crude was purified by flash column chromatography on silica gel (cyclohexane/ethyl acetate: 100/0 to 0/100) to give product **33** (yield = 78%, colorless oil), as a single diastereoisomer. ^1^H NMR (300 MHz, CDCl_3_): δ 4.14–4.00 (m, 2H), 3.76–3.57 (m, 2H), 3.54–3.31 (m, 2H), 2.59–2.41 (m, 1H), 2.39–1.92 (m, 4H), 1.86–1.57 (m, 2H), 1.40 (s, 9H), 0.92 (d, *J* = 6.8 Hz, 3H), 0.88 (d, *J* = 6.8 Hz, 3H) ppm. ^13^C (75 MHz, CDCl_3_): δ 170.4, 154.2, 98.9, 80.0, 66.9, 60.7, 59.2, 43.9, 33.6, 33.0, 29.8, 28.5, 27.2, 25.2, 20.1, 18.6 ppm. HRMS (ESI, *m*/*z*): [M+H]^+^ calculated for C_17_H_29_N_2_O_4_, 325.2127; found 325.2139.

***Tert*** **-butyl (*1R,4S,9R*)-4-isopropyl-6-oxo-2-oxa-5,11-diazatricycl [7.5.0.01,5]tetradecane-11-carboxylate (35):**

Product **35** was obtained from keto-ester **34** and L-valinol using general protocol 2. The crude product was purified by flash column chromatography on silica gel (cyclohexane/ethyl acetate: 100/0 to 0/100) to afford compound **35** (yield = 89%, colorless oil), as a mixture of two diastereoisomers (d.r. = 90:10). Data for the major compound **35**: ^1^H NMR (300 MHz, CDCl_3_): δ 4.14–3.86 (m, 2H), 3.84–3.53 (m, 2H), 3.44–3.30 (m, 3H), 2.61–2.26 (m, 2H), 2.00–1.53 (m, 8H), 1.44 (s, 9H), 0.94 (d, *J* = 6.9 Hz, 3H), 0.90 (d, *J* = 6.8 Hz, 3H) ppm. ^13^C (75 MHz, CDCl_3_): δ 170.5, 156.5, 96.0, 79.8, 66.8, 61.6, 48.0, 47.7, 44.4, 42.9, 33.2, 32.6, 30.6, 28.6, 20.9, 20.4, 20.1, 19.3 ppm. HRMS (ESI, *m*/*z*): [M+H]^+^ calculated for C_19_H_33_N_2_O_4_, 353.2440; found 353.2459.

***Tert*** **-butyl (*1R,4S,8R*)-4-isopropyl-6-oxo-2-oxa-5,10-diazatricyclo[6.4.0.01,5]dodecane-10-carboxylate (37):**

Compound **37** was obtained from keto-ester **36** and L-valinol using general protocol 2. The crude was purified by flash column chromatography on silica gel (cyclohexane/ethyl acetate: 100/0 to 0/100) to give product **37** (yield = 90%, colorless oil), as a single diastereoisomer. ^1^H NMR (300 MHz, CDCl_3_): δ 4.21 (dd, *J* = 8.8 Hz, *J* = 7.6 Hz, 1H), 3.83 (dd, *J* = 8.8 Hz, 6.4 Hz, 1H), 3.70–3.35 (m, 5H), 2.63–2.47 (m, 3H), 2.10–1.88 (m, 2H), 1.68–1.53 (m, 1H), 1.46 (s, 9H), 1.03 (d, *J* = 6.7 Hz, 3H), 0.87 (d, *J* = 6.7 Hz, 3H) ppm. ^13^C (75 MHz, CDCl_3_): δ 178.2, 155.6, 99.2, 80.2, 71.2, 61.8, 42.7, 41.5, 40.6, 39.7, 36.8, 33.9, 30.6, 28.7, 20.7, 19.1 ppm. HRMS (ESI, *m*/*z*): [M+H]^+^ calculated for C_17_H_29_N_2_O_4_, 325.2127; found 325.2144.

***Tert*** **-butyl *N*-[(*3S,7aR,9S,11aR*)-3-isopropyl-5-oxo-3,6,7,7a,8,9,10,11-octahydro-2H-oxazolo[2,3-j]quinolin-9-yl]carbamate (39) and *Tert*-butyl *N*-[(*3S,7aR,9R,11aR*)-3-isopropyl-5-oxo-3,6,7,7a,8,9,10,11-octahydro-2H-oxazolo[2,3-j]quinolin-9-yl]carbamate (40):**

Products **39** and **40** were obtained from keto-ester **38** and L-valinol using general protocol 2. The crude product was purified by flash column chromatography on silica gel (cyclohexane/ethyl acetate: 100/0 to 0/100) to afford compounds **39** (yield = 41%, white solid) and **40** (Yield= 29%, white powder). Compound **39** was crystallized from dichloromethane using the slow evaporation technique at room temperature under atmospheric pressure.

Data for **39**: m.p. = 141–143 °C. ^1^H NMR (300 MHz, CDCl_3_): δ 4.56–4.28 (m, 1H), 4.17–3.96 (m, 2H), 3.78–3.61 (m, 2H), 2.67–2.53 (m, 1H), 2.48–2.28 (m, 1H), 2.11–1.85 (m,5H), 1.84–1.68 (m, 4H), 1.43 (s, 9H), 0.94 (d, *J* = 6.9 Hz, 3H), 0.90 (d, *J* = 6.9 Hz, 3H) ppm. ^13^C (75 MHz, CDCl_3_): δ 170.2, 155.2, 93.3, 66.3, 61.4, 44.1, 40.0, 34.7, 32.5, 30.9, 30.2, 29.6, 28.4, 26.9, 22.4, 19.9, 18.7 ppm. HRMS (ESI, *m*/*z*): [M+H]^+^ calculated for C_19_H_33_N_2_O_4_, 353.2440; found 353.2446.

Data for **40**: ^1^H NMR (300 MHz, CDCl_3_): 4.35 (d, *J* = 7.4 Hz, 1H), 4.05–3.96 (m, 1H), 3.95–3.89 (m, 2H), 3.84–3.64 (m, 1H), 3.00 (d sept, *J* = 7.0 Hz, *J* = 3.2 Hz, 1H), 2.51–2.39 (m, 2H), 2.07–1.97 (m, 5H), 1.95–1.83 (m, 4H), 1.45 (s, 9H), 0.87 (d, *J* = 7.1 Hz, 3H), 0.72 (d, *J* = 7.0 Hz, 3H) ppm. ^13^C (75 MHz, CDCl_3_): δ 68.2, 155.2, 92.9, 62.6, 60.6, 43.9, 38.0, 30.2, 29.5, 28.43, 28.40, 26.9, 24.6, 22.4, 18.9, 19.5, 18.7 ppm. LCMS (ESI, *m*/*z*): [M+H]^+^ = 353.

#### 3.2.3. General Protocol 3: Functionalization of Lactam Ring by Alkylation Reaction

**Scheme 9:**
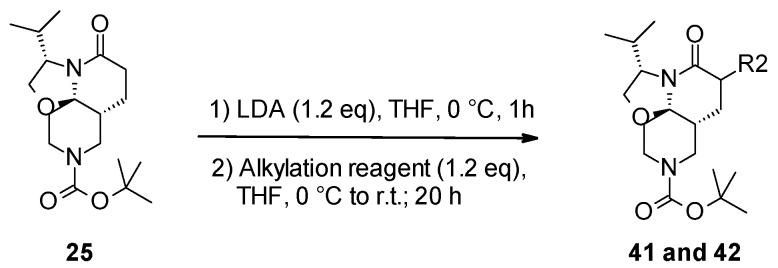
Functionalization of Lactam Ring by Alkylation Reaction.

*N*-Boc lactam **25** (1 eq) was dissolved in dry THF (0.15 M). The solution was cooled down to 0 °C, and then LDA (1.2–3 eq) was added dropwise. The mixture was stirred for 1 h, then a solution of alkylating reagent (1.2–2 eq) in dry THF was added dropwise. The resulting mixture was warmed up to room temperature and stirred for 3–20 h. The solution was quenched with H_2_O and extracted with diethyl ether. The organic layer was washed with a saturated aqueous solution of NH_4_Cl, dried over MgSO_4_, filtered, and concentrated under vacuum. The crude product obtained was purified by flash column chromatography over silica gel (cyclohexane/ethyl acetate: 100/0 to 0/100) to afford the corresponding desired product, (see Scheme 9).

***Tert*** **-butyl (*3S,7aR,11aR*)-3-isopropyl-6-methyl-5-oxo-2,3,6,7,7a,8,10,11-octahydrooxazolo[2,3-j][1,6]naphthyridine-9-carboxylate (41):**

Product **41** was obtained following general protocol 3, using iodomethane as an alkylating reagent (Yield = 75%, colorless oil) as two diastereoisomers separable by flash chromatography.

Diastereoisomer 1: ^1^H NMR (300 MHz, CDCl_3_): δ 4.17–3.97 (m, 4H); 3.71 (d, *J* = 8.1 Hz, 6.0 Hz, 1H); 3.29–3.11 (m, 1H); 2.94–2.77 (m, 1H); 2.53–2.38 (m, 1H); 2.01–1.50 (m, 6H), 1.45 (s, 9H); 1.26 (d, *J* = 7.6 Hz, 3H), 0.91 (d, *J* = 6.9 Hz, 3H), 0.88 (d, *J* = 6.9 Hz, 3H) ppm. ^13^C NMR (75 MHz, CDCl_3_): δ 173.5, 155.1, 92.5, 79.9, 66.9, 61.2, 45.7, 41.4, 40.4, 37.1, 32.9, 31.7, 30.4, 28.5, 19.9, 19.8, 18.8 ppm; [ES+ MS] *m*/*z* 353 (MH^+^). HRMS (ESI,*m*/*z*): [M+H]^+^ calculated for C_19_H_33_N_2_O_4_, 353.2440; found 353.2441.

Diastereoisomer 2: ^1^H NMR (300 MHz, CDCl_3_): δ 4.13–4.01 (m, 2H), 3.92 (d, *J* = 8.8 Hz, 7.1 Hz, 2H), 3.78 (d, *J* = 8.8 Hz, 5.5 Hz, 1H), 3.27–3.09 (m, 1H), 2.96–2.77 (m, 1H), 2.58–2.46 (m, 1H), 2.08–1.56 (m, 6H), 1.44 (s, 9H), 1.21 (d, *J* = 7.2 Hz, 3H), 0.89 (d, *J* = 6.6 Hz, 3H), 0.87 (d, *J* = 6.6 Hz, 3H) ppm. ^13^C NMR (75 MHz, CDCl_3_): δ 173.1, 155.2, 92.6, 79.9, 66.3, 61.5, 44.8, 41.2, 40.4, 37.8, 33.5, 31.9, 31.9, 28.5, 19.7, 18.7, 18.2 ppm. LCMS (ESI, *m*/*z*): [M+H]^+^ = 353.

***Tert*** **-butyl (*3S,7aR,11aR*)-6-fluoro-3-isopropyl-5-oxo-2,3,6,7,7a,8,10,11-octahydrooxazolo[2,3-j][1,6]naphthyridine-9-carboxylate (42):**

Product **42** was obtained following general protocol 3, using *N*-(benzenesulfonyl)-*N*-fluoro-benzenesulfonamide as an alkylating reagent. Yield =16%; colorless oil; single diastereoismer. ^1^H NMR (300 MHz, CDCl_3_): δ 4.93 (ddd, *J* = 48.0 Hz, *J* = 6.6 Hz, *J* = 2.7 Hz, 1H), 4.22–4.13 (m, 1H), 4.05 (dd, *J* = 8.7 Hz, *J* = 7.4 Hz, 1H), 4.04–3.88 (m, 2H), 3.82 (dd, *J* = 8.7 Hz, *J* = 6.1 Hz, 1H), 3.30–3.10 (m, 1H), 2.96–2.73 (m, 1H), 2.34–1.82 (m, 4H), 1.81–1.62 (m, 2H), 1.45 (s, 9H), 0.93 (d, *J* = 6.9 Hz, 3H), 0.90 (d, *J* = 6.9 Hz, 3H) ppm. ^13^C NMR (75 MHz, CDCl_3_): δ 165.9 (d, *J* = 22.3 Hz), 154.9, 92.8, 85.5, 83.2, 80.2, 66.8, 61.4, 44.9, 41.1, 36.8, 32.1, 31.5, 28.6 (d, *J* = 22.9 Hz), 28.5, 19.8, 18.7 ppm. LCMS (ESI, *m*/*z*): [M+H]^+^ = 357.

#### 3.2.4. Deprotection of Lactams

**(*3S,7aR,11aR*)-3-isopropyl-3,6,7,7a,8,9,10,11-octahydro-2H-oxazolo[2,3-j][1,6]naphthyridin-5-one;hydrochloride (43):**

*N*-Boc lactam **25** (3.2 g, 9.46 mmol, 1 eq) was dissolved in 1,4-dioxane (100 mL), and then a solution of HCl (4N in dioxane) (24 mL, 94.6 mmol, 10 eq) was added. The mixture was stirred at room temperature overnight. The solvent was removed under vacuum to afford the product **43** with a quantitative yield as a white powder. ^1^H NMR (300 MHz, CD_3_OD): δ 4.18–4.04 (m, 2H), 3.99–3.87 (m, 1H), 3.48–3.28 (m, 4H), 3.24–3.09 (m, 1H), 2.70 (dd, *J* = 18.6 Hz, *J* = 6.3 Hz, 1H), 2.26–1.84 (m, 6H), 0.95 (d, *J* = 6.9 Hz, 3H), 0.94 (d, *J* = 6.9 Hz, 3H) ppm. ^13^C NMR (75 MHz, CD_3_OD): δ 172.3, 91.4, 67.8, 63.3, 46.1, 42.6, 38.5, 33.5, 30.9, 29.3, 21.4, 20.1, 19.0 ppm. HRMS (ESI, *m*/*z*): [M+H]^+^ calcd. for C_13_H_22_N_2_O_2_, 239.1760; found 239.1743.

**(3S,7aR,11aR)-3-isopropyl-3,6,7,7a,8,9,10,11-octahydro-2H-oxazolo[2,3-j][1,6]naphthyridin-5-one;formic acid (44):**

Lactam **12** (655 mg, 1.99 mmol, 1 eq.) was dissolved in methanol (20 mL), then were added Pd/C 10% (127 mg, 1.20 mmol, 0.12 mmol, 10 mol%) and ammonium formate (629 mg, 9.97 mmol, 5 eq). The mixture was refluxed for 30 min. The solution was filtered over celite, then the filtrate was concentrated under reduced pressure to afford compound **44** with a quantitative yield as a white powder. ^1^H NMR (300 MHz, CD_2_Cl_2_): δ 8.44 (brs, 1H), 4.13–3.99 (m, 2H), 3.77 (dd, *J* = 8.3 Hz, *J* = 5.6 Hz, 1H), 3.35–3.11 (m, 3H), 2.99 (td, *J* = 13.1 Hz, *J* = 3.2 Hz, 1H), 2.68–2.55 (m, 1H), 2.48–2.20 (m, 2H), 2.10 (td, *J* = 14.4 Hz, *J* = 4.6 Hz, 1H), 2.01–1.70 (m, 4H), 0.92 (d, *J* = 6.4 Hz, 3H), 0.90 (d, *J* = 6.4 Hz, 3H) ppm. HRMS (ESI, *m*/*z*): [M+H]^+^ calcd. for C_13_H_22_N_2_O_2_, 239.1760; found 239.1759.

#### 3.2.5. R3 Functionalization of Lactams **43** and **44**

**(3S,7aR,11aR)-9-(cyclohexylmethyl)-3-isopropyl-2,3,6,7,7a,8,10,11-octahydrooxazolo[2,3-j][1,6]naphthyridin-5-one (45):**

To a solution of lactam **44** (100 mg, 0.352 mmol) in MeCN (2 mL), bromomethylcyclohexane (126 mg, 0.528 mmol), and K_2_CO_3_ (146 mg, 1.06 mmol) were added. After 2 h of stirring at room temperature, NaI (39.5 mg, 0.264 mmol) was added. The mixture was then heated to 80 °C and stirred for 60 h. The solvent was removed under vacuum, and the mixture was washed with dichloromethane and a saturated solution of Na_2_CO_3_. The organic layer was dried over magnesium sulfate, and the solvent was removed under vacuum. The crude product was then purified by flash column chromatography over silica gel (cyclohexane/ethyl acetate: 100/0 to 0/100) to afford the corresponding desired product **45** (78.4 mg, 67%) as a colorless oil. ^1^H NMR (300 MHz, CD_2_Cl_2_): δ 4.09–3.96 (m, 2H), 3.78 (dd, *J* = 8.0 Hz, *J* = 5.7 Hz, 1H), 3.98 (d, *J* = 7.5 Hz, 1H), 3.79 (d, *J* = 5.7 Hz, 1H), 3.76 (d, *J* = 6.2 Hz, 1H), 2.75–2.46 (m, 3H), 2.44–2.26 (m, 3H), 2.18–1.87 (m, 5H), 1.85–1.57 (m, 8H), 1.53–1.39 (m, 1H), 1.37–1.16 (m, 3H), 0.89 (d, *J* = 7.9 Hz, 3H), 0.92 (d, *J* = 7.9 Hz, 3H), 0.90–0.82 (m, 2H) ppm. ^13^C NMR (75 MHz, CDCl_3_): δ 170.3, 92.8, 66.1, 65.0, 61.2, 55.3, 51.3, 40.4, 35.4, 32.4, 32.3, 30.8, 26.9, 26.2, 22.1, 19.9, 18.7 ppm. HRMS (ESI, *m*/*z*): [M+H]^+^ calcd. for C_20_H_35_N_2_O_2_, 335.2699; found 335.2693.

**(*3S,7aR,11aR*)-3-isopropyl-9-[[4-(1-piperidyl)phenyl]methyl]-2,3,6,7,7a,8,10,11-octahydrooxazolo[2,3-j][1,6]naphthyridin-5-one (46):**

In a tube charged with 4-(1-piperidyl)benzaldehyde (103 mg, 0.5 mmol, 3 eq), lactam **43** (50 mg, 0.18 mmol, 1 eq), DIEA (31 µL, 0.18 mmol, 1 eq) dissolved in DCE (1 mL), and NaBH(OAc)_3_ (116 mg, 0.5 mmol, 3 eq) were added. The mixture was stirred at room temperature overnight. The crude was evaporated with celite and then purified by flash column chromatography over silica gel (cyclohexane/ethyl acetate: 100/0 to 0/100) to afford the desired product **46** (58 mg, 77%) as a colorless oil. ^1^H NMR (300 MHz, CDCl_3_): δ 7.18 (d, *J* = 8.7 Hz, 2H), 6.90 (d, *J* = 8.7 Hz, 2H), 4.11 (m, 1H), 3.99 (dd, *J* = 8.7 Hz, *J* = 7.6 Hz, 1H), 3.78 (dd, *J* = 8.7 Hz, *J* = 6.3 Hz, 1H), 3.50 (d, *J* = 13.0 Hz, 1H), 3.34 (d, *J* = 13.0 Hz, 1H), 3.18–3.12 (m, 4H), 2.82 -2.66 (m, 2H), 2.64–2.52 (m, 1H), 2.49–2.12 (m, 5H), 2.10–1.88 (m, 2H), 1.85–1.53 (m, 9H) 0.96 (d, *J* = 6.9 Hz, 3H), 0.92 (d, *J* = 6.9 Hz, 3H) ppm. ^13^C NMR (75 MHz, CDCl_3_): δ 170.2, 151.3, 129.5, 116.2, 92.7, 66.1, 61.98, 61.2, 54.5, 50.8, 50.4, 40.3, 32.3, 30.8, 25.9, 24.3, 21.8, 18.8, 18.6 ppm. HRMS (ESI, *m*/*z*): [M+H]^+^ calcd. For C_25_H_38_N_3_O_2_, 412.2964; found 412.2961.

**(*3S,7aR,11aR*)-3-isopropyl-9-[3-[4-(trifluoromethyl)phenyl]propanoyl]-2,3,6,7,7a,8,10,11-octahydrooxazolo[2,3-j][1,6]naphthyridin-5-one (47):**

COMU (234 mg, 0.546 mmol, 1.5 eq) was dissolved in ethyl acetate (1 mL), and then 3-[4(trifluoromethyl)phenyl]propanoic acid (79.4 mg, 0.364 mmol, 1 eq), and DIEA (94 µL, 0.5 mmol, 1.5 eq) were added. The mixture was stirred at room temperature, then a solution of lactam **43** (100 mg, 0.364 mmol, 1 eq) and DIEA (94 µL, 0.5 mmol, 1.5 eq) were added. The resulting solution was stirred at room temperature for 4 h. The mixture was washed with an aqueous solution of NaHCO_3_ and brine. The layers were separated, then the organic layer was dried over MgSO_4_, filtered, and concentrated under reduced pressure to give the crude product. The crude was purified by reverse phase chromatography using H_2_O/MeCN (90/10 to 0/100) to afford product **47** (102 mg, 64%) as a colorless oil. ^1^H NMR (300 MHz, CDCl_3_): δ 7.54 (d, *J* = 8.1 Hz, 2H), 7.36–7.30 (m, 2H), 4.68–4.54 (m, 1H), 4.20–4.06 (m, 1H), 3.87–3.75 (m, 1H), 3.75–3.62 (m, 1H), 3.62–3.48 (m, 0.5H), 3.24–3.16 (m, 0.5H), 3.11–2.98 (m, 2H), 2.80–2.57 (m, 2H), 2.57–2.35 (m, 2H), 2.10–1.90 (m, 1H), 1.88–1.51 (m, 7H), 0.96–0.83 (m, 6H) ppm. ^13^C NMR (75 MHz, CDCl_3_): δ 170.5, 170.4, 169.7, 169.3, 145.5, 128.9, 128.6 (q, *J* = 32 Hz), 125.7 (q, *J* = 3.7 Hz), 124.5 (q, *J* = 272.3 Hz), 92.1, 66.5, 61.6, 61.4, 46.7, 42.9, 42.6, 40.0, 39.7, 38.8, 34.3, 32.5, 32.1, 32.0, 31.5, 31.1, 31.0, 29.9, 29.8, 20.5, 19.7, 19.6, 18.7, 18.6 ppm. HRMS (ESI, *m*/*z*): [M+H]^+^ calcd. for C_23_H_30_N_2_O_3_F_3_, 439.2209; found 439.2182.

**(*3S,7aR,11aR*)-3-isopropyl-9-[2-[4-(trifluoromethyl)phenyl]ethylsulfonyl]-2,3,6,7,7a,8,10,11-octahydrooxazolo[2,3-j][1,6]naphthyridin-5-one (48):**

In a tube charged with lactam **43** (50 mg, 0.18 mmol, 1 eq) dissolved in 1 mL of anhydrous MeCN, DIEA (62 µL, 0.36 mmol, 2 eq) was added dropwise. The mixture was stirred until complete solubilization of **43** was achieved, then 2-[4-(trifluoromethyl)phenyl]ethanesulfonyl chloride (50 mg, 0.18 mmol, 1 eq) was added. The mixture was stirred at room temperature overnight. The crude was evaporated with celite and then purified by flash chromatography over silica gel (cyclohexane/ethyl acetate: 100/0 to 50/50) to afford the desired product **48** (49 mg, 57%) as a colorless oil. ^1^H NMR (300 MHz, CDCl_3_): δ 7.60 (d, *J* = 8.1 Hz, 2H), 7.35 (d, *J* = 8.1 Hz, 2H), 4.19–4.09 (m, 1H), 3.98 (dd, *J* = 8.9 Hz, *J* = 7.4 Hz, 1H), 3.83–3.65 (m, 3H), 3.28 (dd, *J* = 12.5 Hz, *J* = 3.0 Hz, 1H), 3.18 (br s, 4H), 2.97 (dt, *J* = 12.5 Hz, *J* = 3.0 Hz, 1H), 2.63 (ddd, *J* = 18.3 Hz, *J* = 8.8 Hz, *J* = 2.5 Hz, 1H), 2.25–2.09 (m, 1H), 2.08–1.95 (m, 2H), 1.90–1.66 (m, 4H), 0.96 (d, *J* = 6.9 Hz, 3H), 0.93 (d, *J* = 6.8 Hz, 3H). ^13^C NMR (75 MHz, CDCl_3_): δ 169.6, 142.0, 129.5 (q, *J* = 32 Hz), 128.8, 125.8 (q, *J* = 3.8 Hz), 124.0 (q, *J* = 273 Hz), 91.4, 66.4, 61.5, 50.6, 47.1, 43.1, 39.4, 32.1, 32.0, 29.7, 29.2, 20.3, 19.6, 18.6 ppm. HRMS (ESI, *m*/*z*): [M+H]^+^ calcd. for C_22_H_30_N_2_O_4_F_3_S, 475.1878; found 475.1875.

### 3.3. X-ray Structural Determination

A suitable single crystal of compound **39** was selected, glued at the tip of a Mitegen sample holder, and mounted on a Bruker APEX DUO diffractometer. The crystal was kept at RT during data collection. Using the Olex2 program [44], the structure was solved with SHELXT [45] and refined by least-squares procedures on F^2^ with SHELXL [46]. Crystal Data for C_19_H_32_N_2_O_4_ (M = 352.47 g/mol): orthorhombic, space group P2_1_2_1_2_1_ (no. 19), a = 9.2668(12) Å, b = 12.1741(17) Å, c = 17.512(3) Å, V = 1975.6(5) Å^3^, Z = 4, T = 296 K, μ(CuKα) = 0.67 mm^−1^, Dcalc = 1.185 g/cm3, 60262 reflections measured (8.8° ≤ 2Θ ≤ 138.4°), 3662 unique (Rint = 0.0510, Rsigma = 0.0182), which were used in all calculations. The final R1 was 0.0339 (I > 2σ(I)) and wR2 was 0.0953 (all data). CCDC 2215399 contains the supplementary crystallographic data for this paper. These data can be obtained free of charge from the Cambridge Crystallographic Data Centre via www.ccdc.cam.ac.uk/structures, accessed on 20 December 2022.

### 3.4. Determination of Compound ***44*** Solubility

To determine compound solubility, 10 mM of the compound **44** in DMSO was diluted 50-fold either in PBS at pH 7.4 or in organic MeOH solvent in PP tubes (n = 3 for PBS and methanol). The tubes were gently shaken for 24 h at room temperature. Then, the three PBS tubes and three of the six methanol tubes were centrifuged for 5 min at 4000 rpm and filtered over 0.45 μm filters (Millex-LH Millipore). The sample was diluted 50-fold in MeOH before LC-MS/MS analysis. The test was performed in triplicate. The solubility was determined by the following ratio: (AUC_PBS_/AUC_MeOH_) × 200.

To determine compound LogD, 10 mM of the compound **44** in DMSO was diluted 50-fold in a mixture of 1:1 octanol:PBS at pH 7.4. The mixture was gently shaken for 2 h at room temperature. Each sample was then diluted 50-fold in MeOH before LC-MS/MS analysis. For each compound, the test was performed in triplicate. LogD was determined as the logarithm of the ratio of product concentrations in octanol and PBS, respectively, determined by mass signals.

## 4. Conclusions

Here, we report the rapid and efficient synthesis of novel natural-inspired tricyclic spirolactams from keto-esters by stereoselective Meyers’ lactamization. The synthetic route was straightforward, reliable, and versatile, and it generated new tricyclic spirolactams with a large scaffold diversity. This novel family of molecules contains a spiranic carbon, providing rigidity and three-dimensionality as well as three easily functionalized positions to populate new biologically relevant chemical spaces. On average, these molecules exhibit good drug-like properties: molecular weight < 500, clogP < 5, and a high fraction of sp^3^-hybridized carbon atoms (Fsp^3^ > 0.6). A focused library of new three-dimensional tricyclic spirolactams is currently produced and screened on a variety of biological targets. Among them, several compounds have already been identified as lead compounds against mycobacteria [47]. The structure-activity relationship studies will be reported soon.

## 5. Patents

B.D., B.V., L.F., M.F., N.W. and S.T. are inventors on a patent application covering the TriSLa described in this manuscript. The remaining authors declare no competing interest.

## Figures and Tables

**Table 1:** Optimization of Meyers’ lactamization between 4-piperidone-derived keto-acid **8** or keto-ester **9** and R-phenylglycinol.

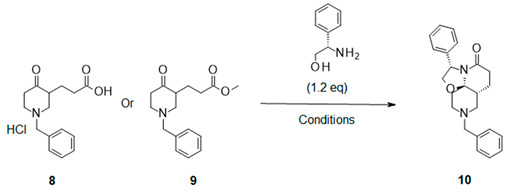			
Entry	Substrate ^a^	Conditions	Conversion ^b^
1	**8**	Toluene, reflux, 20 h	66%
2	**8**	Toluene, 150 °C (µW), 2 h	76%
3	**9**	Pivalic acid (1.2 eq), Toluene, 150 °C (µW), 1 h	100%

^a^ Keto acid **8** and Keto-ester **9** were used as racemic forms. ^b^ Conversion was determined by LCMS at 215 nm.

**Table 2:** Meyers’ lactamization of benzyl–δ-keto-ester **9** with amino-alcohols.

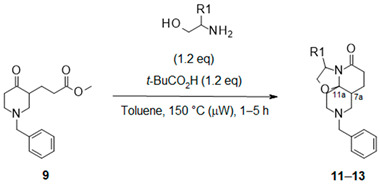				
Entry	Amino-Alcohol	Product	Isolated Compound (Yield) ^a^	d.r. ^b^
1	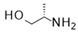	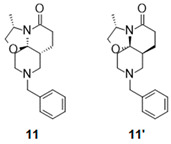	**11 + 11′** (46%)	70:30
2	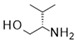	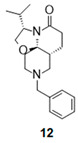	**12** (75%)	100:0
3	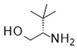	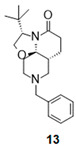	**13** (40%)	100:0

^a^ Isolated yield after purification. ^b^ Ratio of diastereoisomers (before separation) measured by LCMS at 215 nm.

**Table 3:** Meyers’ lactamization of Boc-protected δ-keto-ester **16** with various 3-aminopropanols.

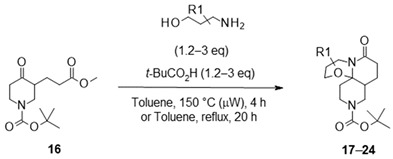				
Entry	Amino-Alcohol	Product	Isolated Compound (Yield) ^d^	d.r. ^e^
1	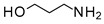	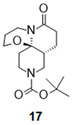	**17** ^a^ (79%)	
2	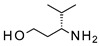	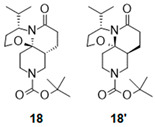	**18** ^#^ (18%)	90:10
3	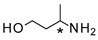	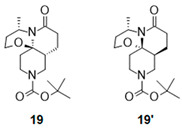	**19 + 19′** ^b^ (86%)	50:50
4	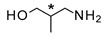	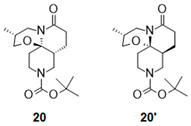	**20** ^c^ (63%)	60:40
5	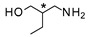	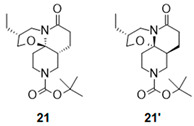	**21 + 21′** ^b^ (89%)	70:30
6	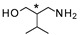	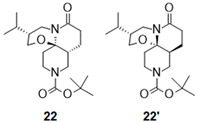	**22** ^c^ (92%)	96:4
7	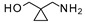	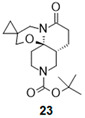	**23** ^a^ (88%)	
8	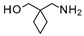	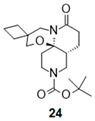	**24** ^a^ (98%)	

* The amino alcohol was used as a racemic mixture. ^#^ All compounds were synthesized in toluene under microwave (µW) irradiation apart from compound **18**, which was synthesized in toluene under refluxing conditions. ^a^ Product was obtained as a racemic mixture from non-chiral amino alcohol. ^b^ Both diastereoisomers were obtained as racemic mixtures from racemic amino-alcohol. ^c^ Product was obtained as a single diastereoisomer in a racemic mixture from racemic amino-alcohol. ^d^ Isolated yield after purification. ^e^ Ratio of diastereoisomers (before separation) measured by LCMS at 215 nm.

**Table 4:** Extension of Meyers’ lactamization reaction to various Boc-protected keto-esters and amino-alcohols.

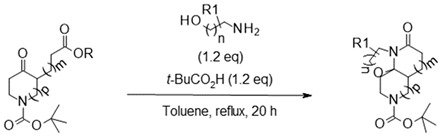					
Entry	Boc-Keto-Ester ^a^	Amino-Alcohol	Product	Isolated Compound (Yield) ^c^	d.r. ^d^
1	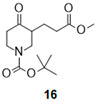	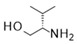	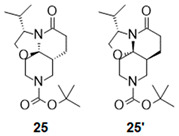	**25** (84%) * **25** (88%)	90:10
2	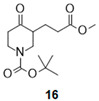	** 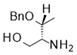 **	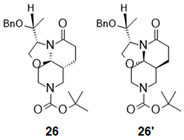	**26** (50%)	94:6
3	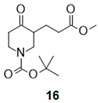	** 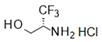 **	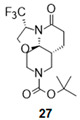	**27** (59%)	100:0
4	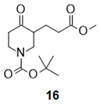	** 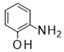 **	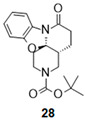	**28** ^b^ (98%)	
5	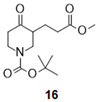	** 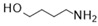 **	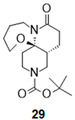	**29** ^b^ (81%)	
6	** 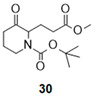 **	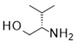	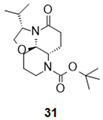	**31** (49%)	100:0
7	** 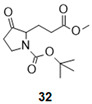 **	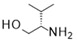	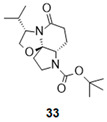	**33** (78%)	100:0
8	** 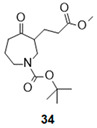 **	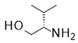	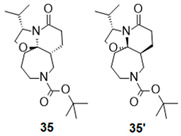	**35** (89%)	90:10
9	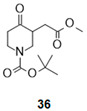	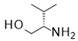	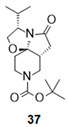	**37** (90%)	100:0

* 84% yield was obtained for compound **25** using previously optimized conditions (toluene, 150 °C (µW), 2 h). ^a^ Keto-ester was used as a racemic mixture. ^b^ Product was obtained as a racemic mixture from non-chiral amino-alcohol. ^c^ Isolated yield after purification. ^d^ Ratio of diastereoisomers (before separation) measured by LCMS at 215 nm.

## Data Availability

Data is contained within the article and Supplementary Materials.

## Supplementary Materials

The following supporting information can be downloaded at: https://www.mdpi.com/article/10.3390/ph16030413/s1, NMR spectra of compounds **10**–**48**.
