# Supplementary material for: Rapid and Efficient Access to Novel Bio-Inspired 3-Dimensional Tricyclic SpiroLactams as Privileged Structures via Meyers’ Lactamization
Source: Pharmaceuticals (Basel). 2023 Mar 8;16(3):413. doi: 10.3390/ph16030413 (PMC10054226; doi:10.3390/ph16030413)

Supplementary Information

Rapid and efficient access to novel bio-inspired 3-dimensional Tricyclic SpiroLactams as privileged structures via Meyers' lactamization

Salia Tangara [1], Léo Faïon [1], Catherine Piveteau [1], Frédéric Capet [2], Romain Godelier [1], Marion Michel [1], Marion Flipo [1], Benoit Deprez [1], Nicolas Willand [1], Baptiste Villemagne [1, *]

[1] Univ. Lille, Inserm, Institut Pasteur de Lille, U1177 - Drugs and Molecules for Living Systems, F-59000, Lille, France.

[2] Univ. Lille, CNRS, Centrale Lille, ENSCL, Univ. Artois, UMR 8181 - UCCS - Unité de Catalyse et Chimie du Solide, F-59000 Lille, France.

*****Correspondence: baptiste.villemagne@univ-lille.fr;

^1H and ^{13}C NMR Spectra:

16, ^1H NMR (300 MHz, $CDCl_3$)

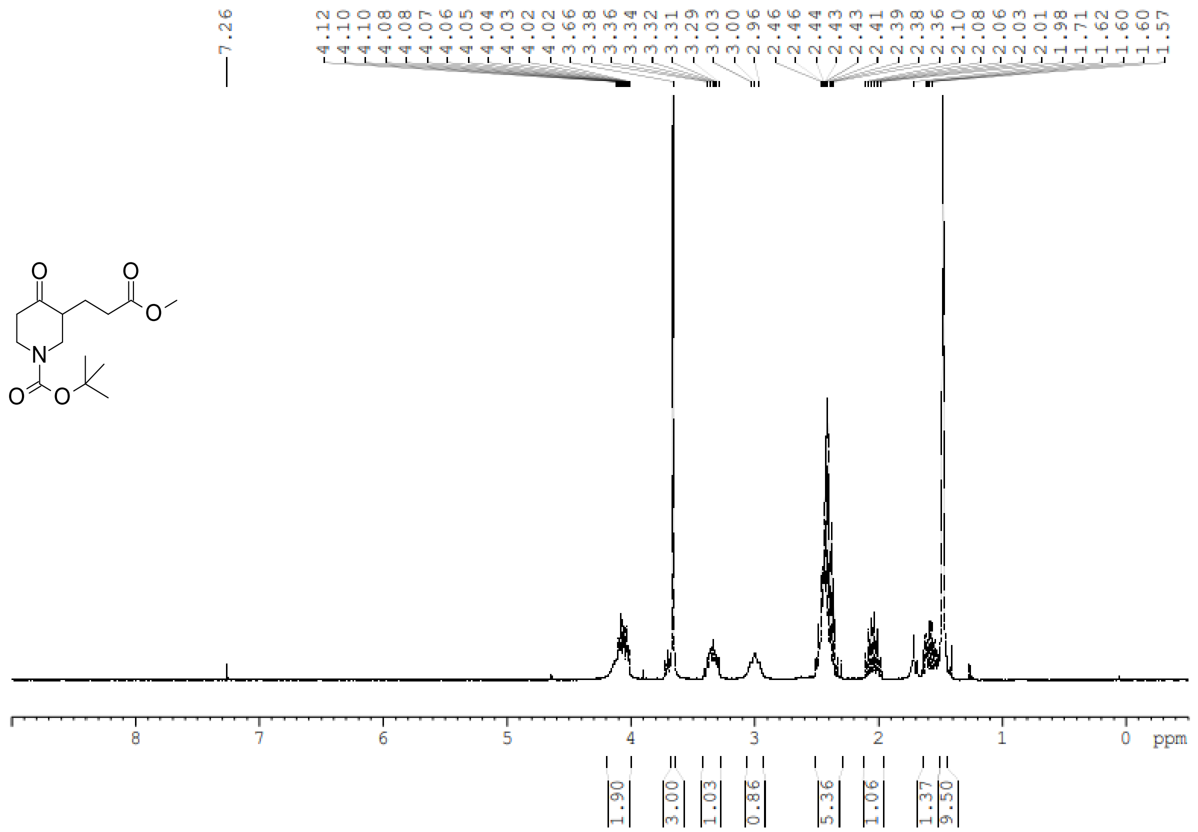

16, ^{13}C NMR (75 MHz, $CDCl_3$)

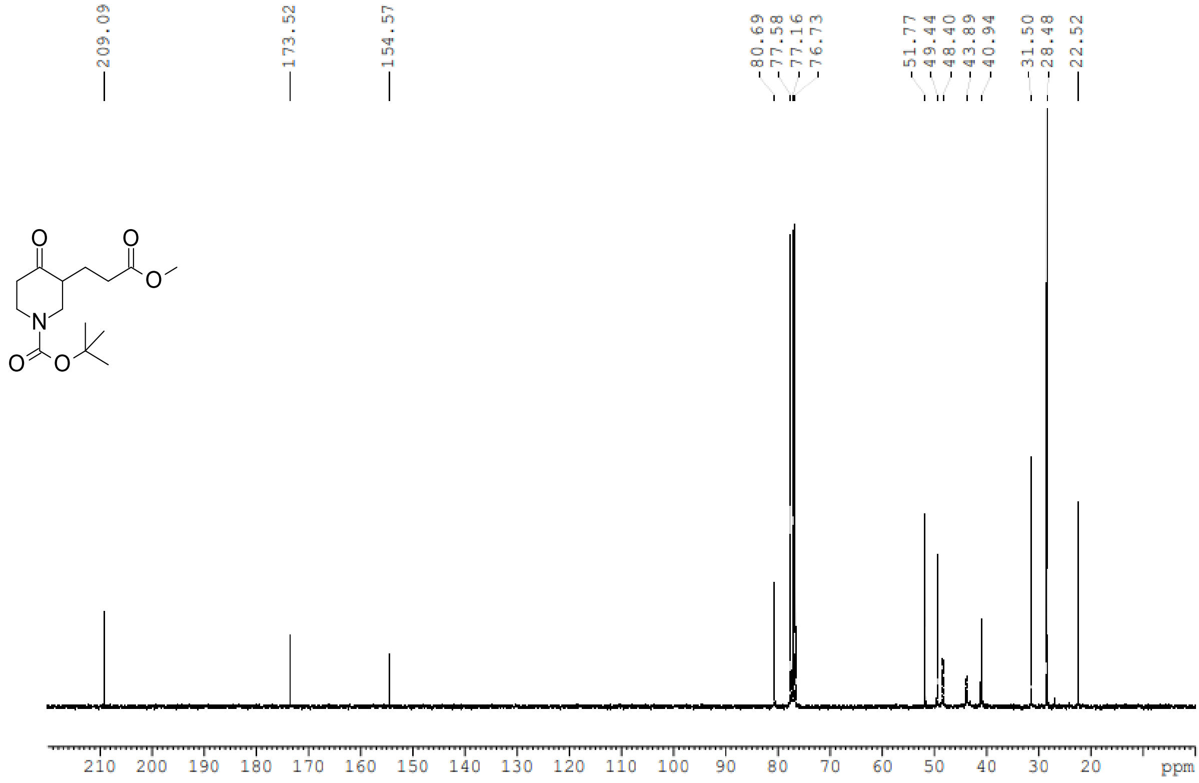

30, ^{1}H NMR (300 MHz, $CDCl_3$)

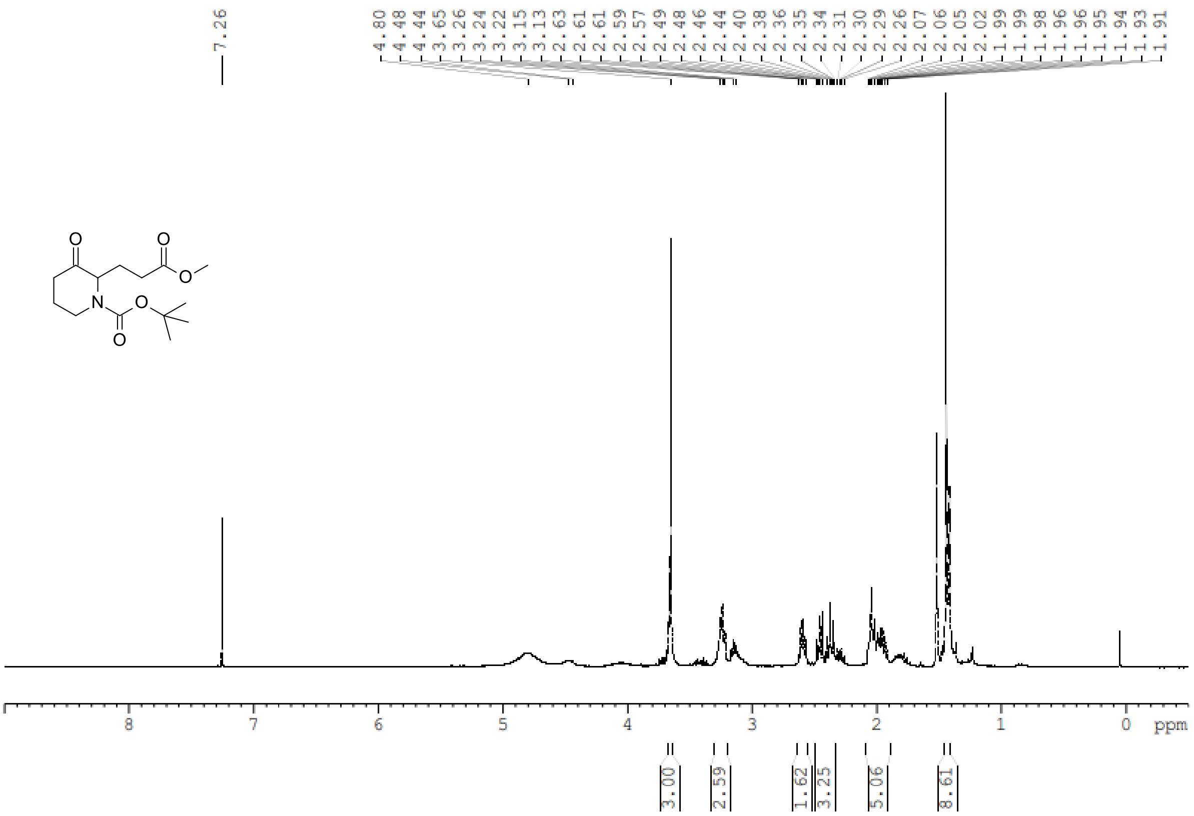

32, ^{1}H NMR (300 MHz, $CDCl_3$)

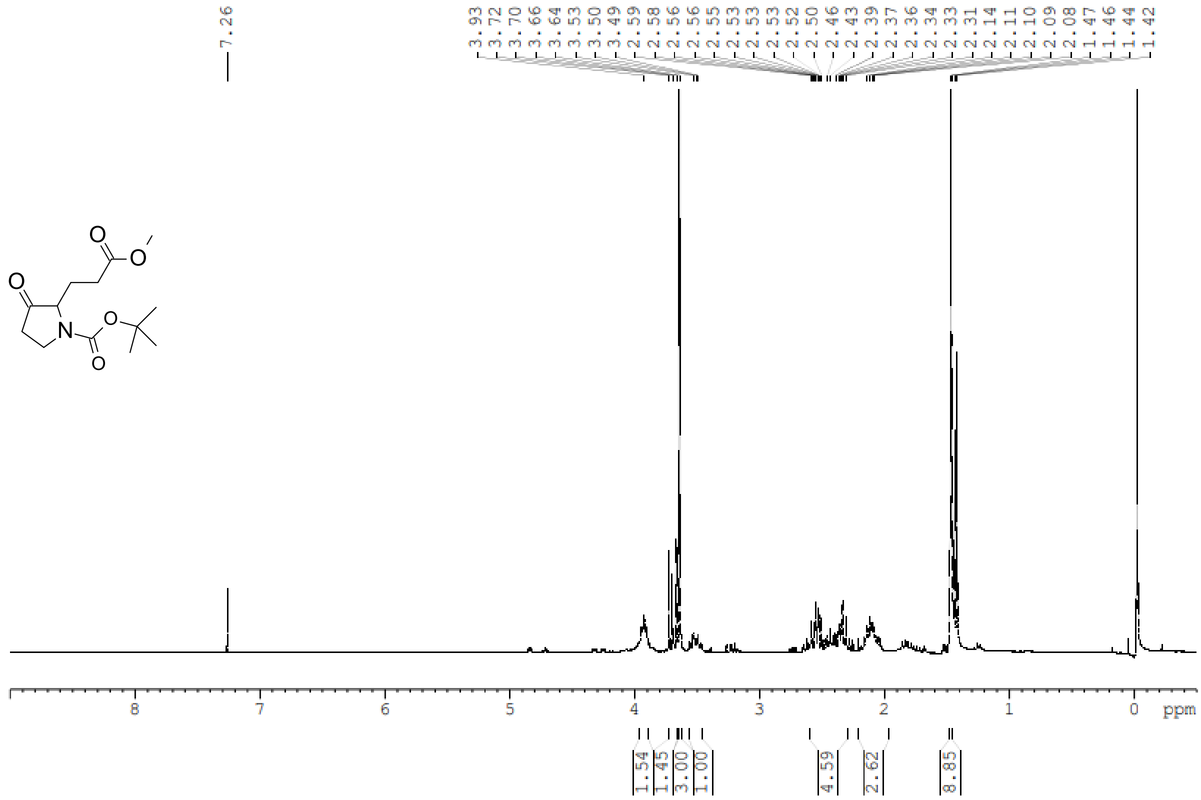

34, ^{1}H NMR (300 MHz, $CDCl_3$)

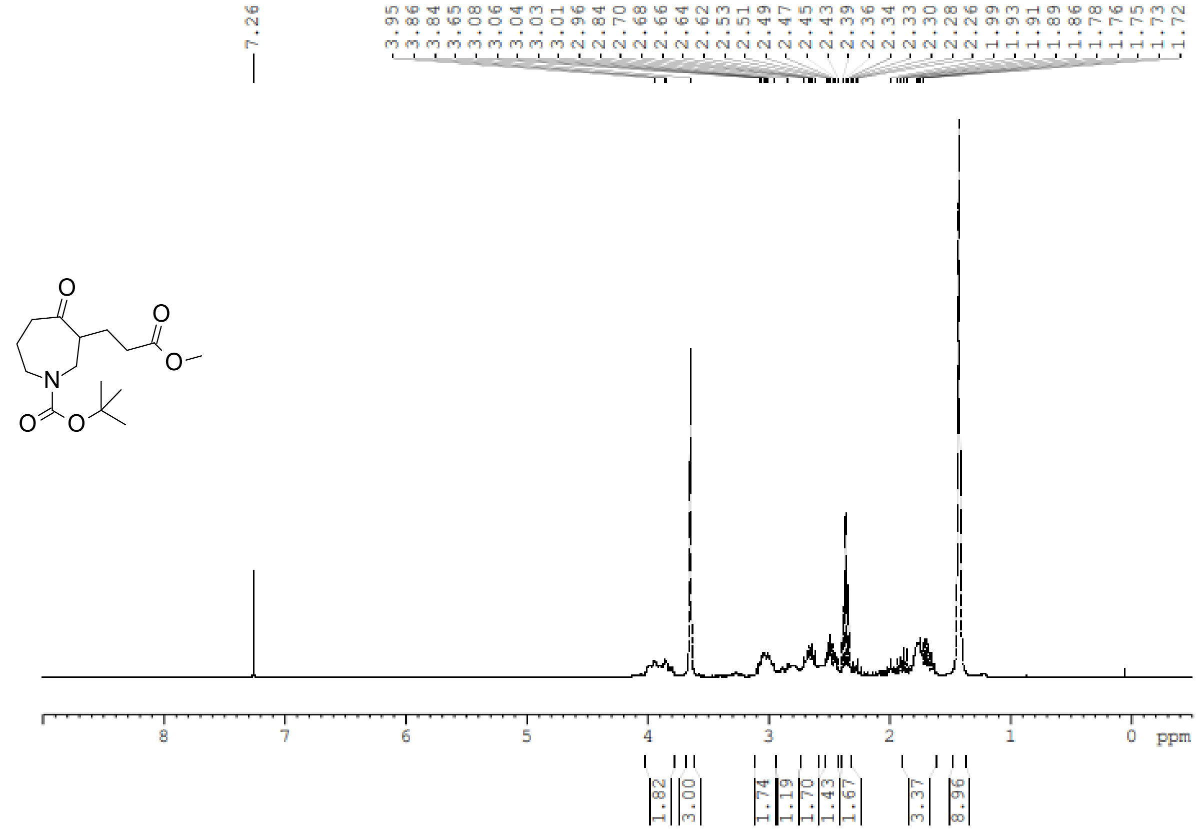

34, ^{13}C NMR (75 MHz, $CDCl_3$)

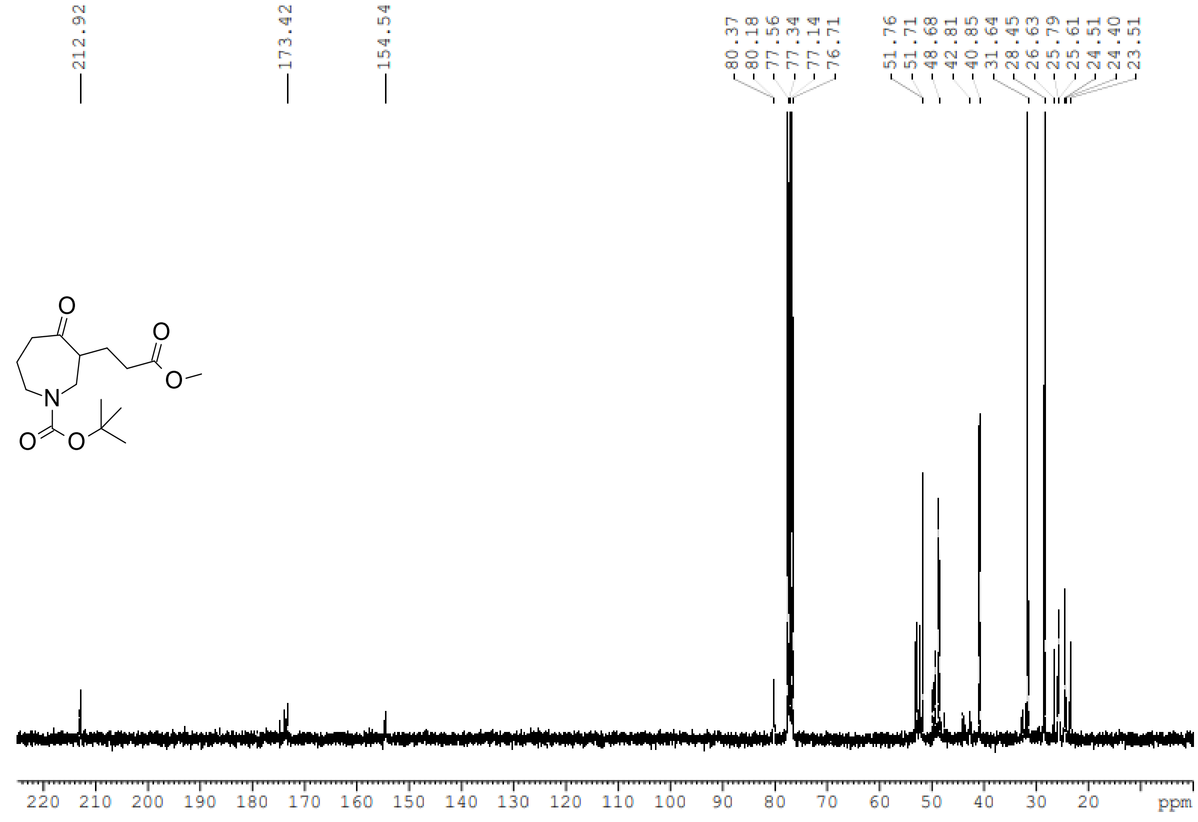

36, ^{1}H NMR (300 MHz, $CDCl_3$)

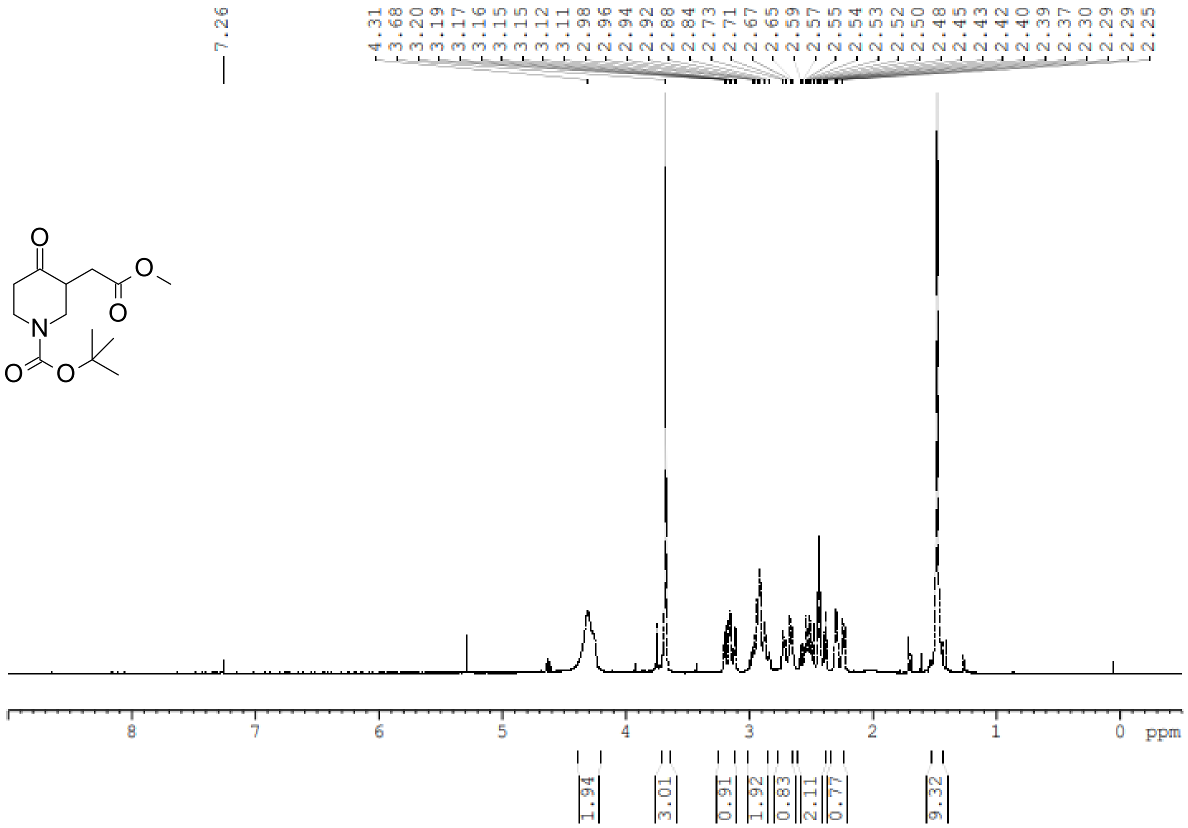

36, ^{13}C NMR (75 MHz, $CDCl_3$)

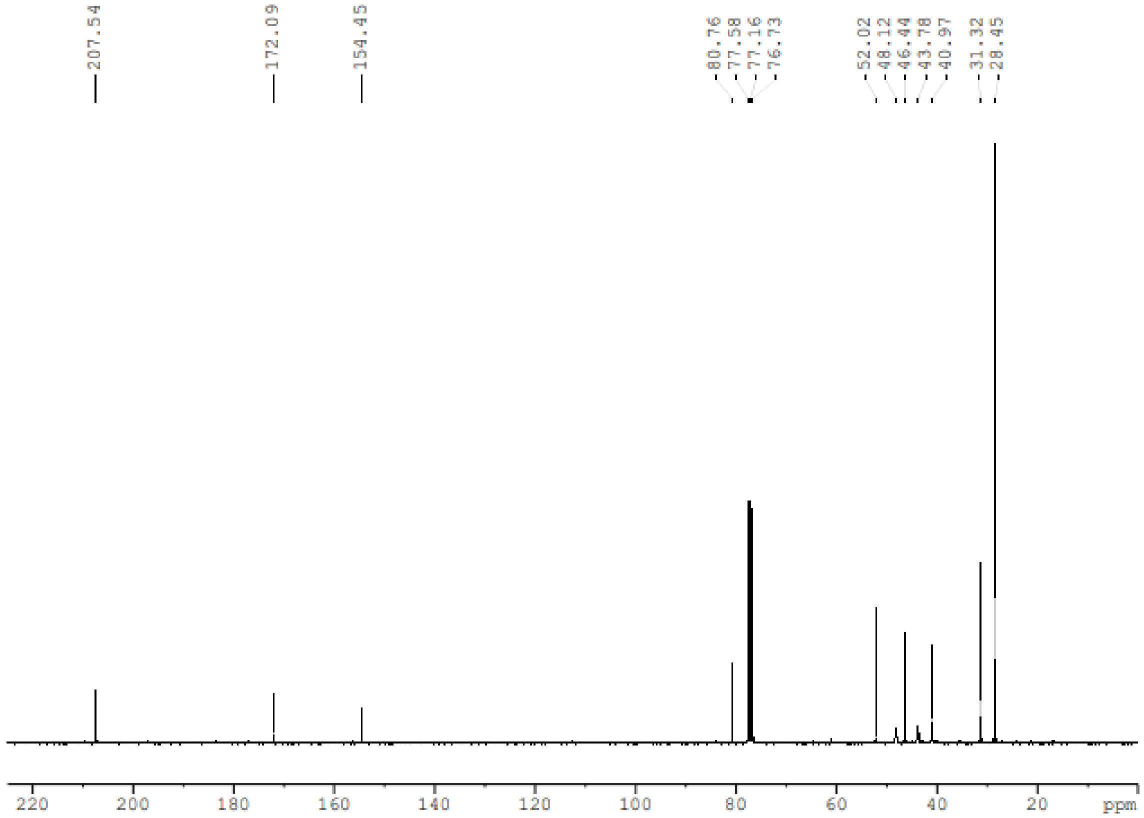

38, ^{1}H NMR (300 MHz, $CDCl_3$)

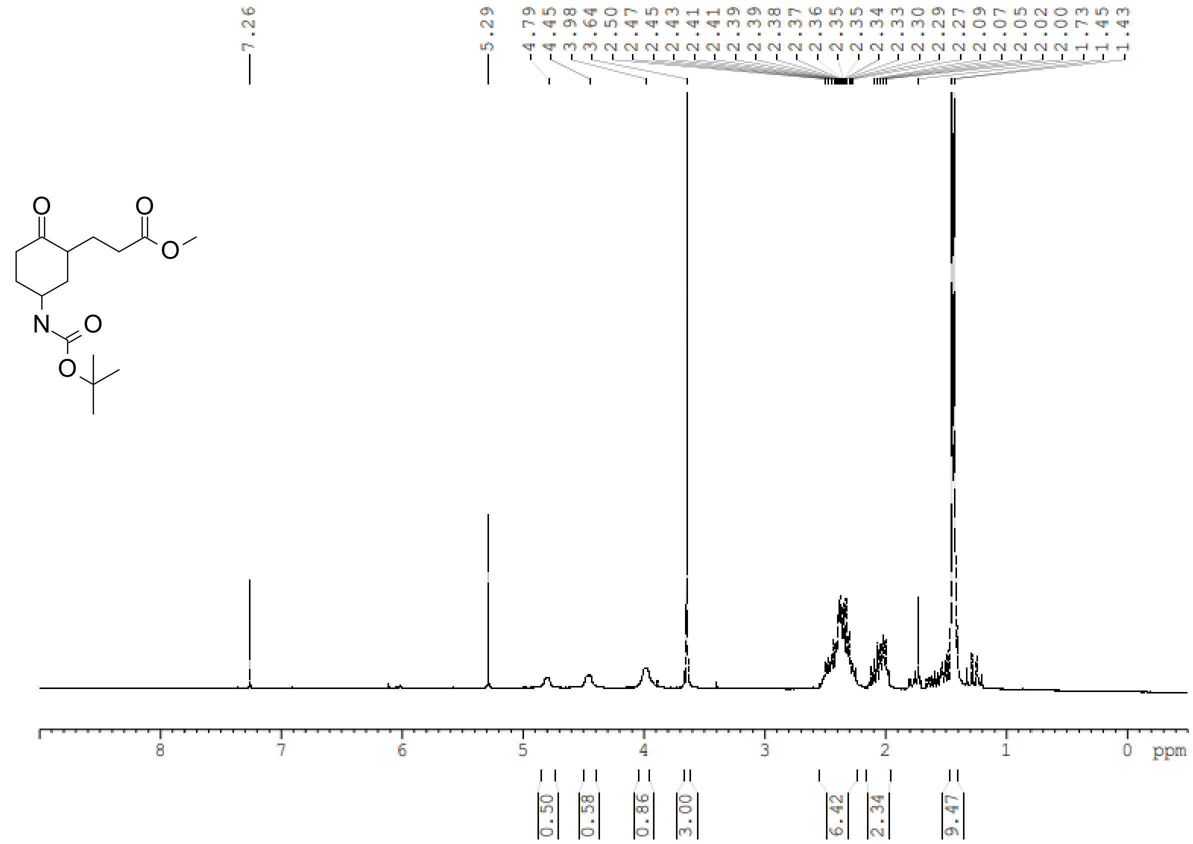

11, ^{1}H NMR (300 MHz, CD_2Cl_2)

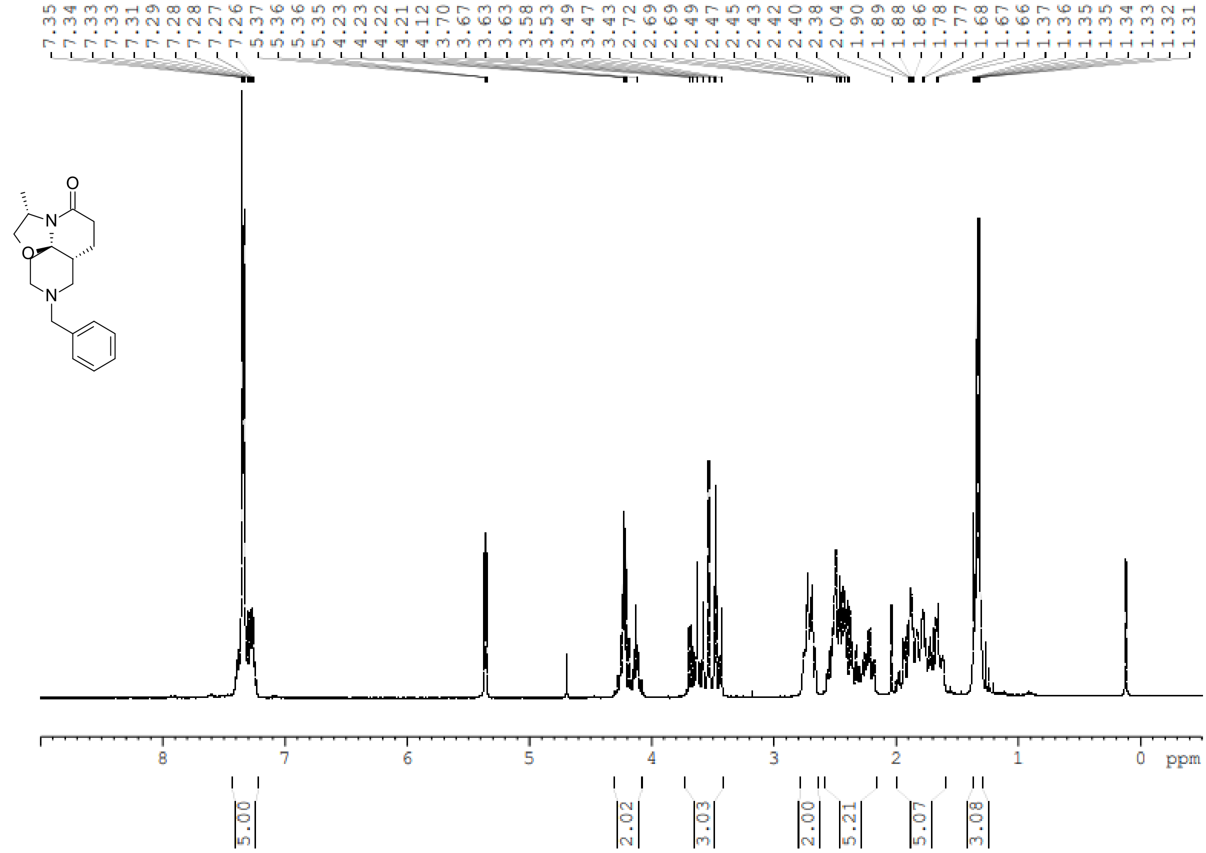

11, ^{13}C NMR (75 MHz, CD_2Cl_2)

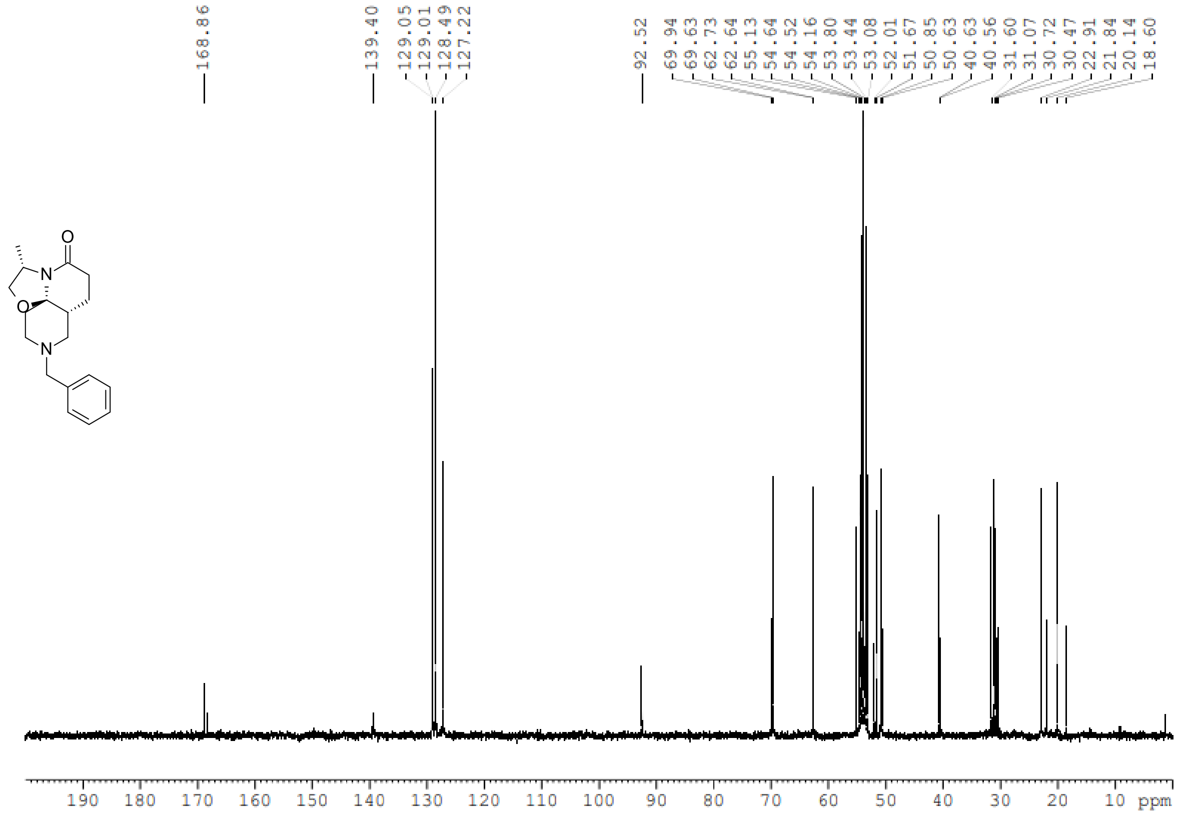

12, ^{1}H NMR (300 MHz, $CDCl_3$)

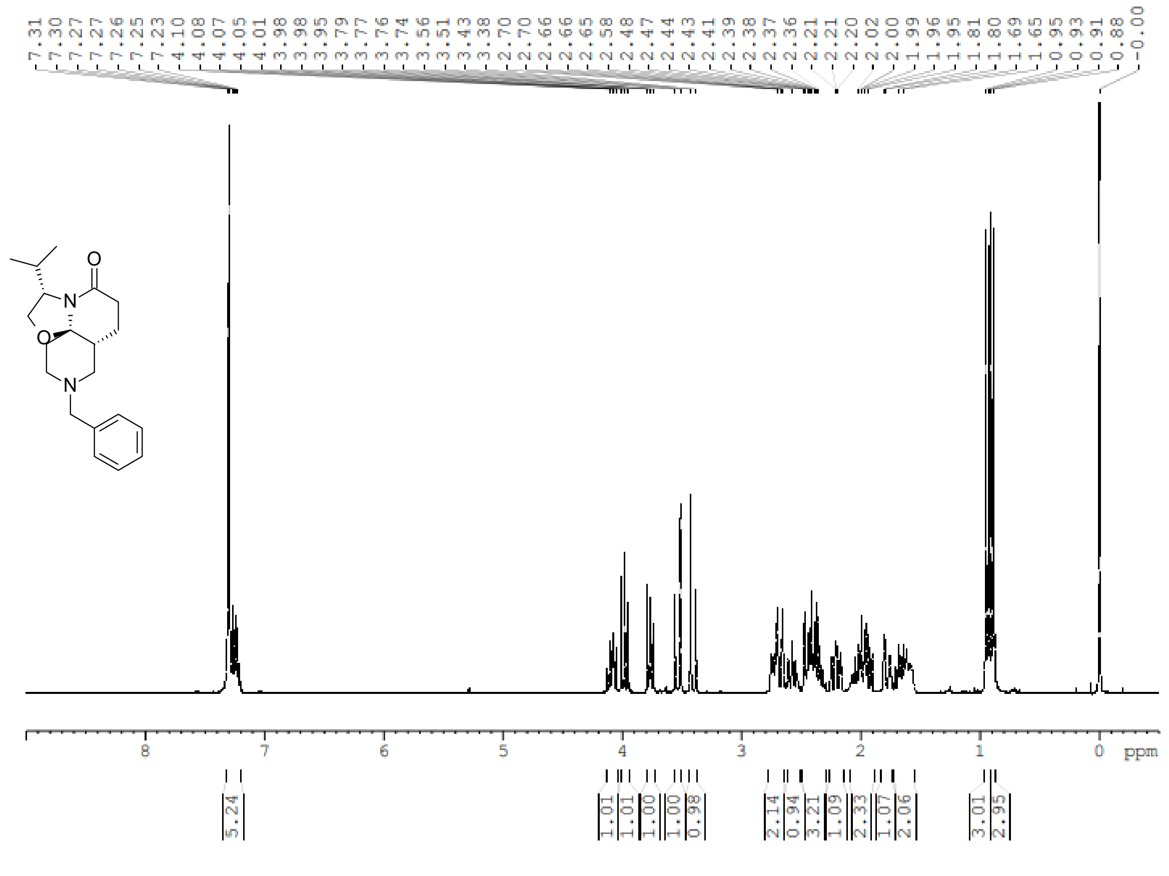

12, ^{13}C NMR (75 MHz, $CDCl_3$)

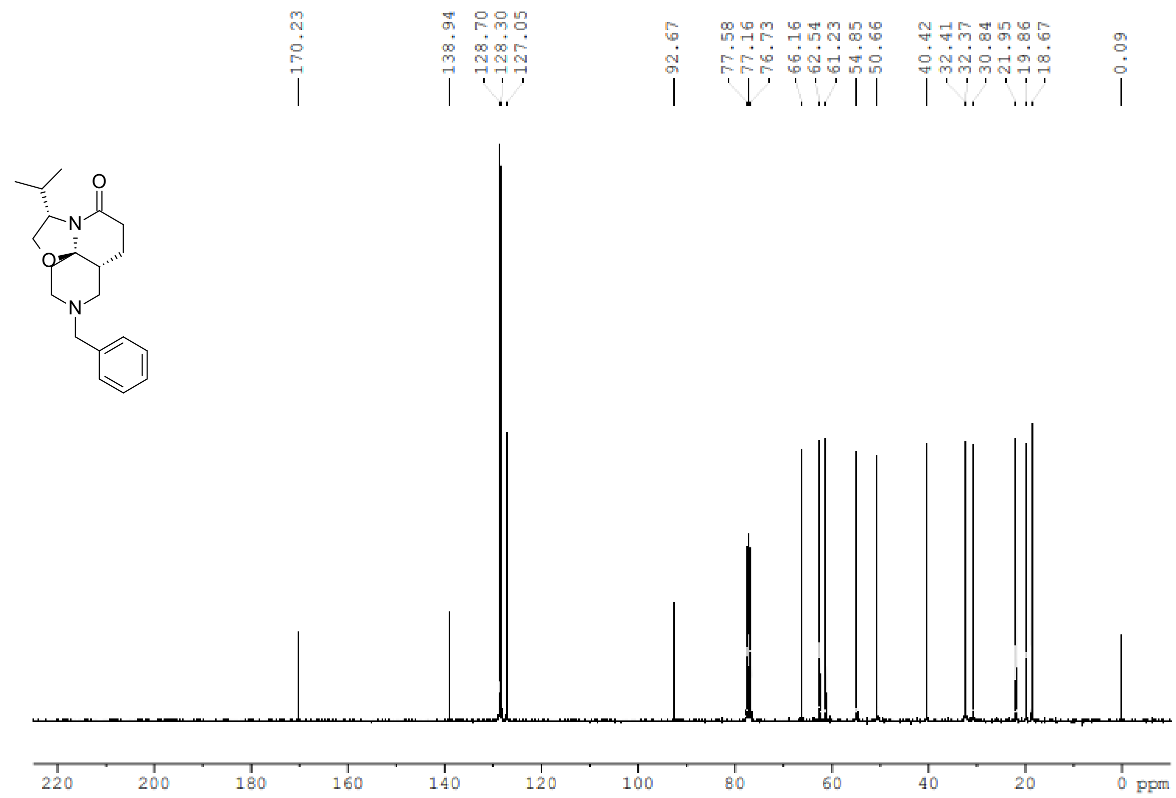

13, ^{1}H NMR (300 MHz, CD_2Cl_2)

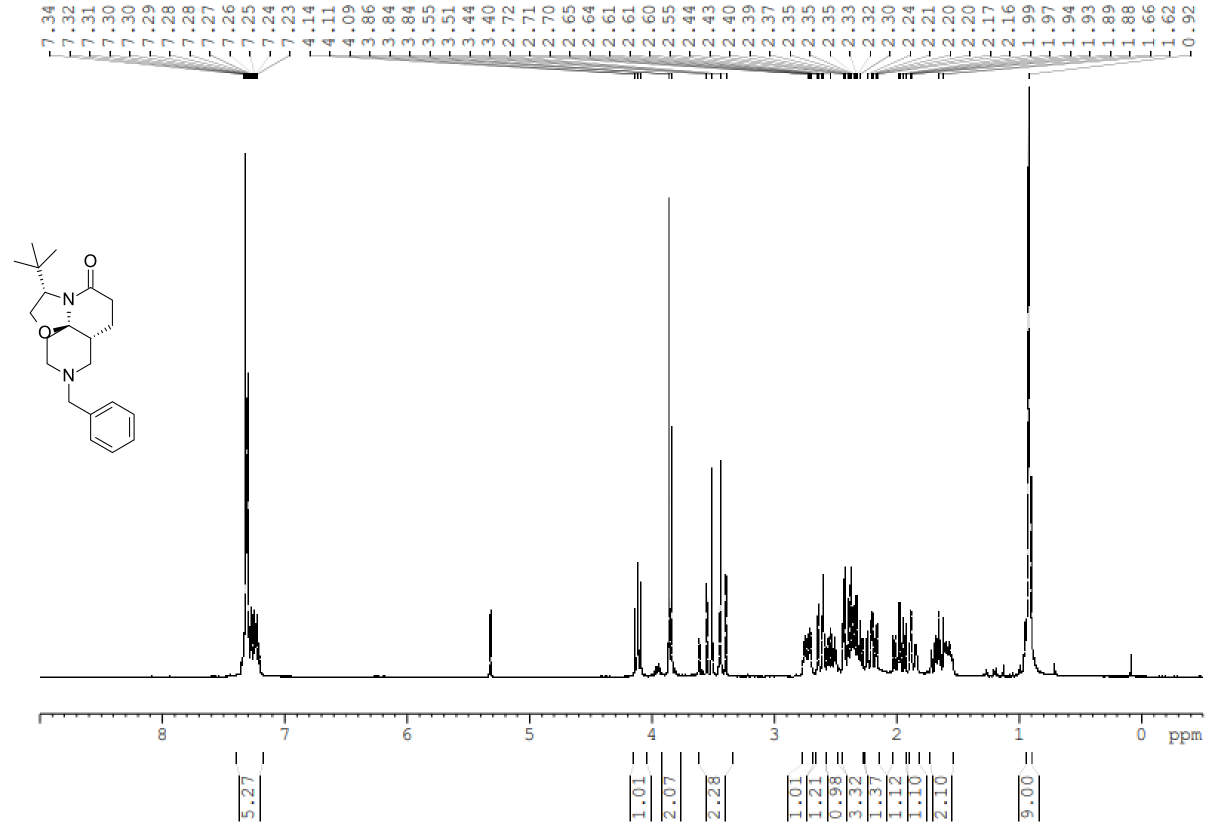

13, ^{13}C NMR (75 MHz, CD_2Cl_2)

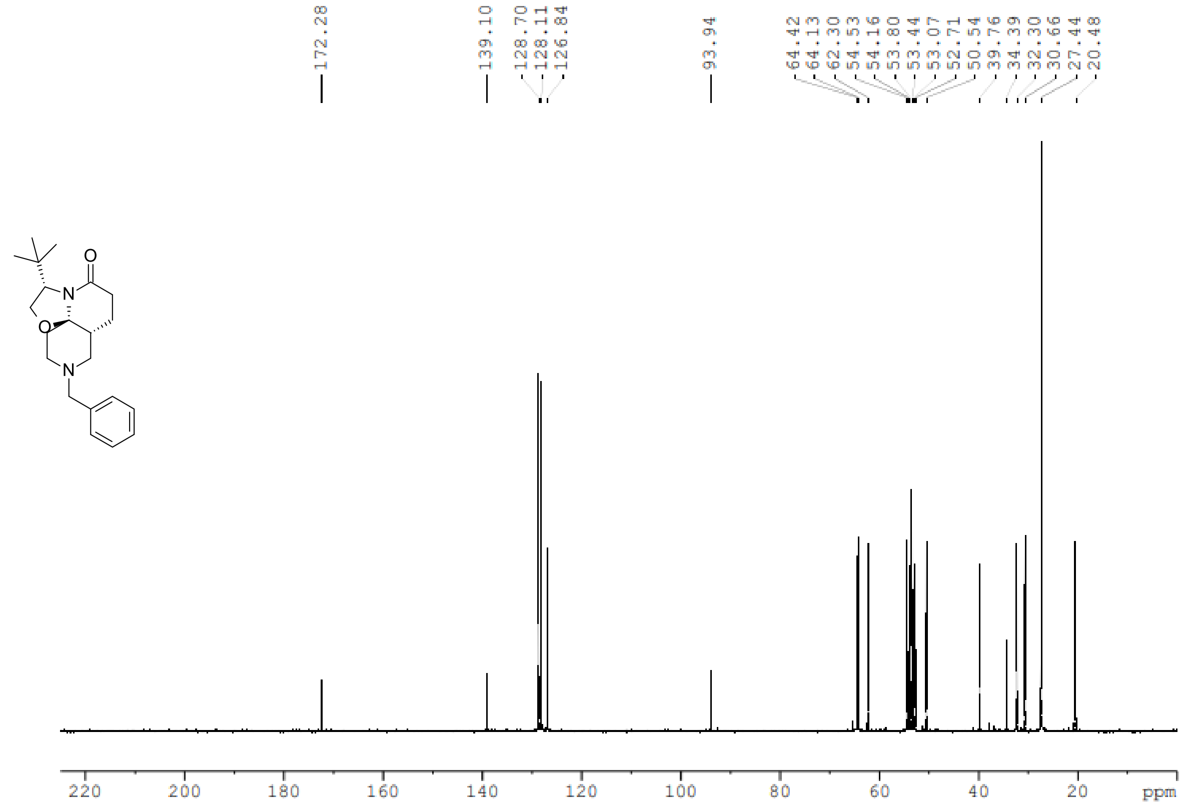

17, ^{1}H NMR (300 MHz, $CDCl_3$)

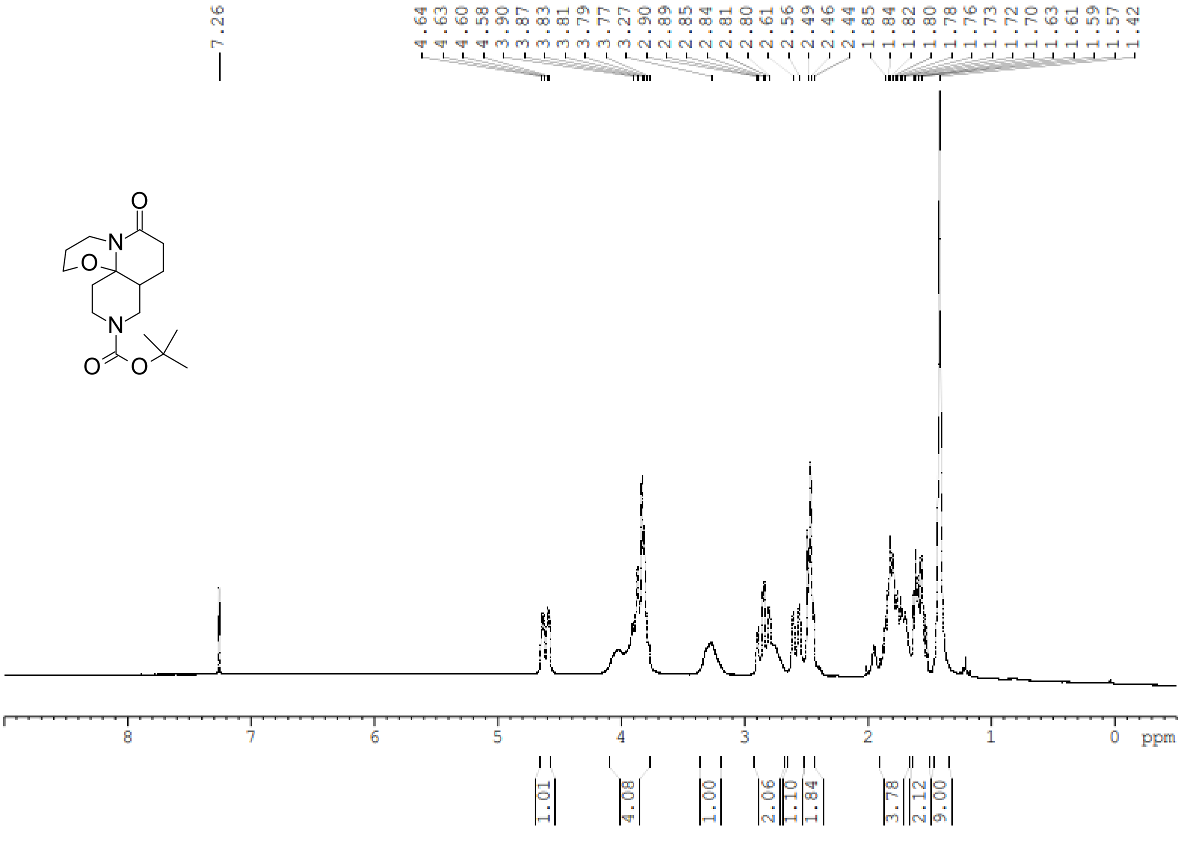

17, ^{13}C NMR (75 MHz, $CDCl_3$)

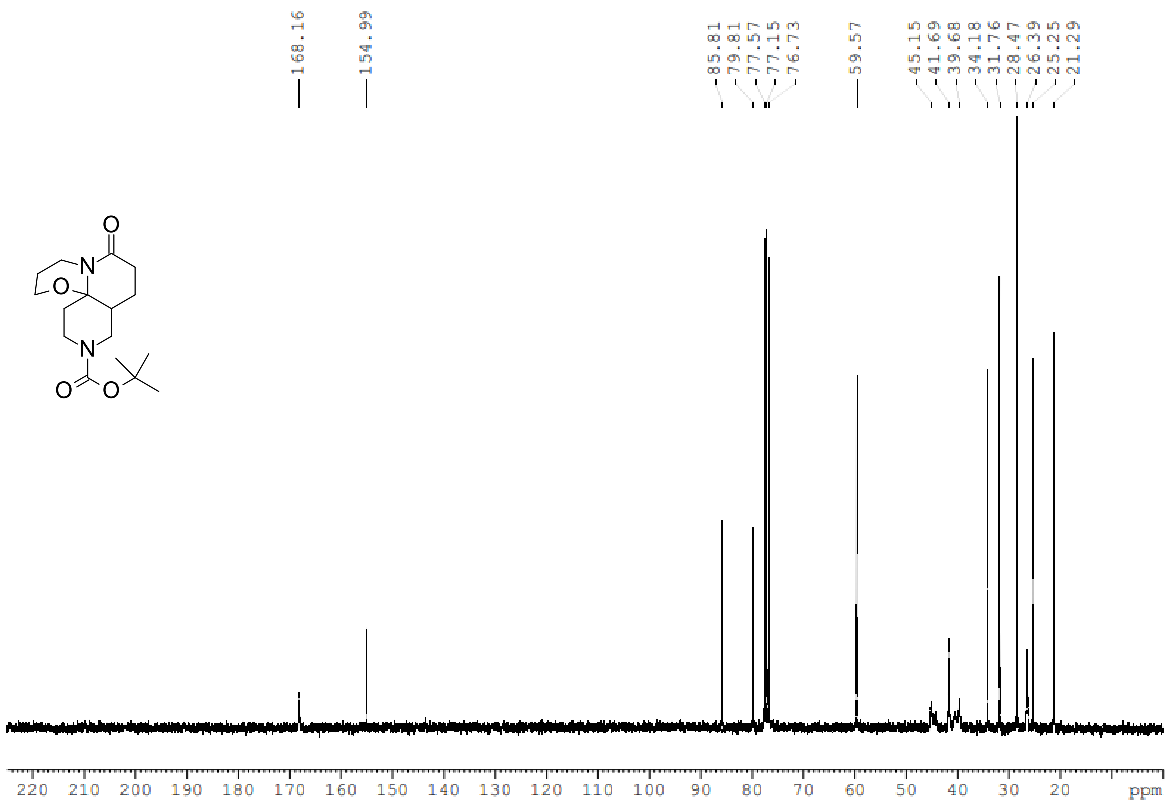

18, ^{1}H NMR (300 MHz, $CDCl_3$)

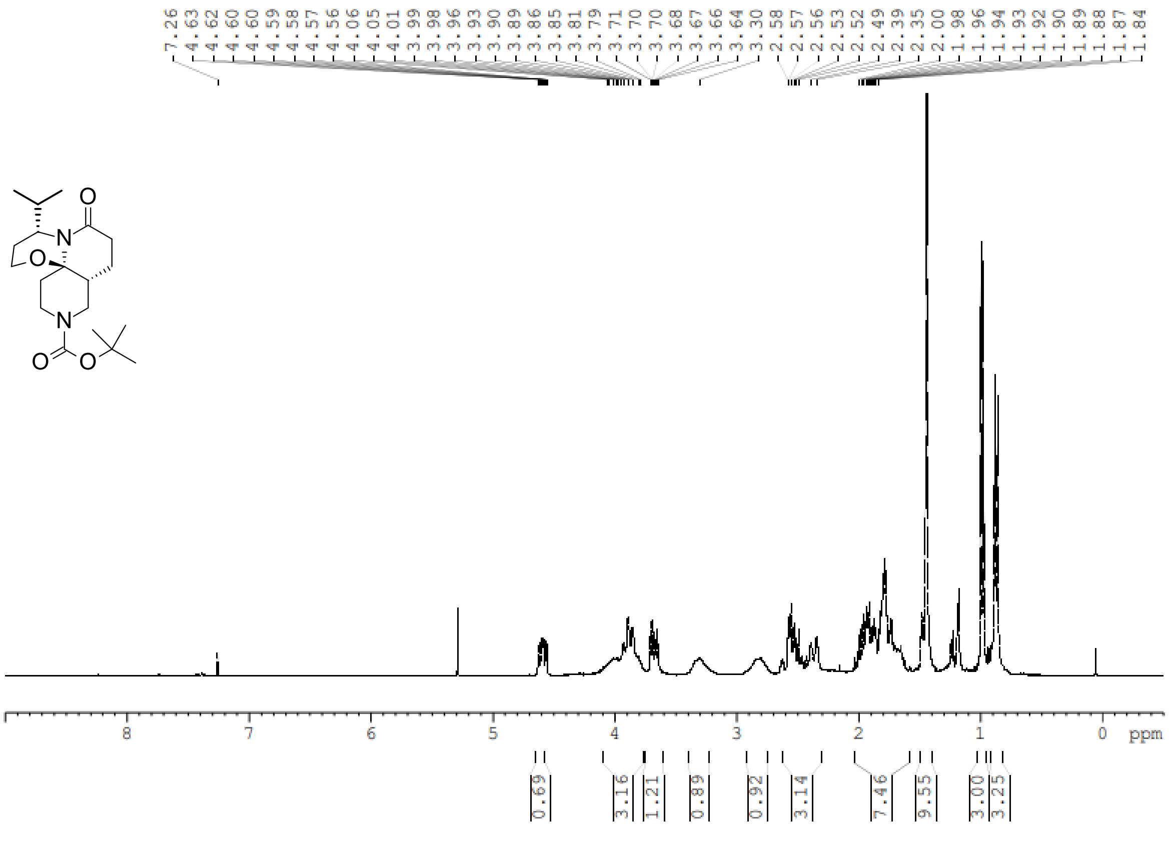

18, ^{13}C NMR (75 MHz, $CDCl_3$)

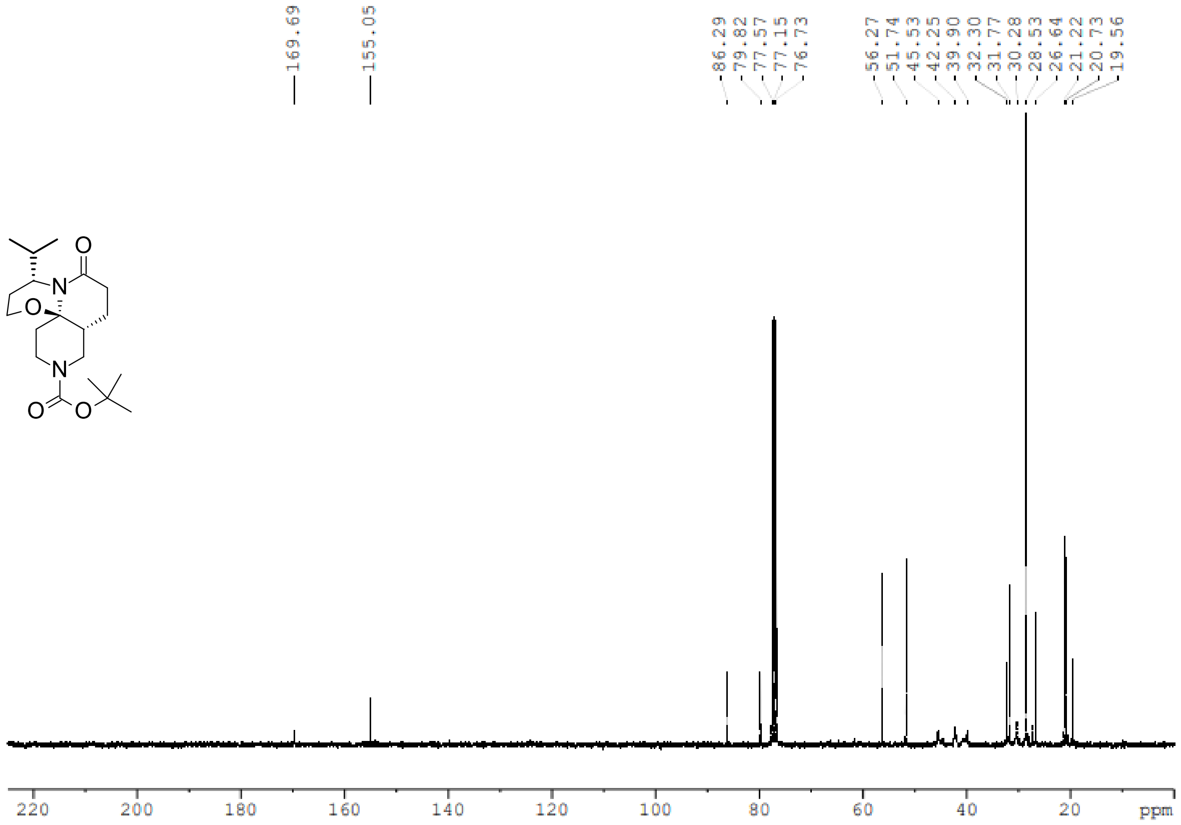

19+19', ^{1}H NMR (300 MHz, $CDCl_3$)

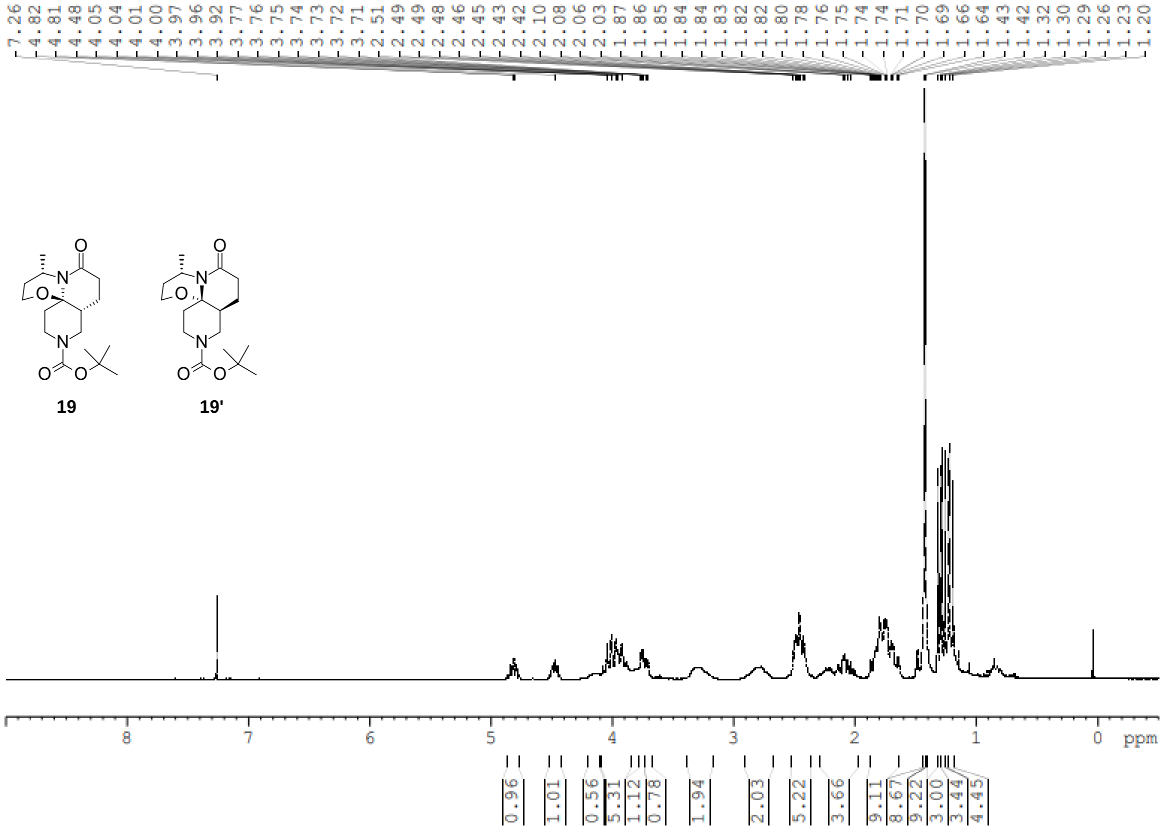

19+19', ^{13}C NMR (75 MHz, $CDCl_3$)

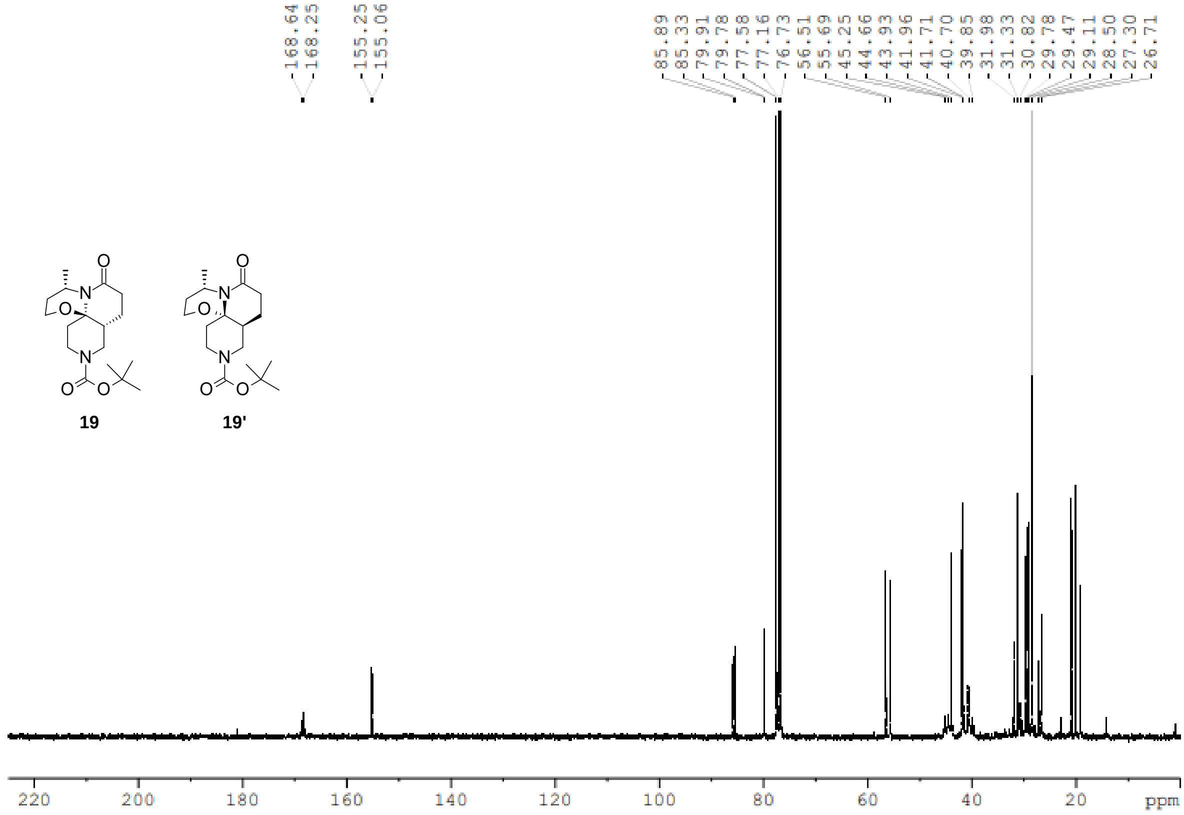

20, ^{1}H NMR (300 MHz, $CDCl_3$)

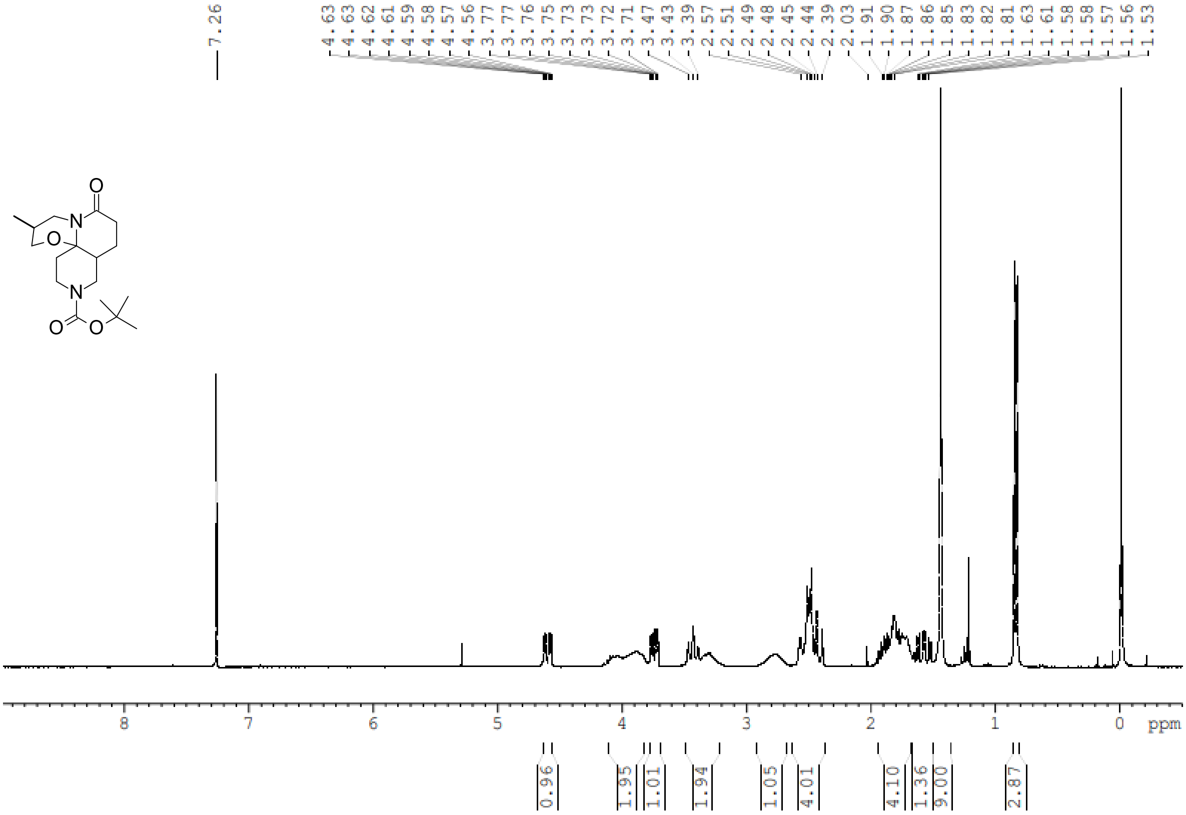

20, ^{13}C NMR (75 MHz, $CDCl_3$)

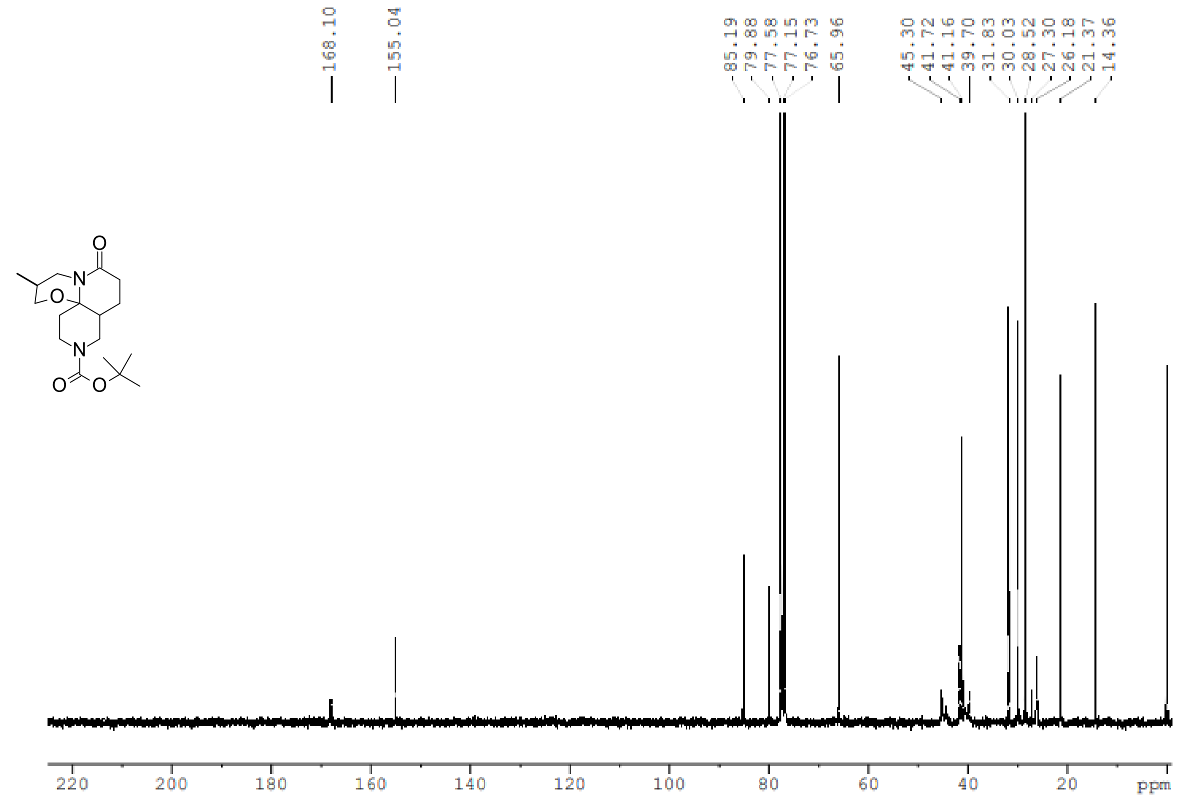

21, ^{1}H NMR (300 MHz, $CDCl_3$)

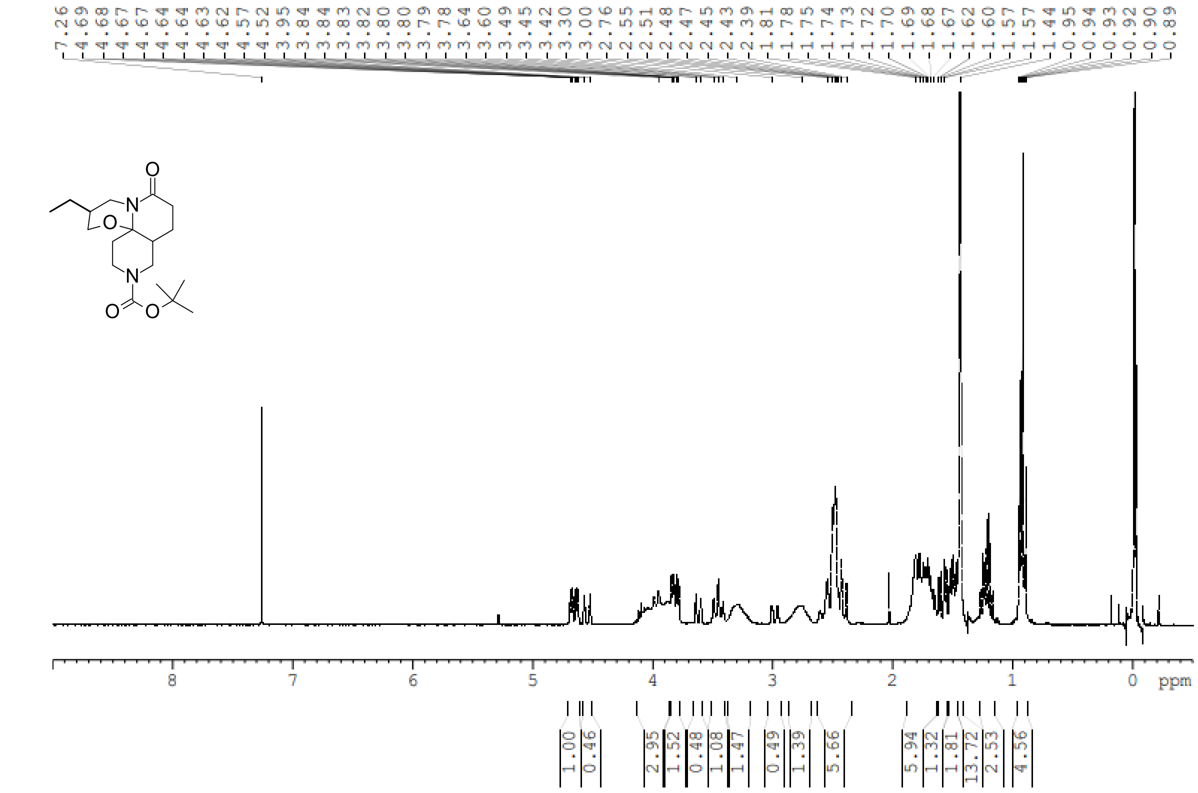

21, ^{13}C NMR (75 MHz, CDCl$_3$)

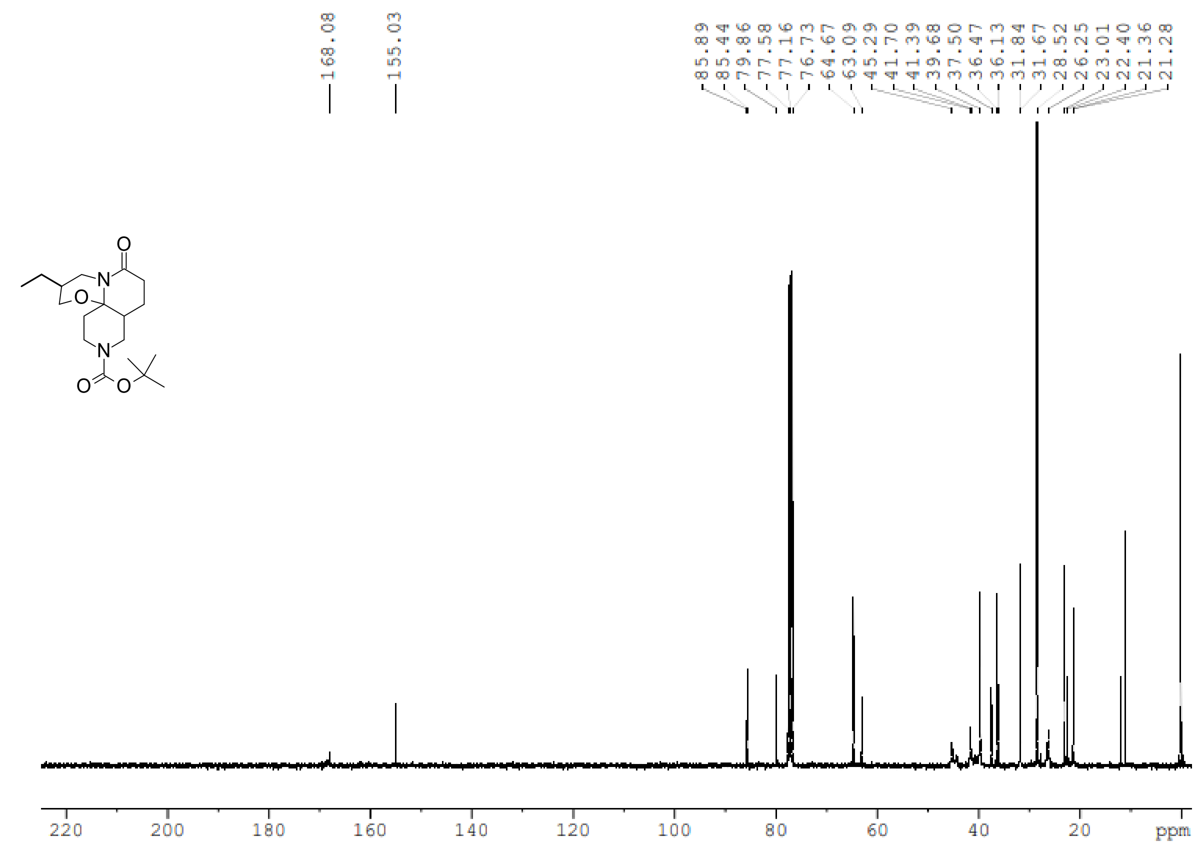

22, ^{1}H NMR (300 MHz, CDCl$_3$)

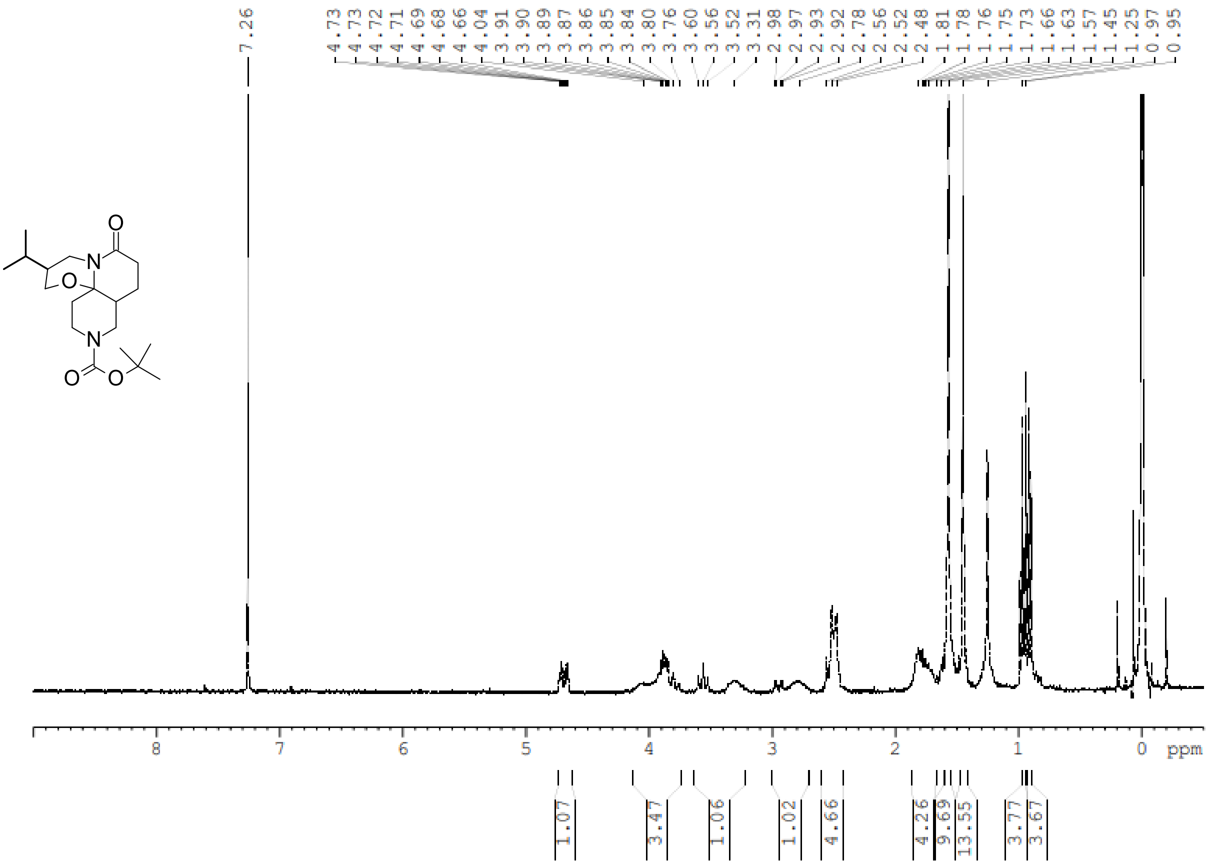

22, ^{13}C NMR (75 MHz, $CDCl_3$)

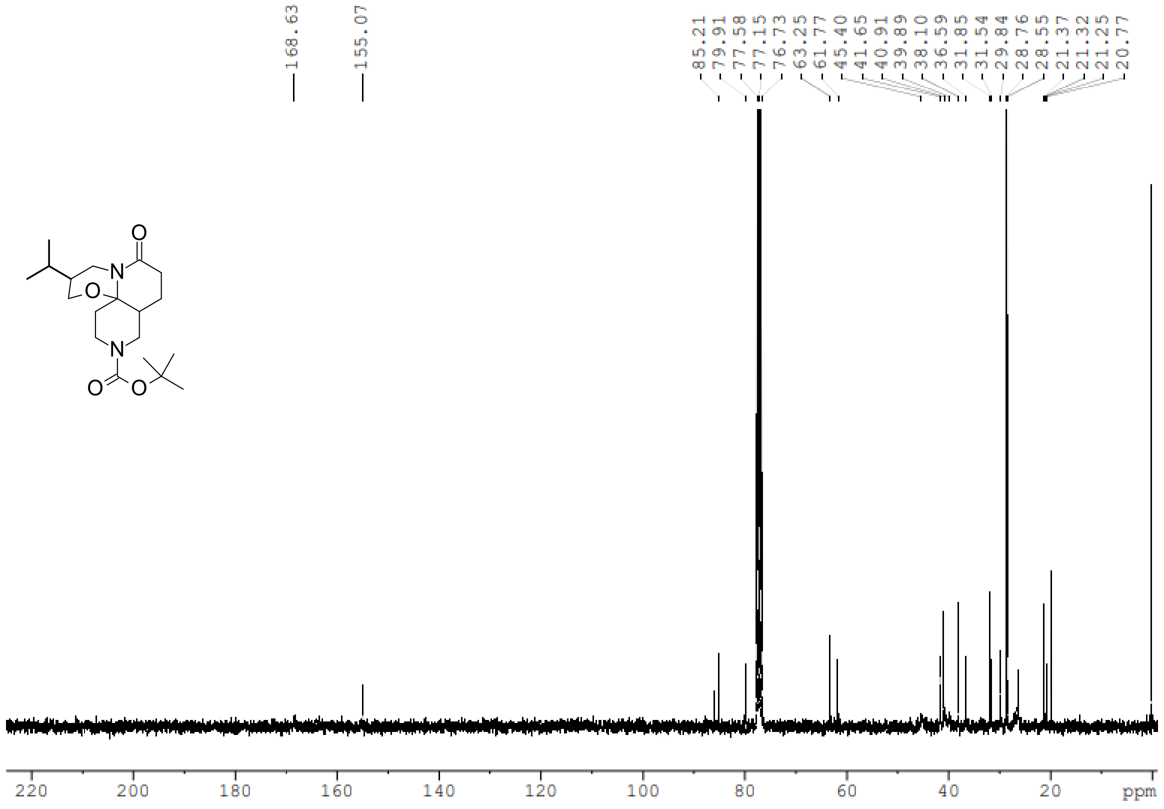

23, ^{1}H NMR (300 MHz, $CDCl_3$)

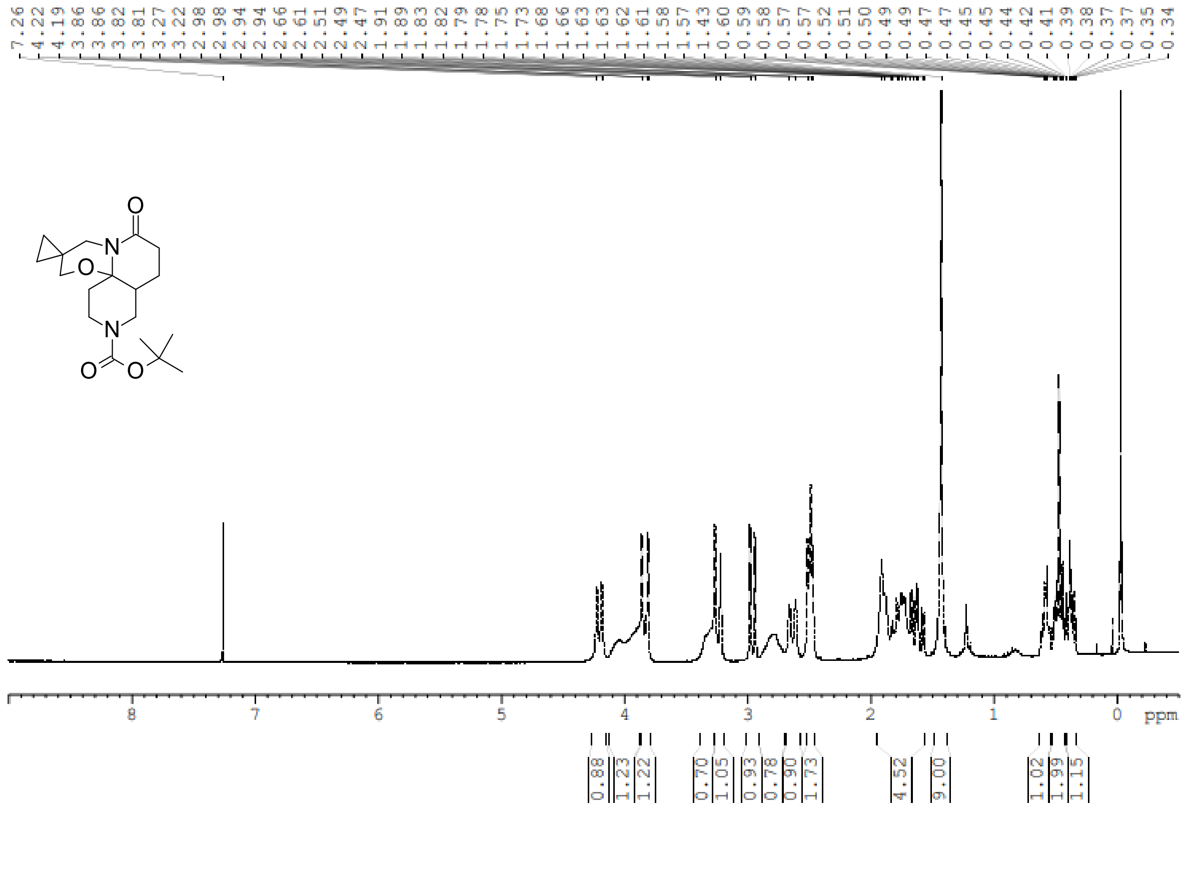

23, ^{13}C NMR (75 MHz, $CDCl_3$)

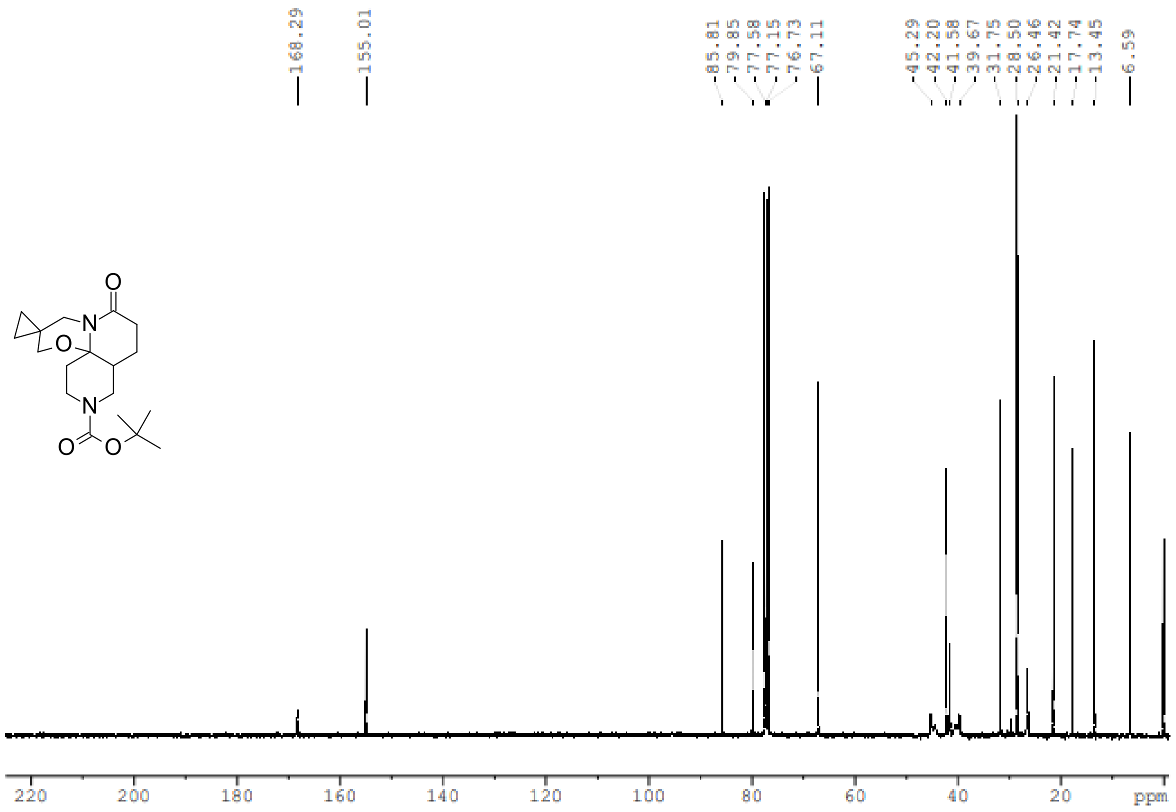

24, ^{1}H NMR (300 MHz, $CDCl_3$)

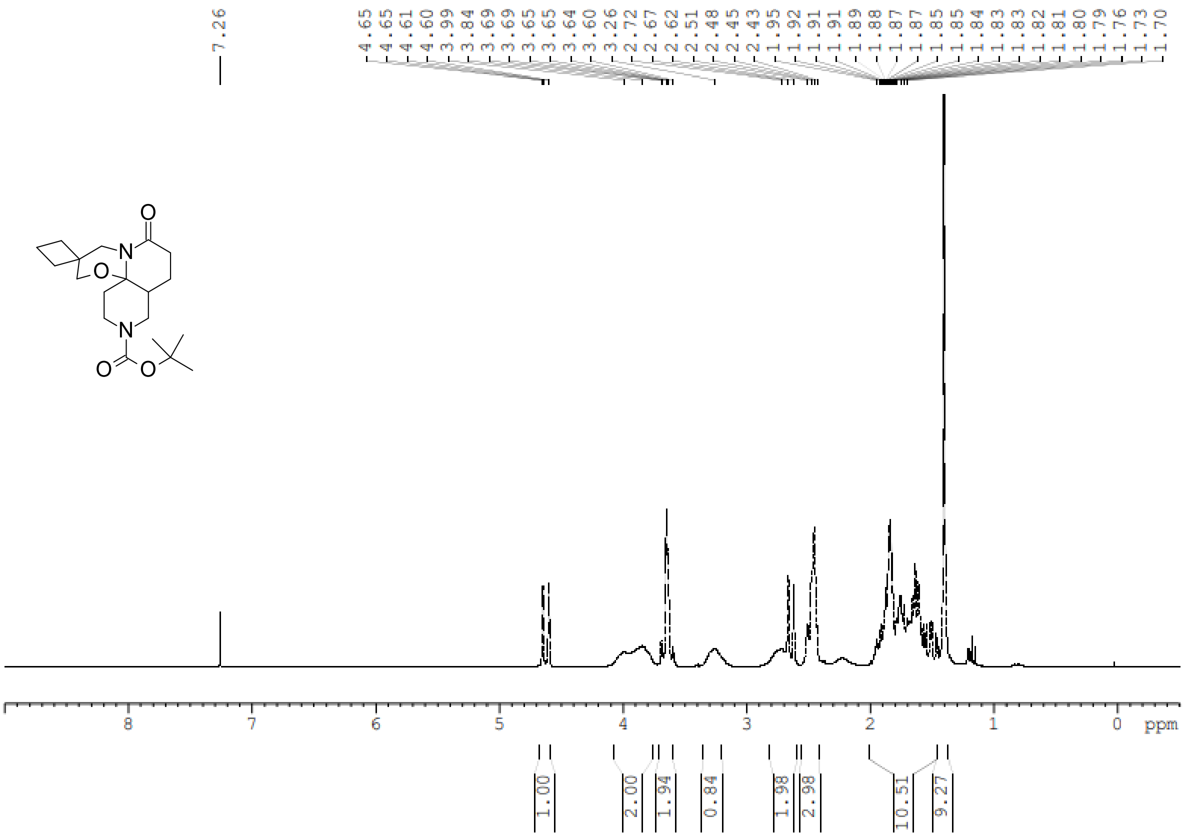

24, ^{13}C NMR (75 MHz, $CDCl_3$)

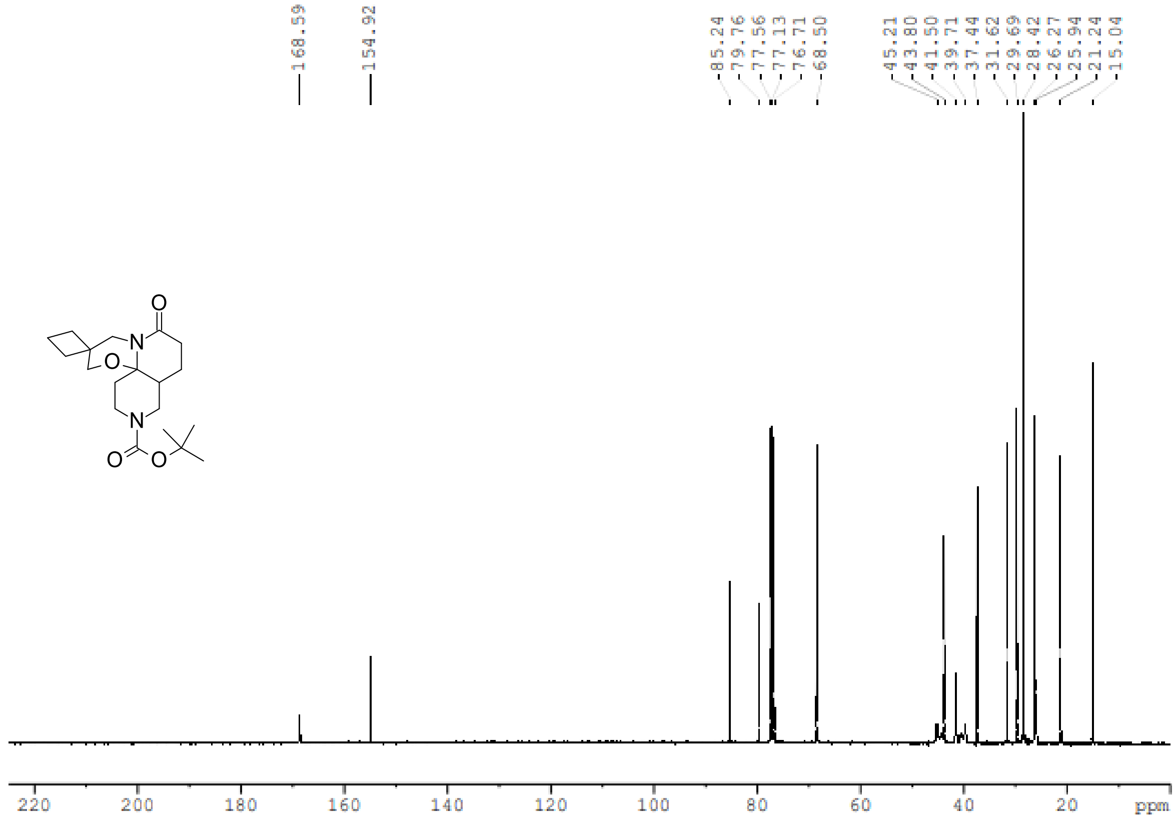

25, ^{1}H NMR (300 MHz, $CDCl_3$)

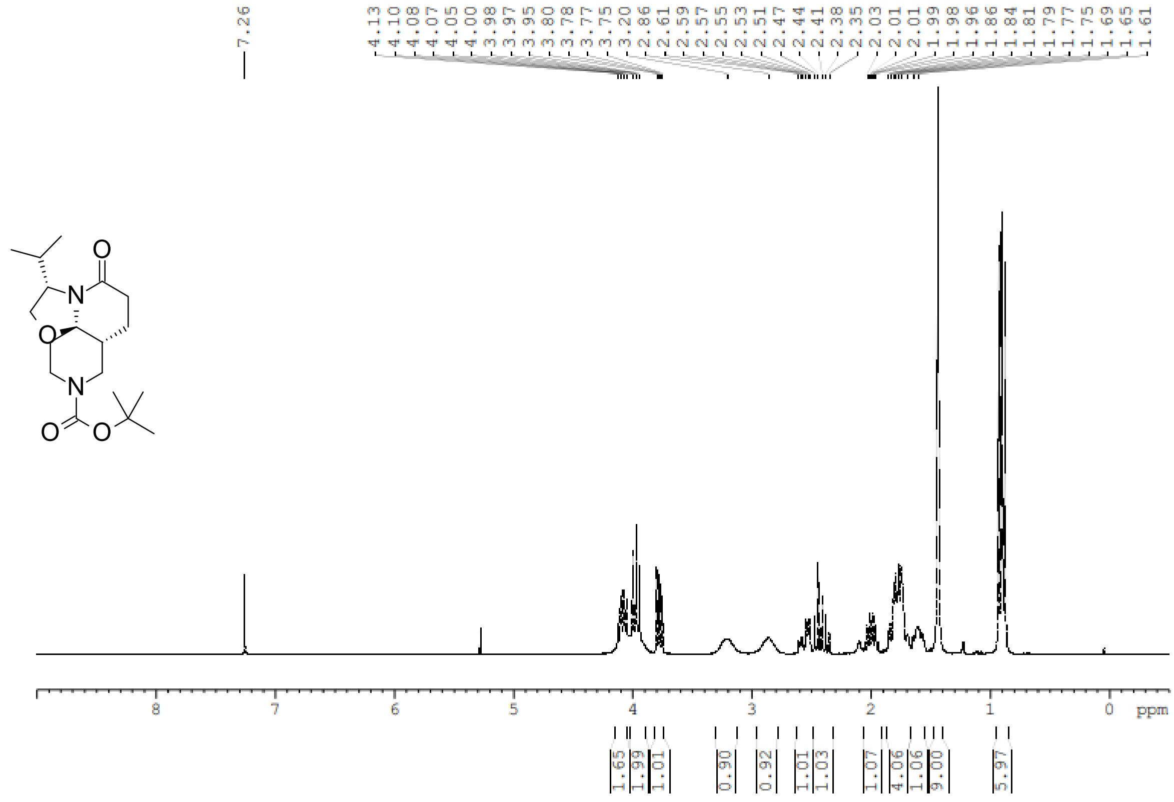

25, ^{13}C NMR (75 MHz, $CDCl_3$)

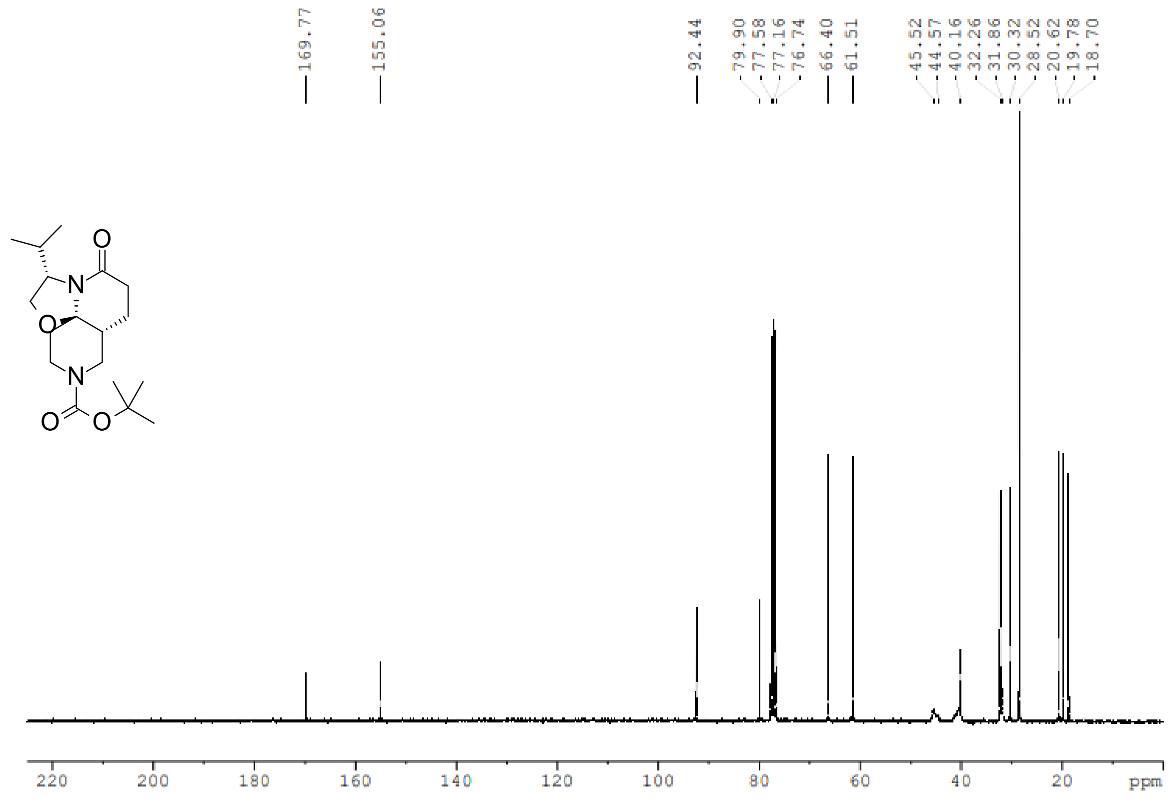

26, ^{1}H NMR (300 MHz, $CDCl_3$)

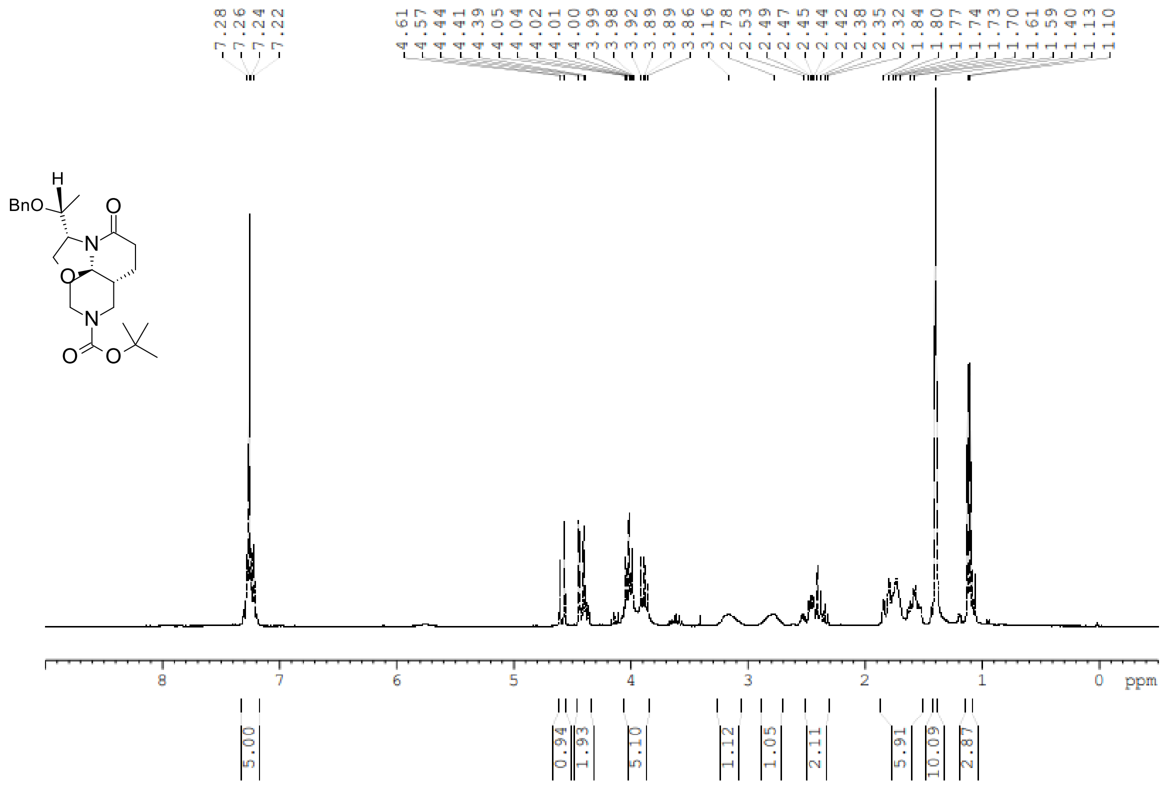

26, ^{13}C NMR (75 MHz, $CDCl_3$)

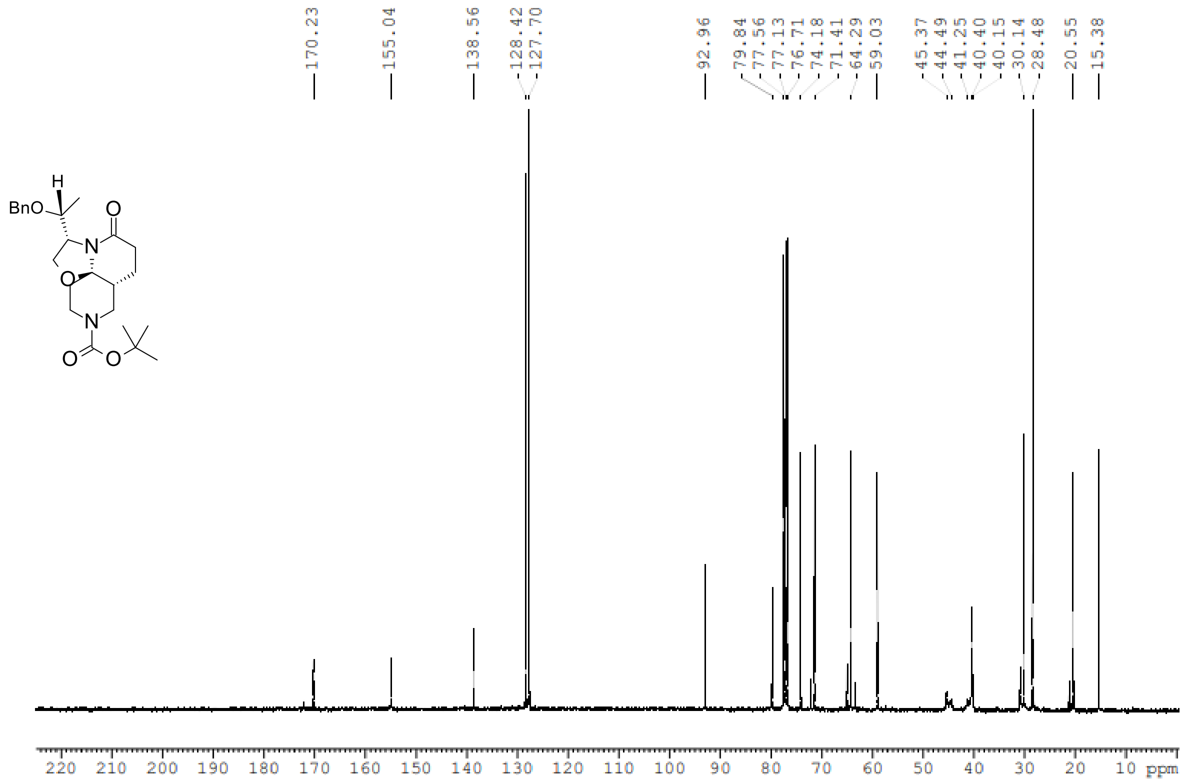

27, ^{1}H NMR (300 MHz, $CDCl_3$)

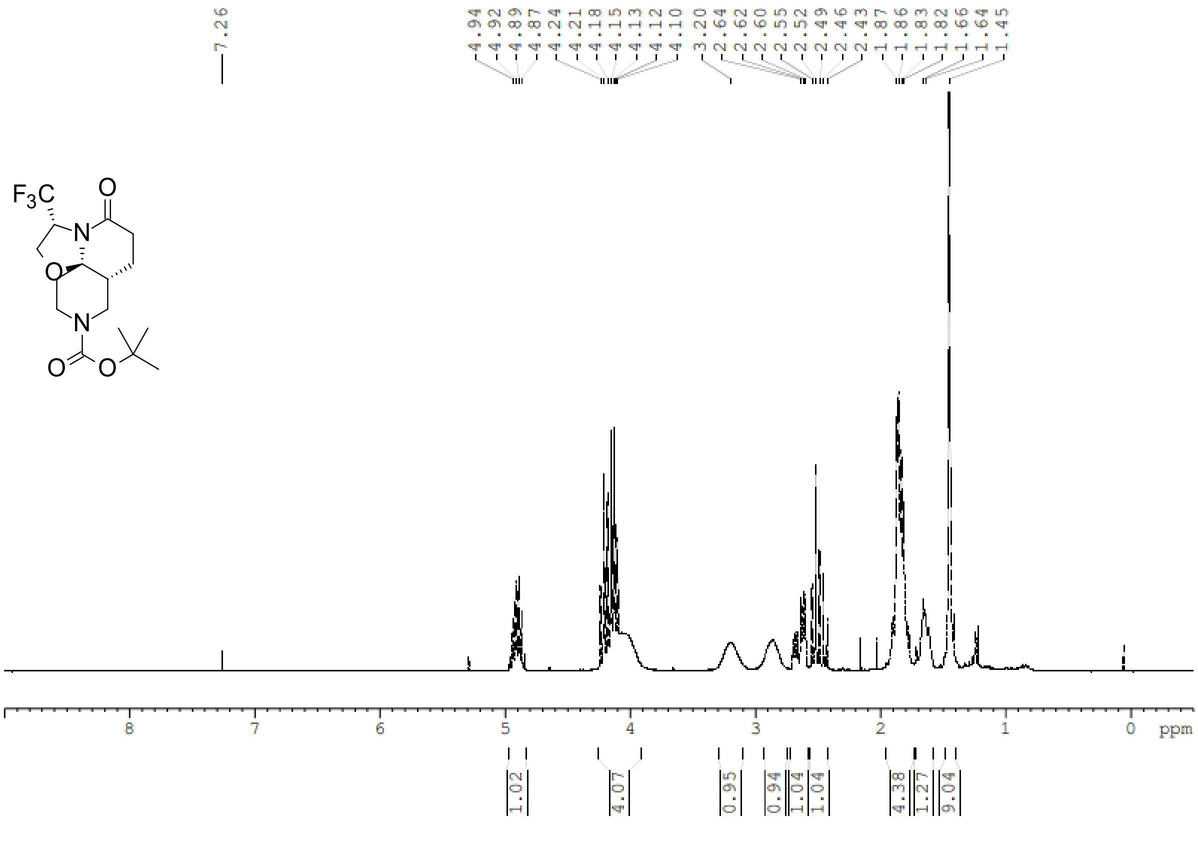

27, ^{13}C NMR (75 MHz, $CDCl_3$)

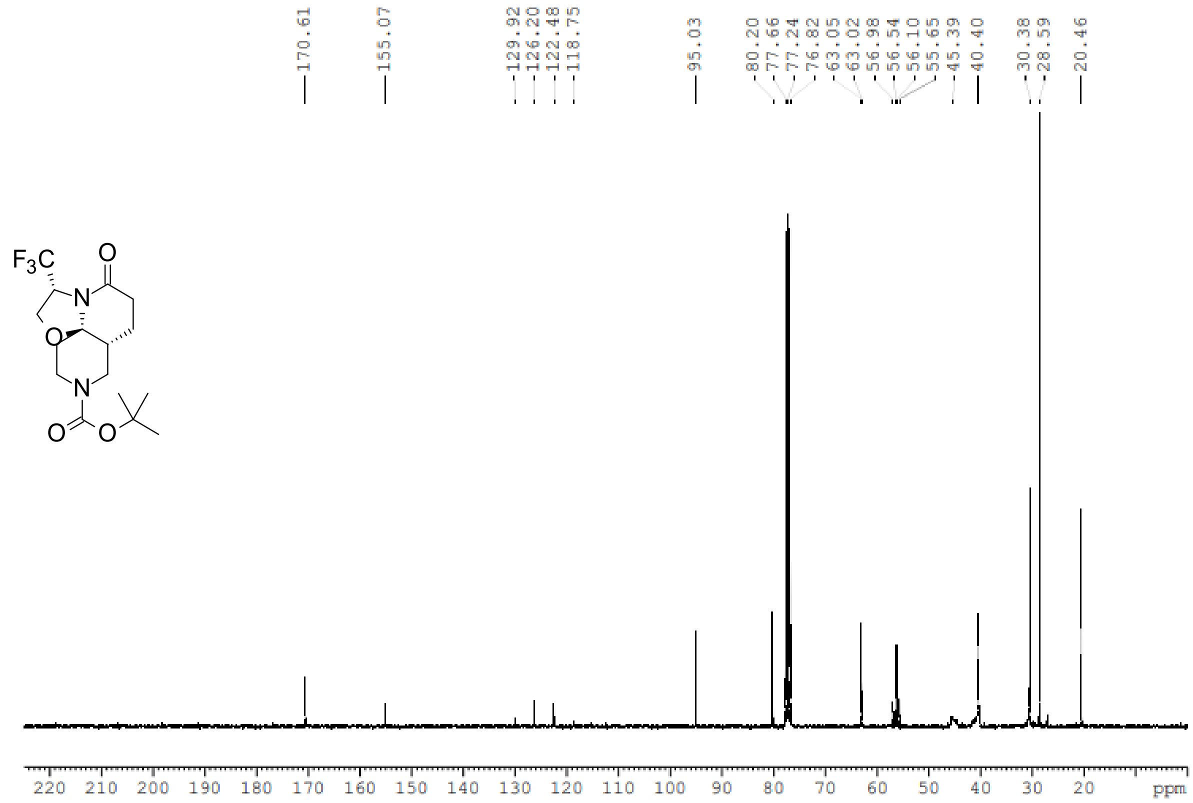

28, ^{1}H NMR (300 MHz, $CDCl_3$)

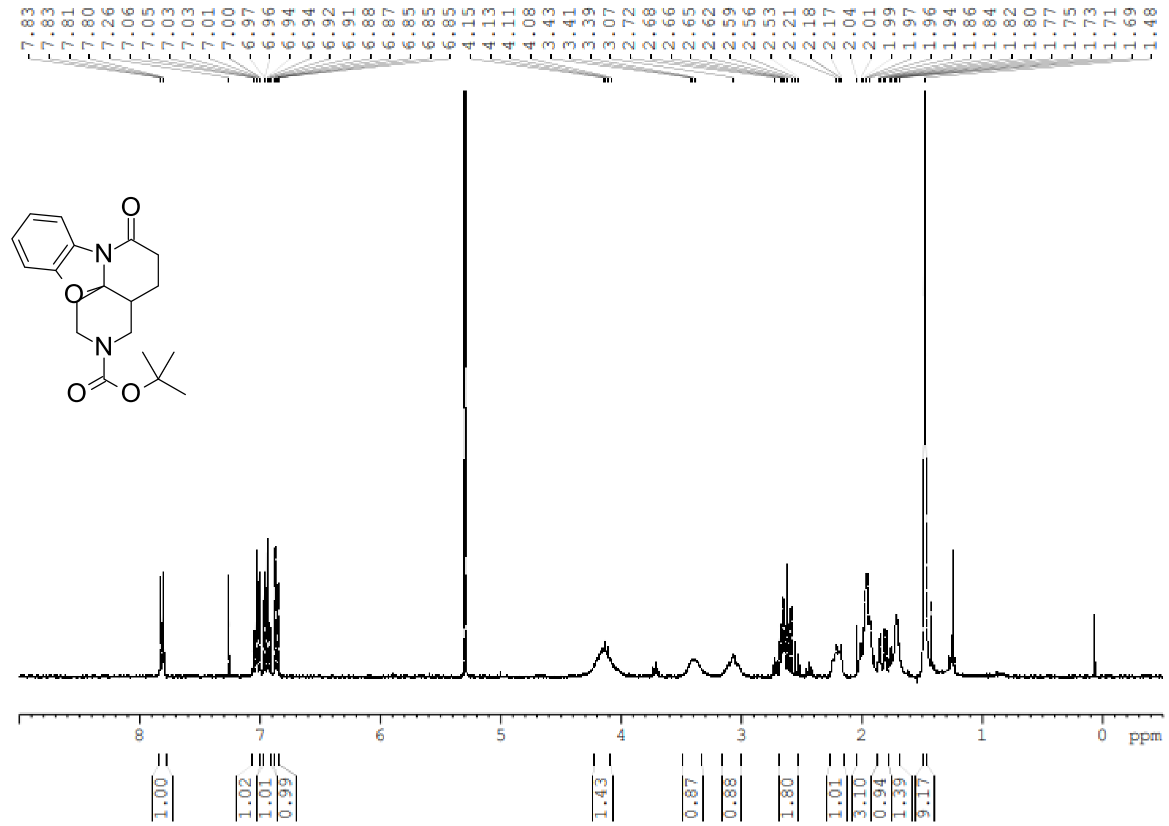

28, ^{13}C NMR (75 MHz, $CDCl_3$)

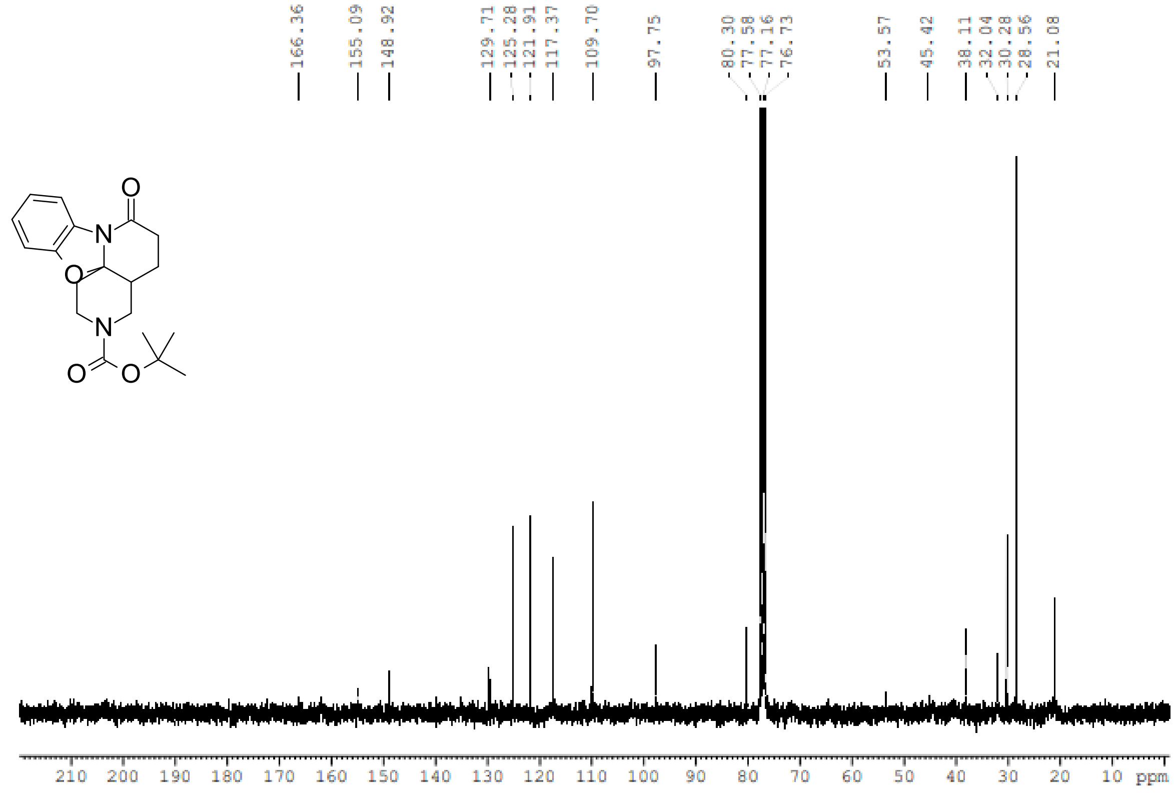

29, ^{1}H NMR (300 MHz, $CDCl_3$)

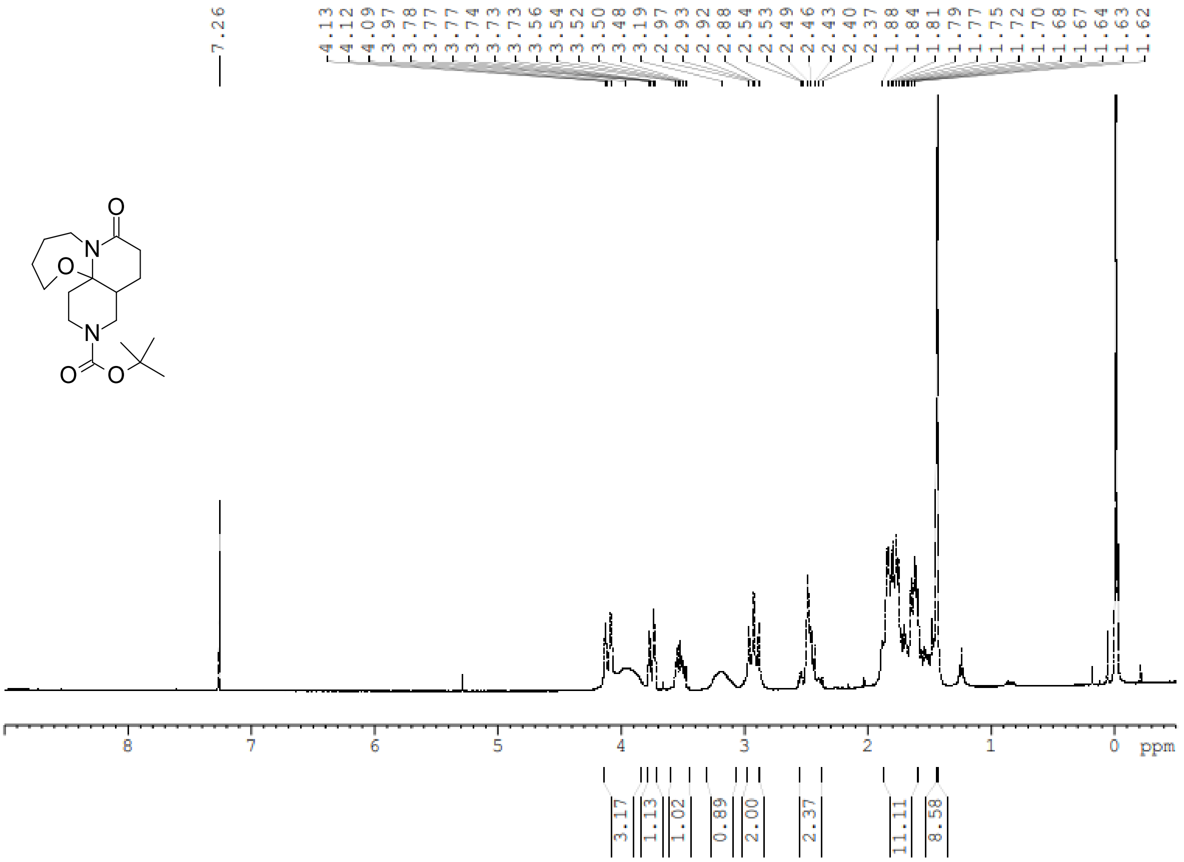

29, ^{13}C NMR (75 MHz, $CDCl_3$)

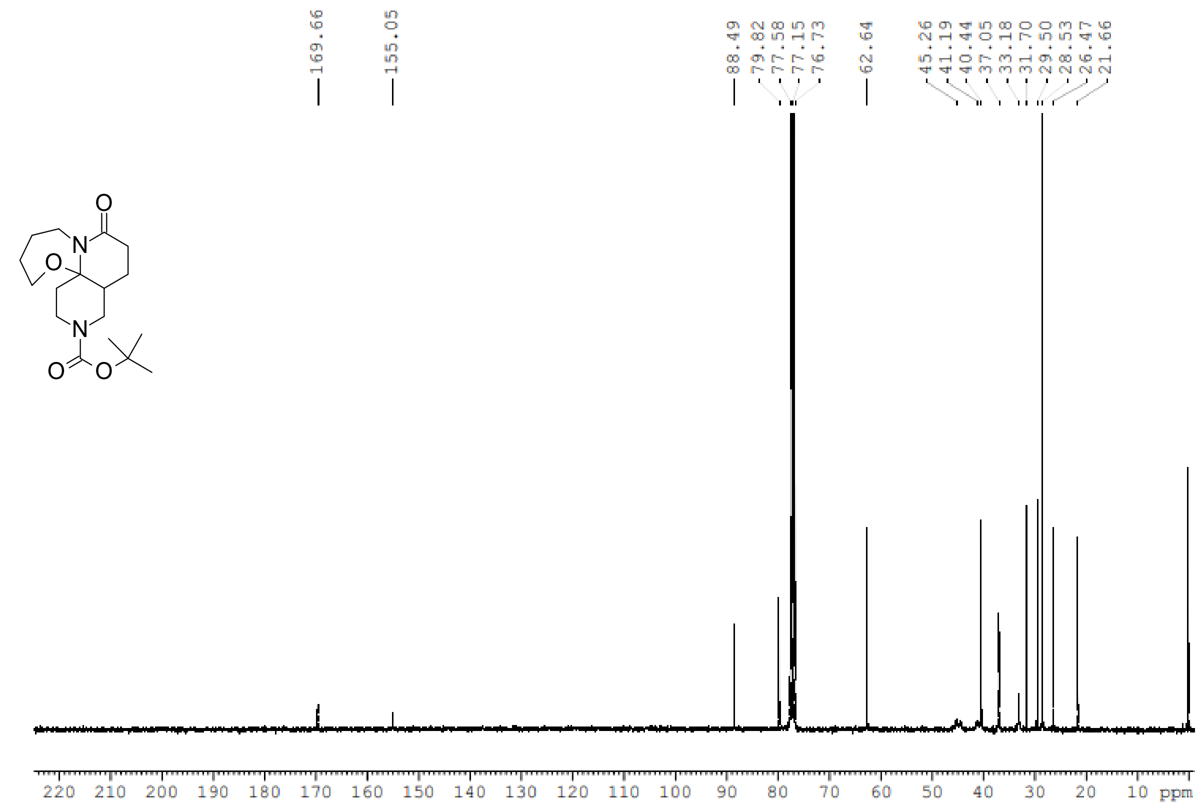

31, ^{1}H NMR (300 MHz, $CDCl_3$)

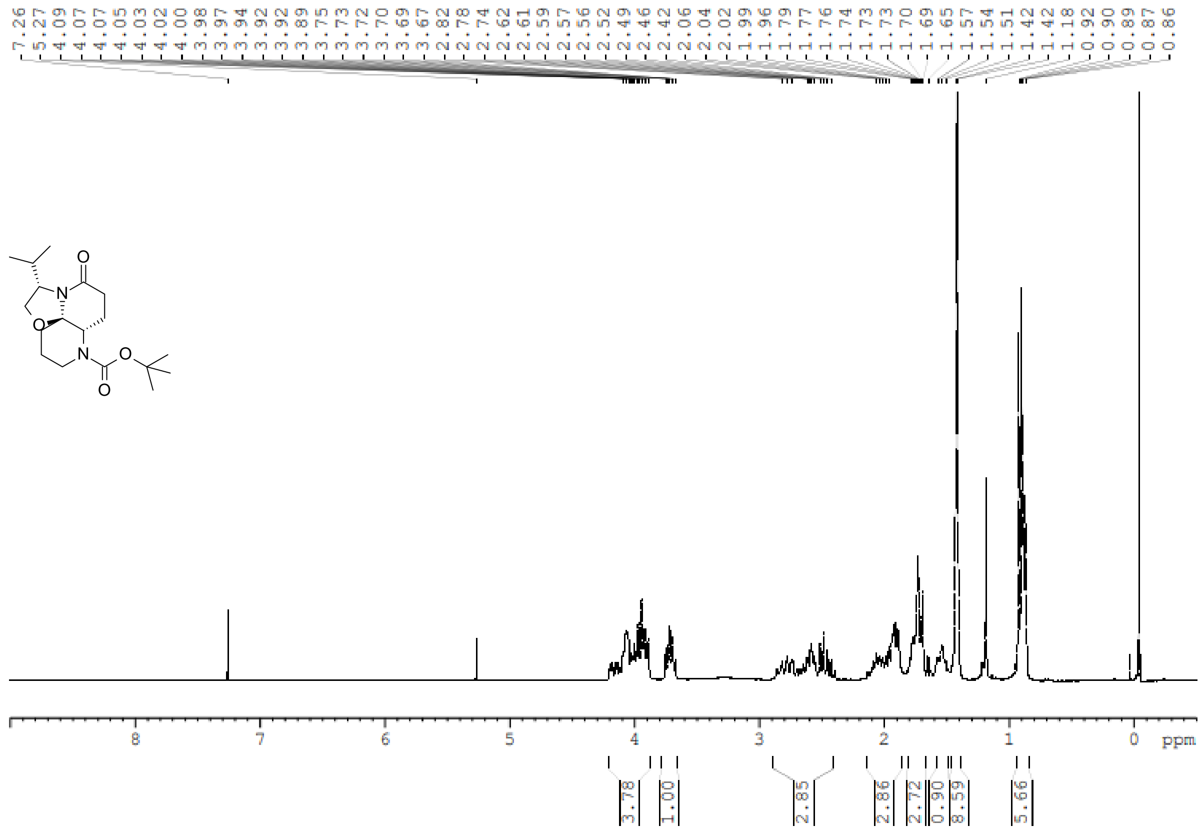

31, ^{13}C NMR (75 MHz, $CDCl_3$)

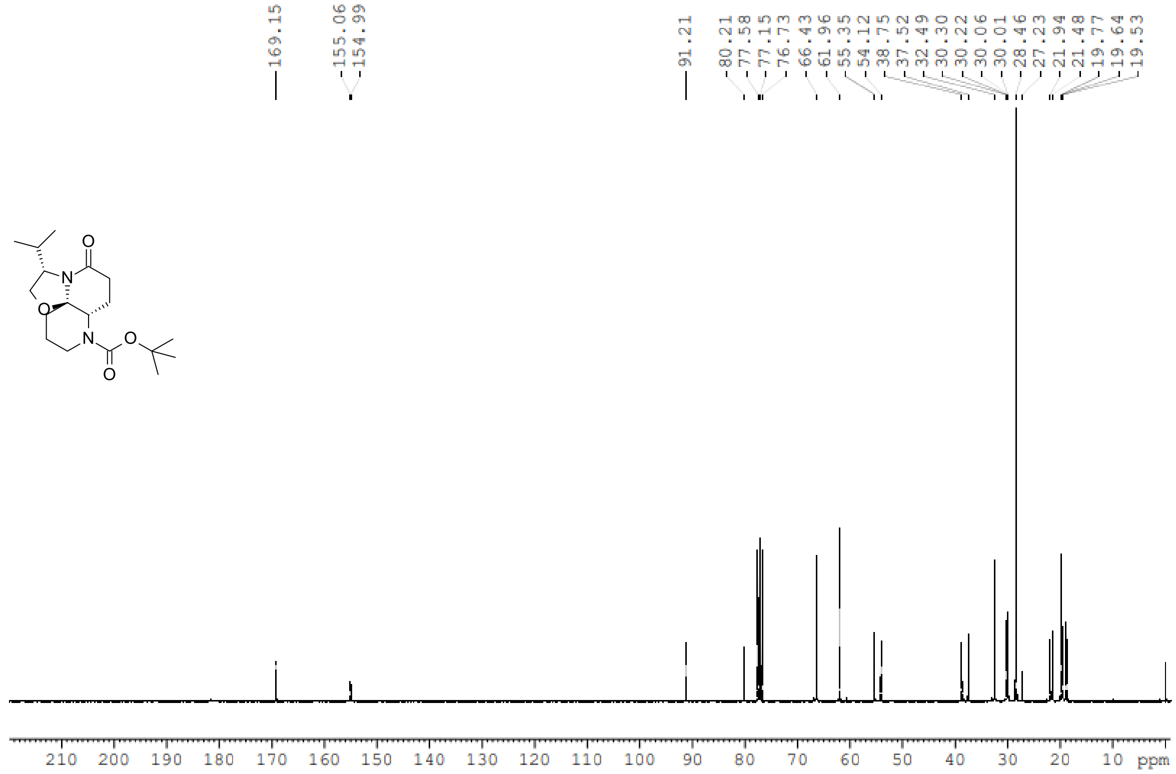

33, ^{1}H NMR (300 MHz, $CDCl_3$)

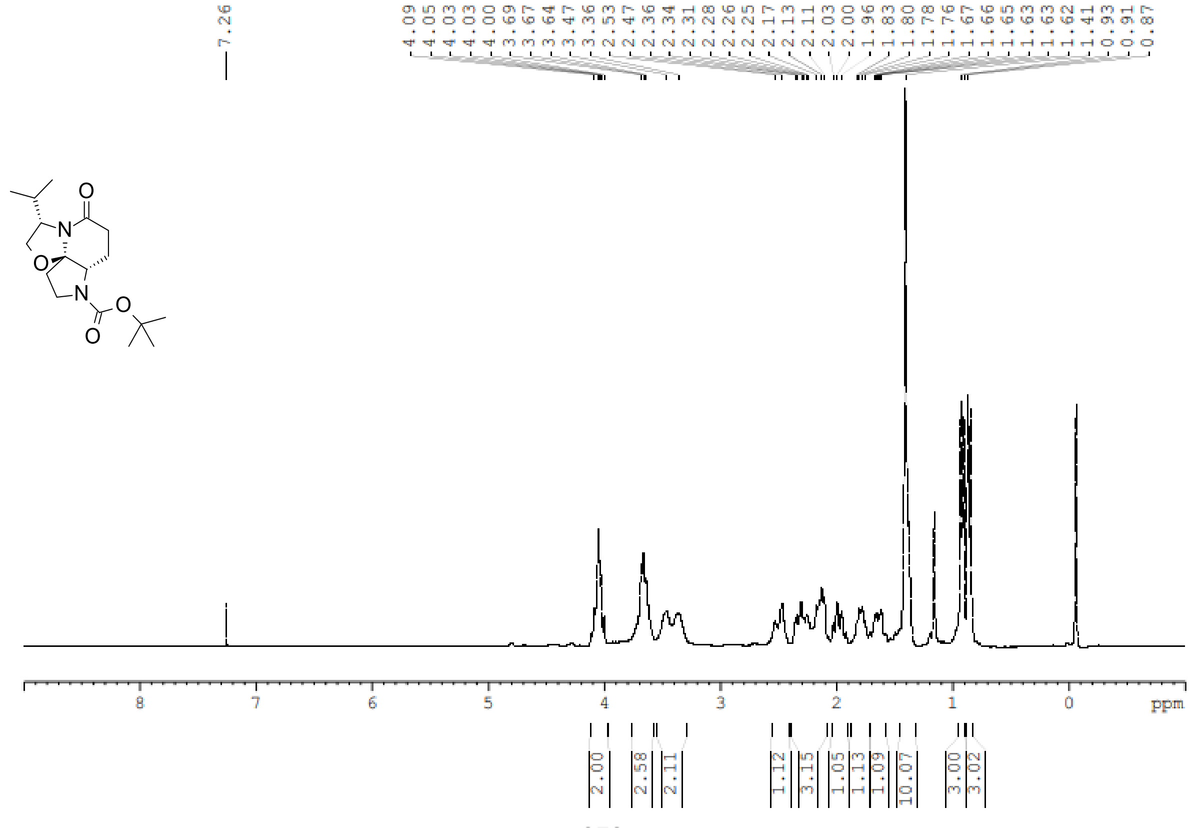

33, ^{13}C NMR (75 MHz, $CDCl_3$)

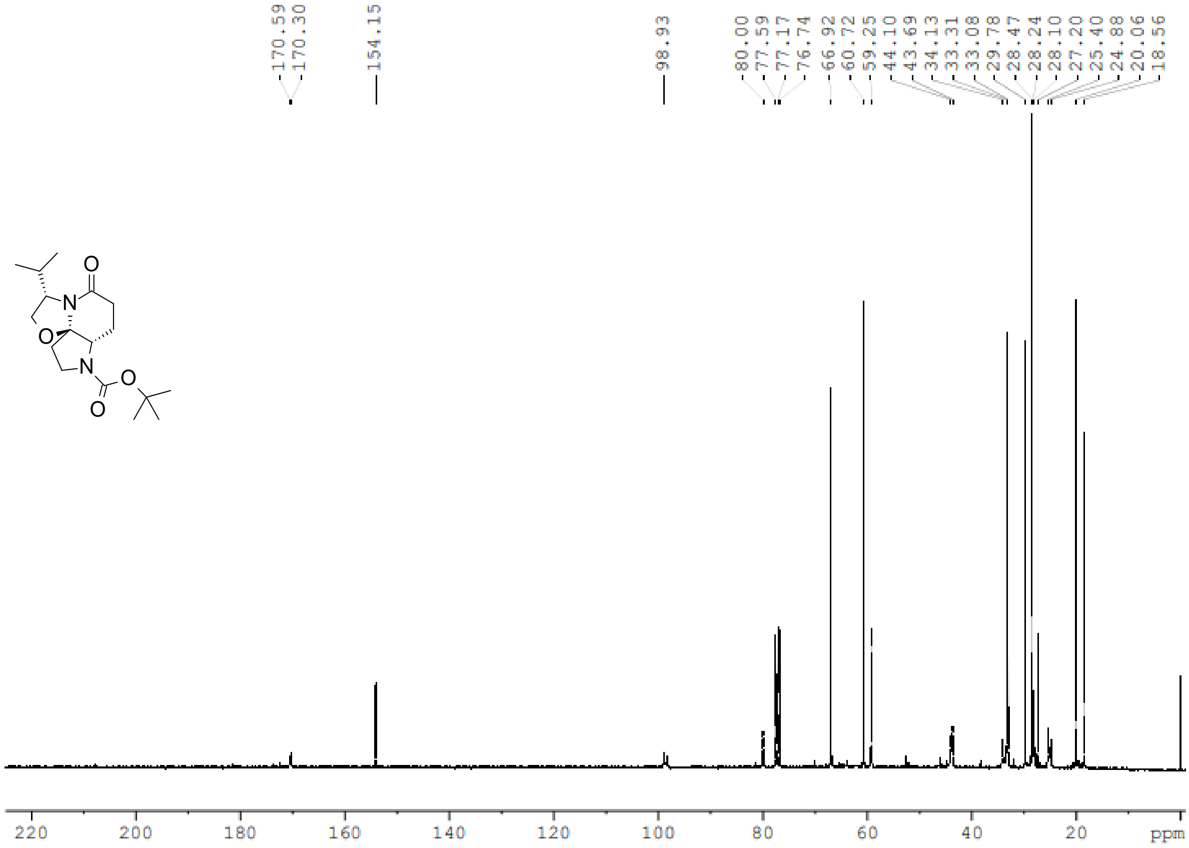

35, ^{1}H NMR (300 MHz, $CDCl_3$)

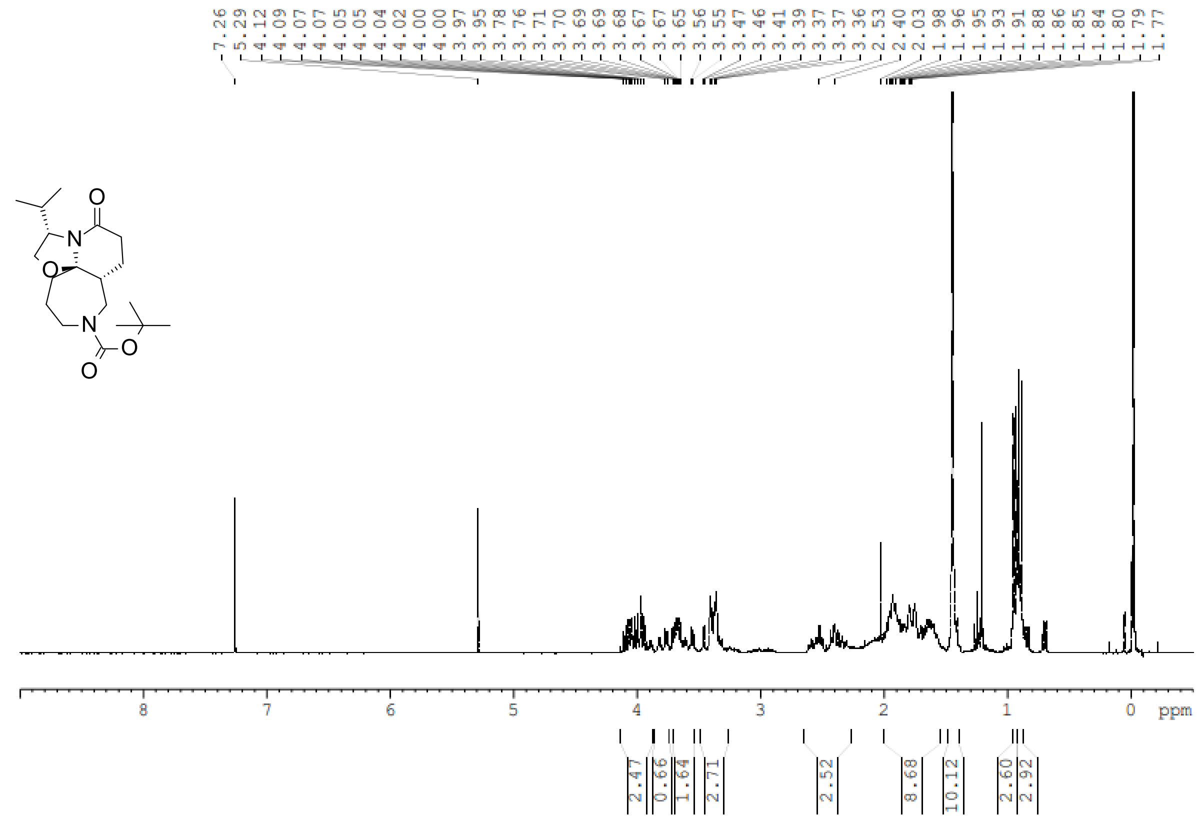

35, ^{13}C NMR (75 MHz, $CDCl_3$)

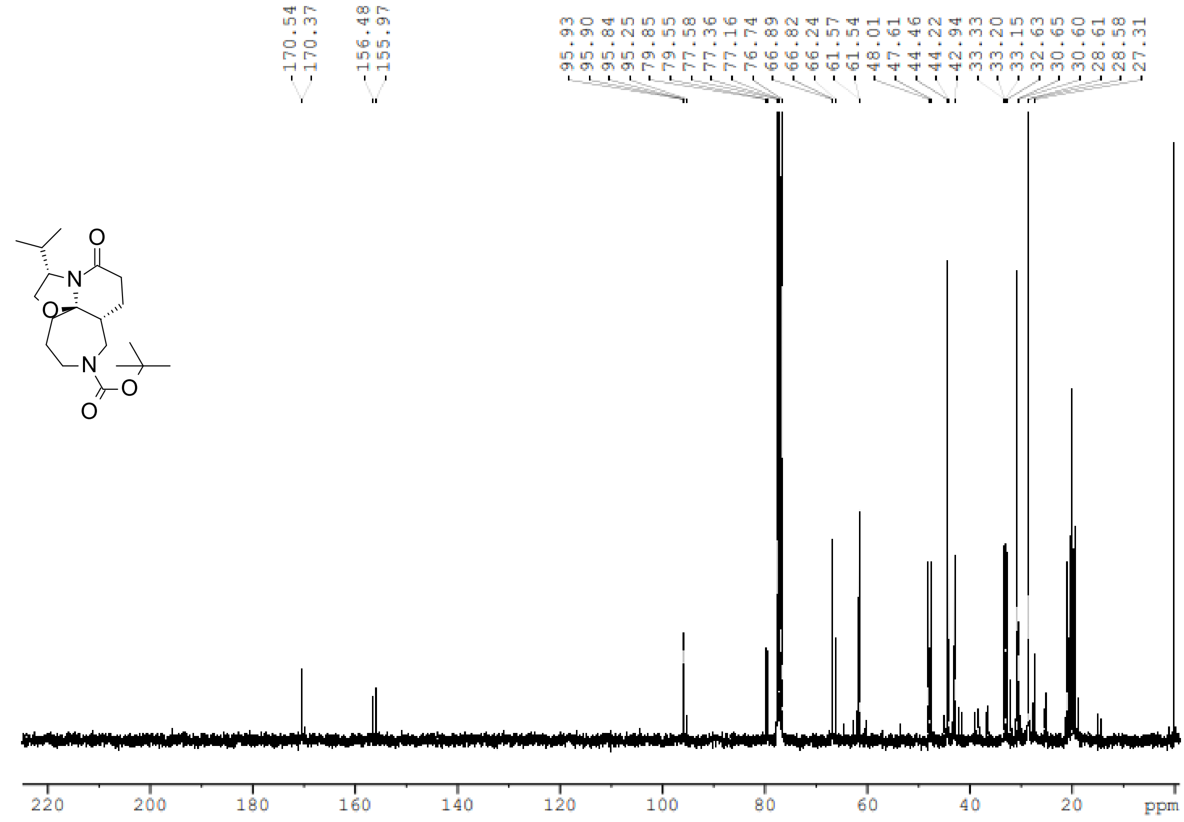

37, ^{1}H NMR (300 MHz, $CDCl_3$)

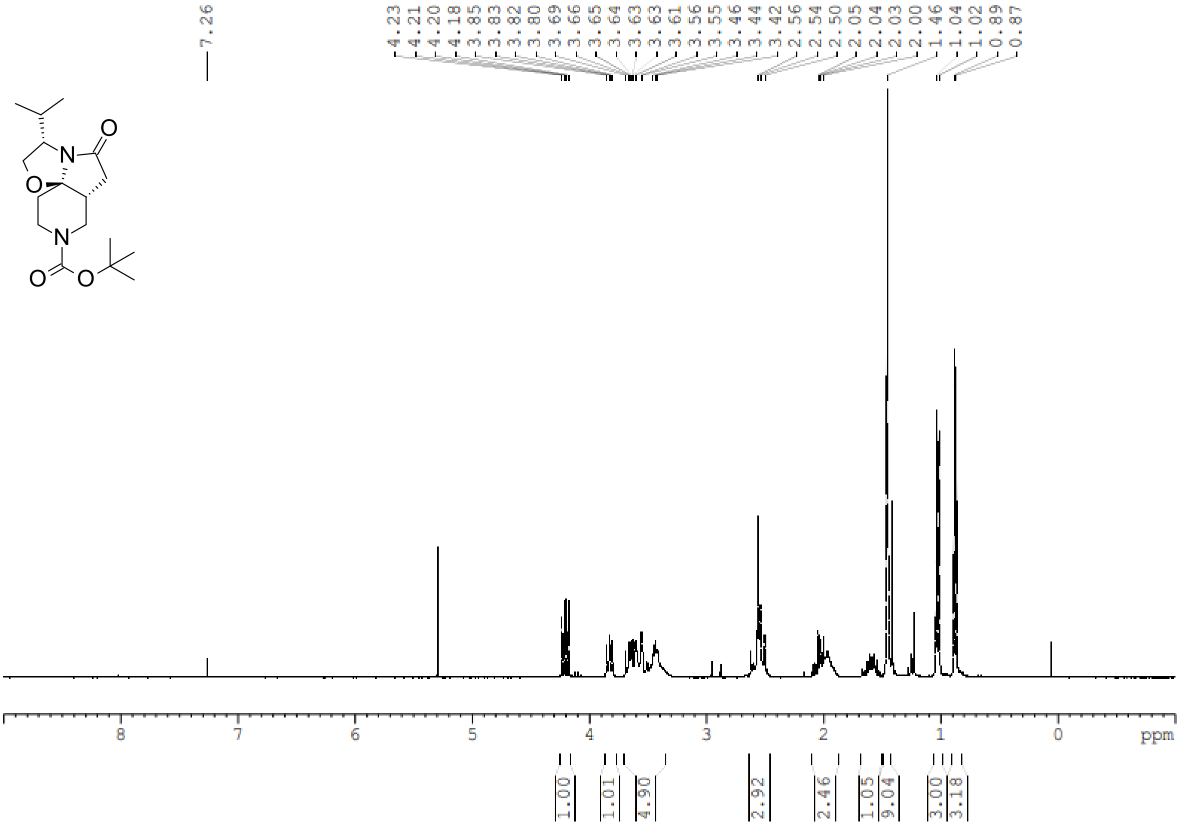

37, ^{13}C NMR (75 MHz, $CDCl_3$)

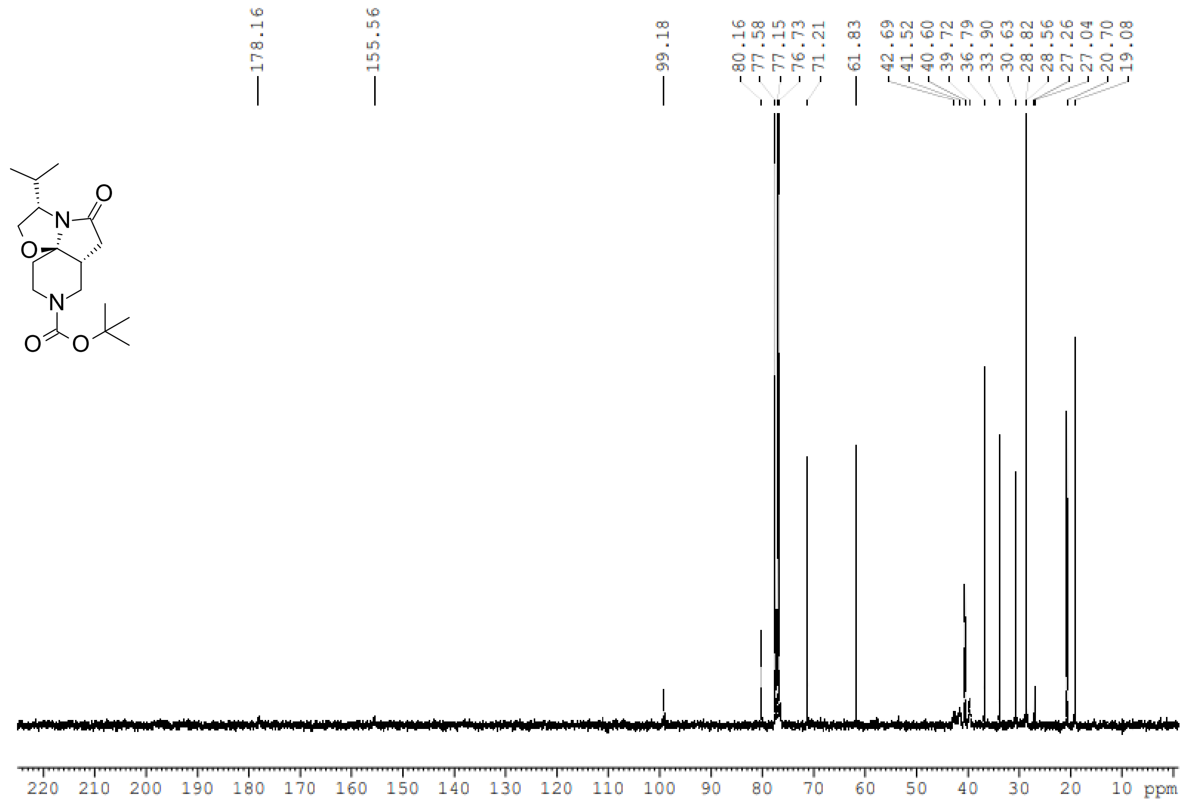

39, ^{1}H NMR (300 MHz, $CDCl_3$)

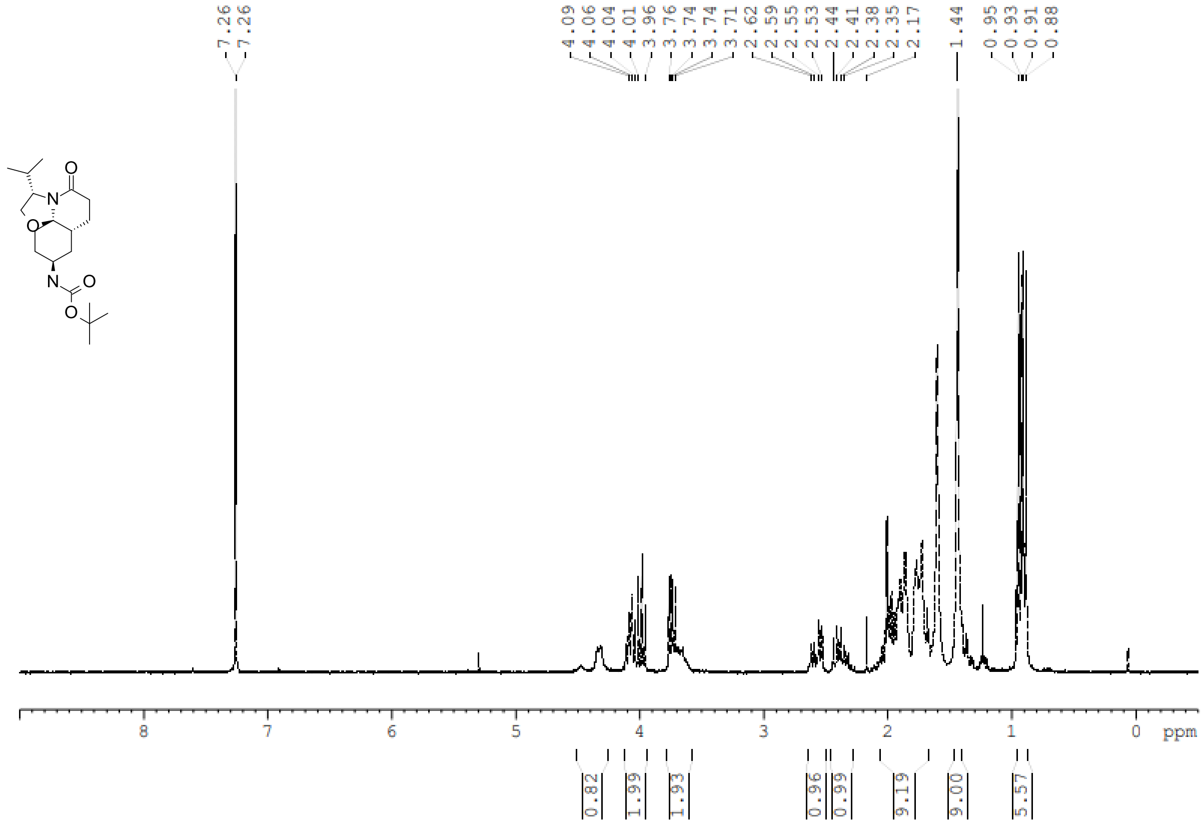

39, ^{13}C NMR (75 MHz, $CDCl_3$)

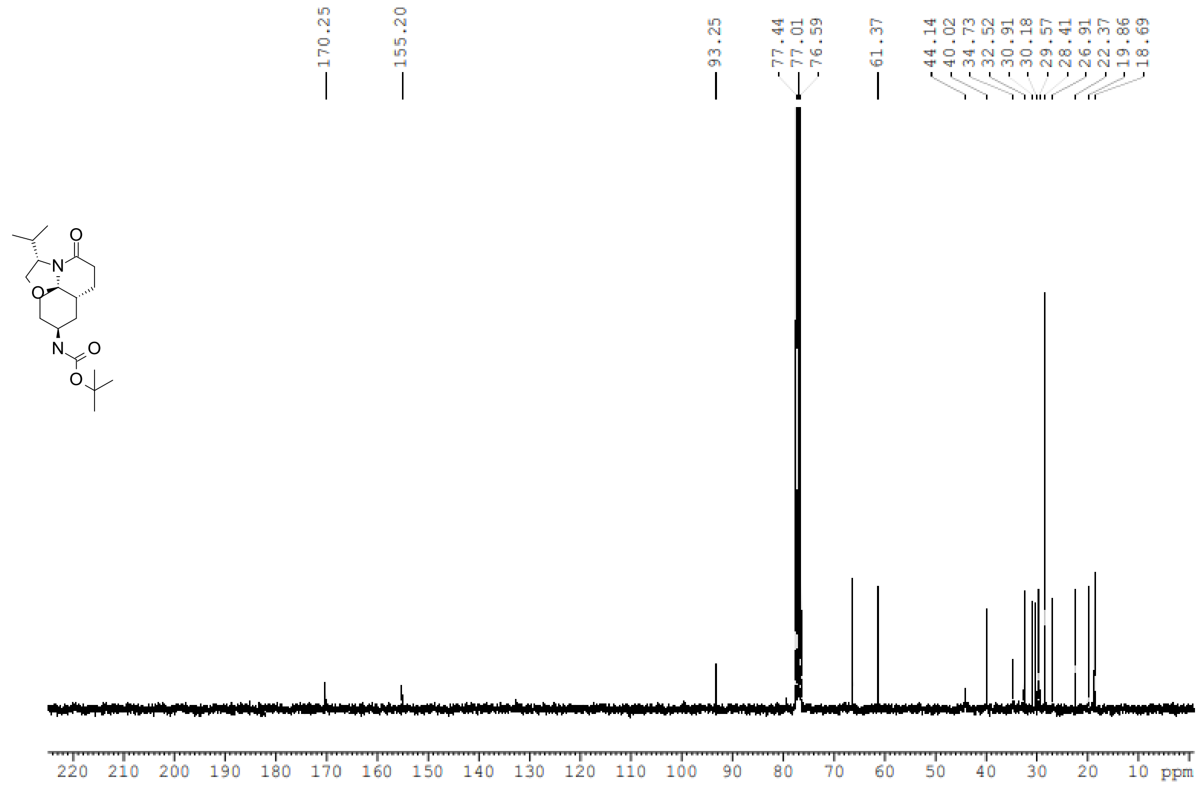

40, ^{1}H NMR (300 MHz, $CDCl_3$)

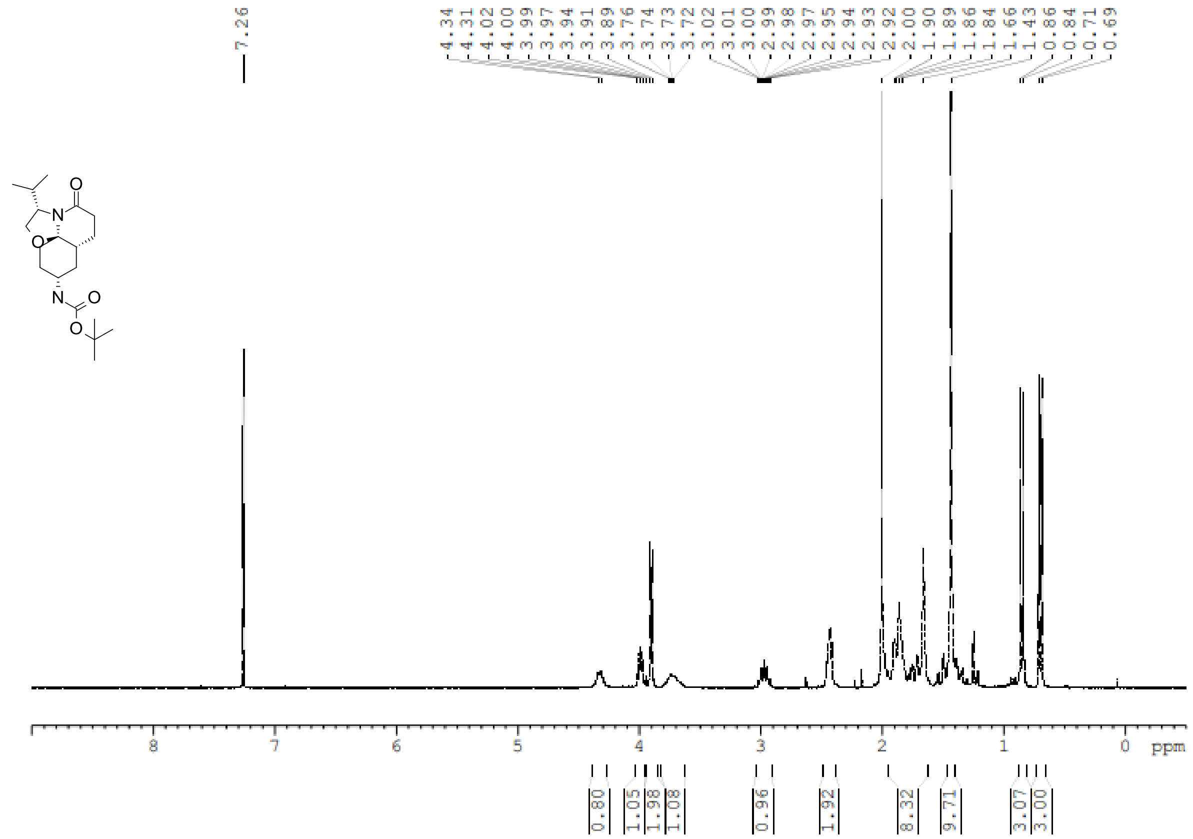

40, ^{13}C NMR (75 MHz, $CDCl_3$)

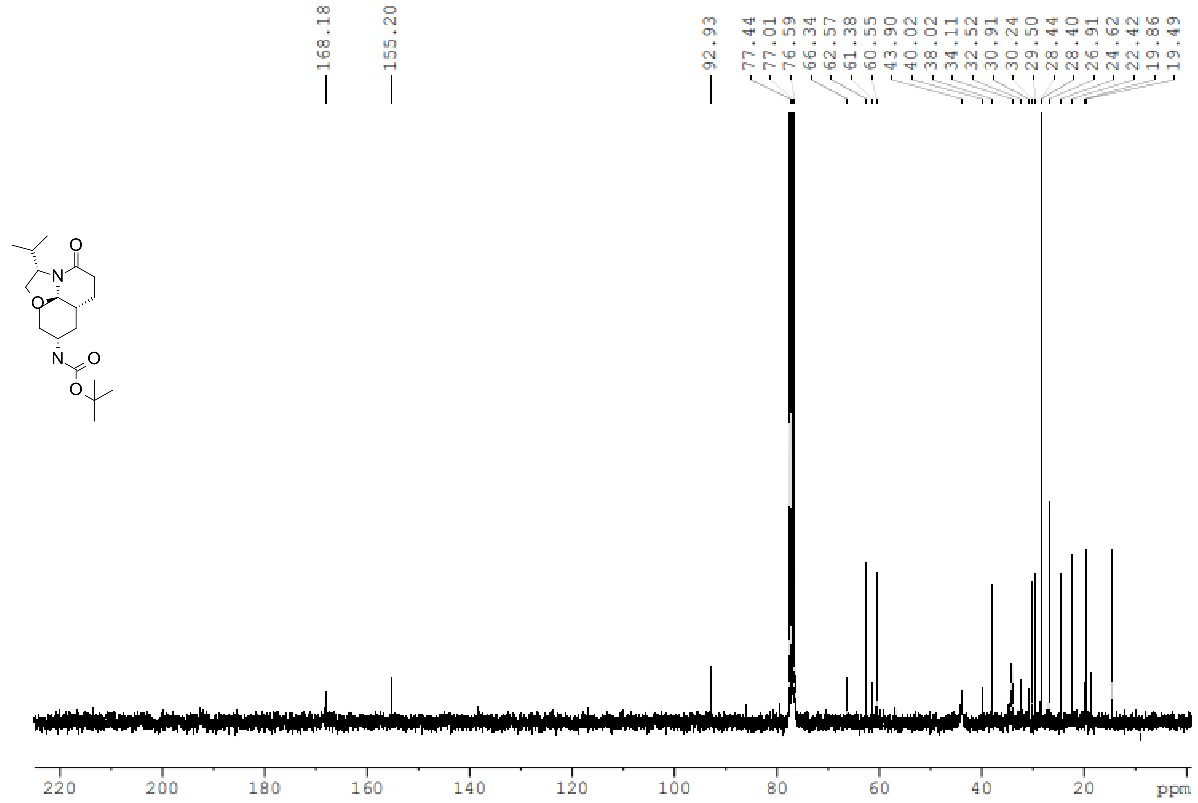

41 or 41' (diastereoisomer 1), ^{1}H NMR (300 MHz, $CDCl_3$)

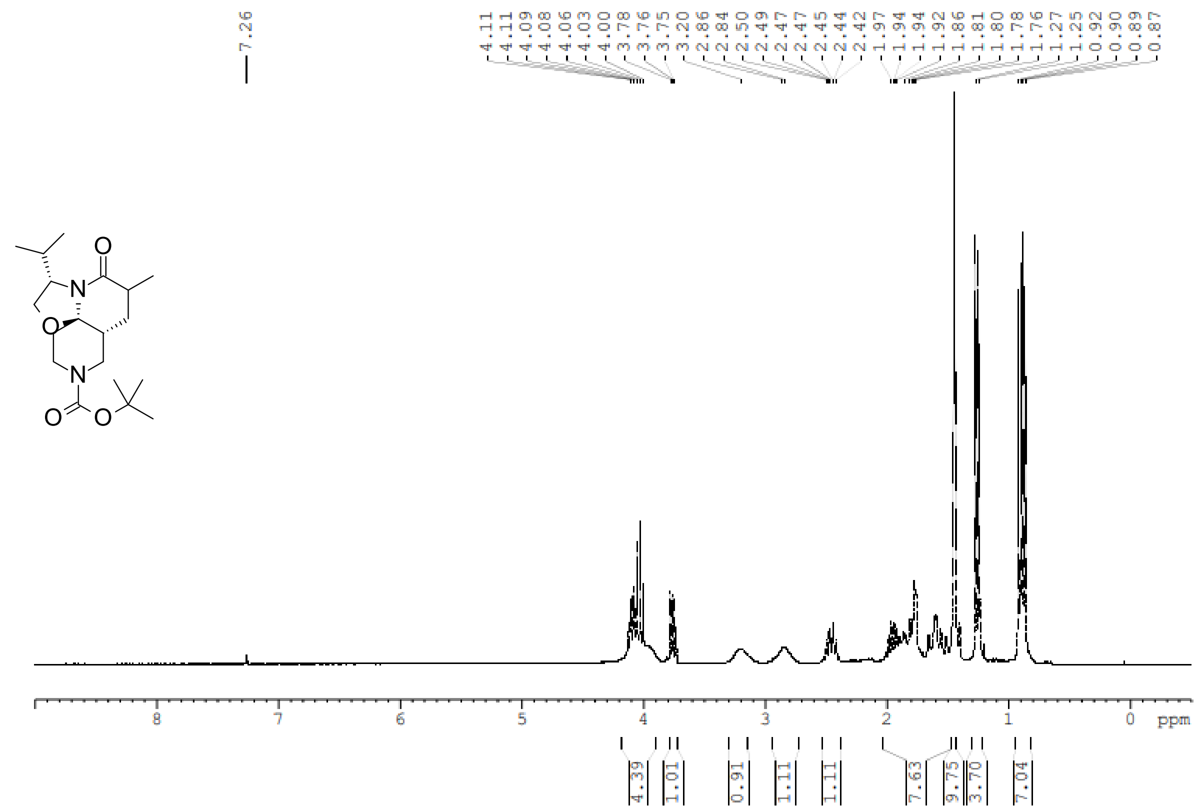

41 or 41' (diastereoisomer 1), ^{13}C NMR (75 MHz, $CDCl_3$)

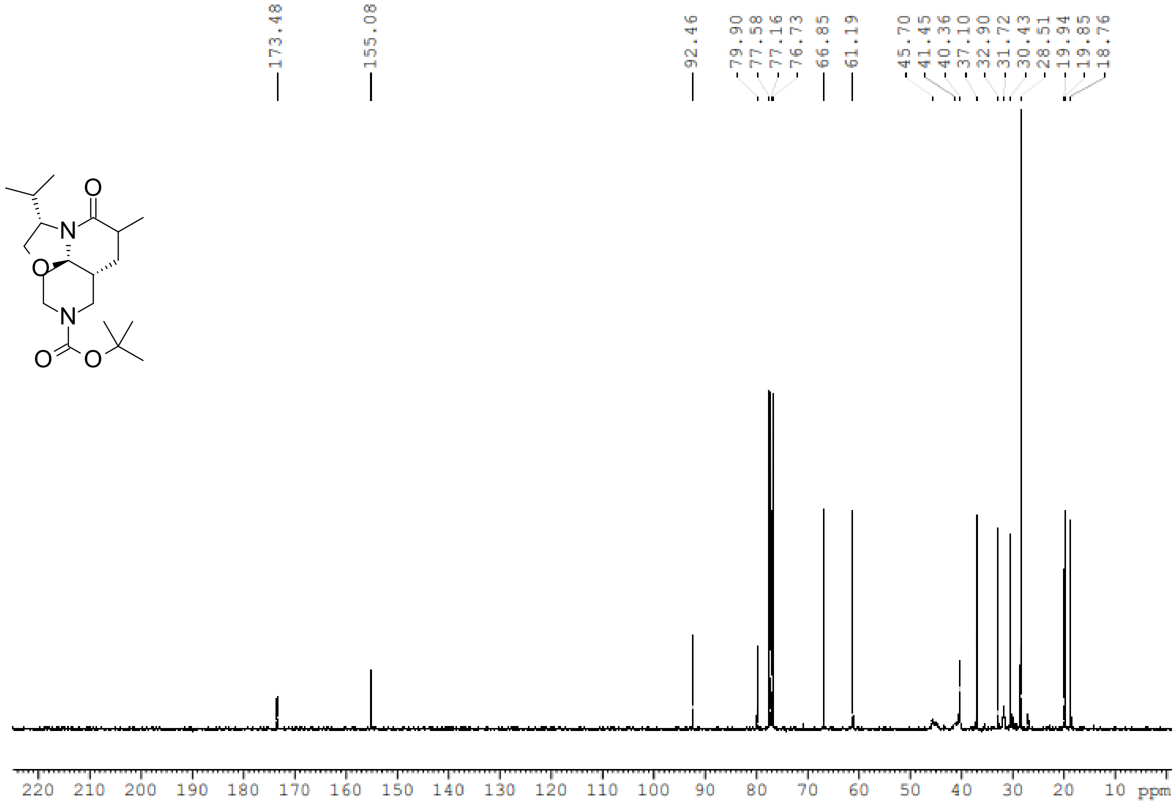

41 or 41' (diastereoisomer 2), ^{1}H NMR (300 MHz, $CDCl_3$)

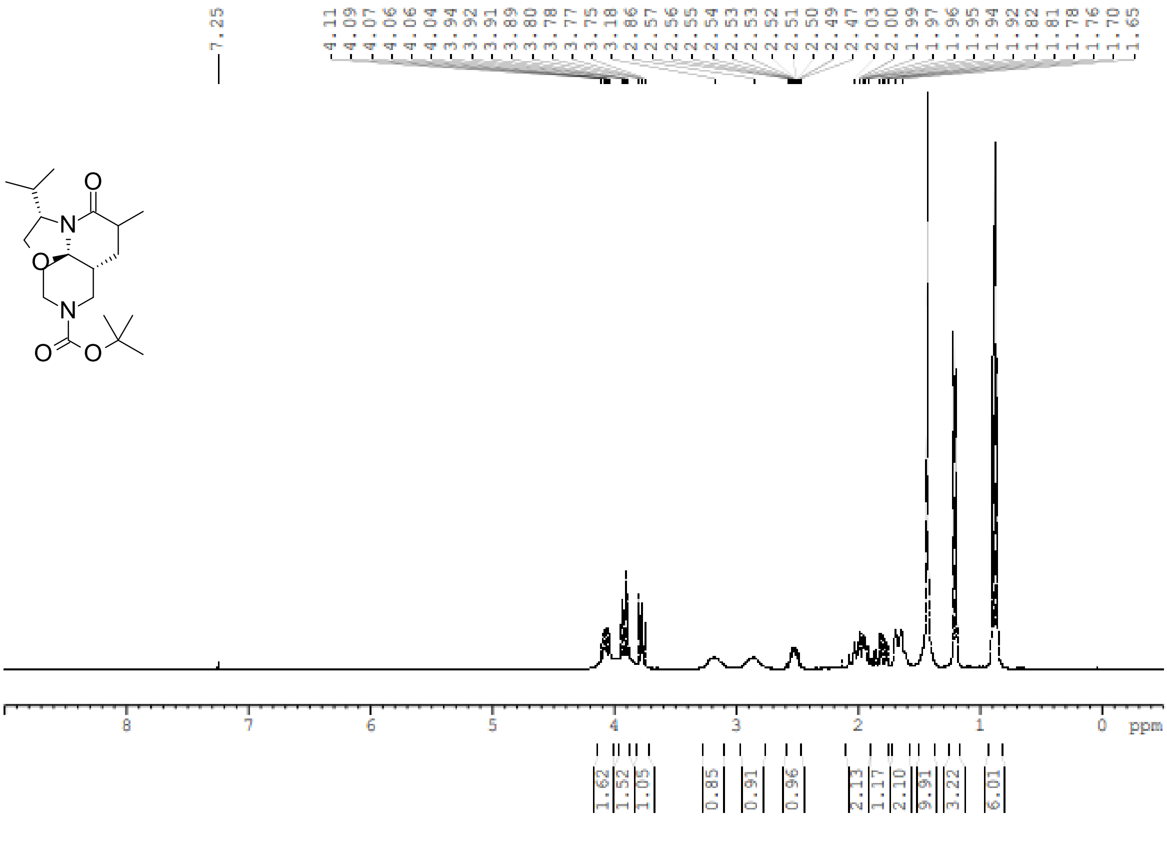

41 or 41' (diastereoisomer 2), ^{13}C NMR (75 MHz, $CDCl_3$)

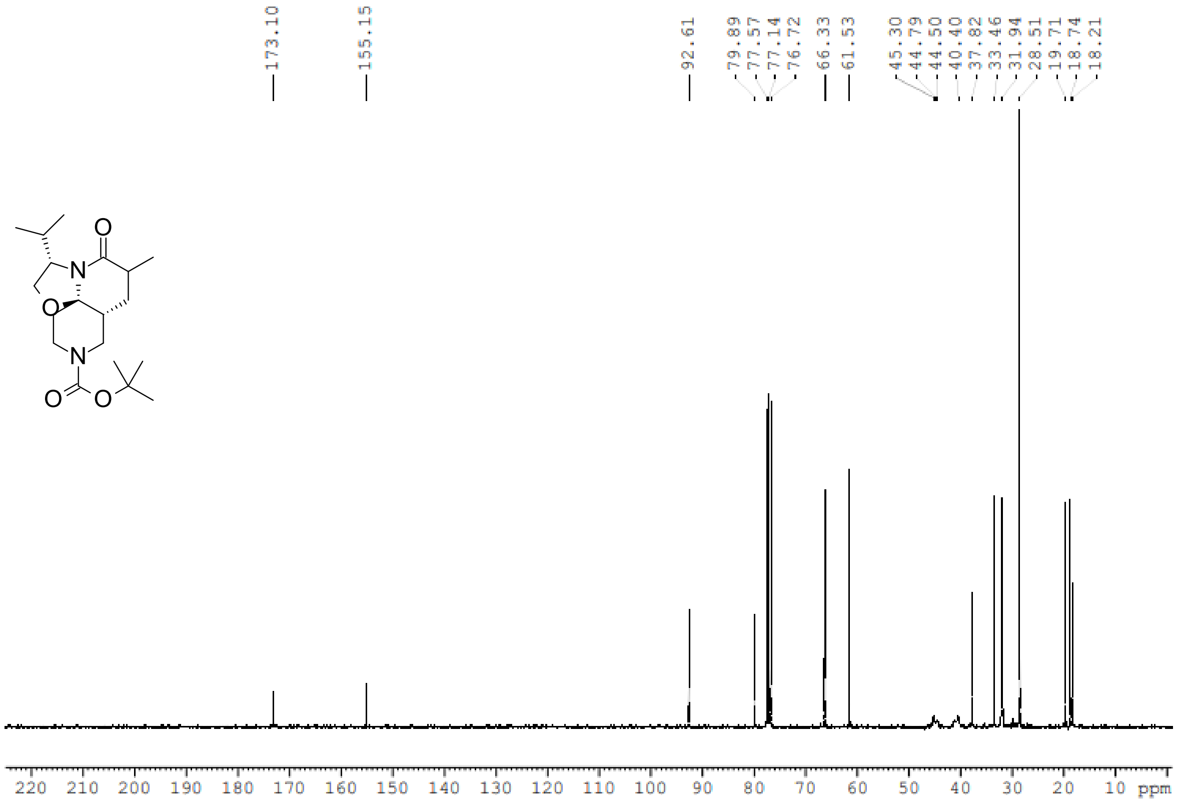

42, ^{1}H NMR (300 MHz, $CDCl_3$)

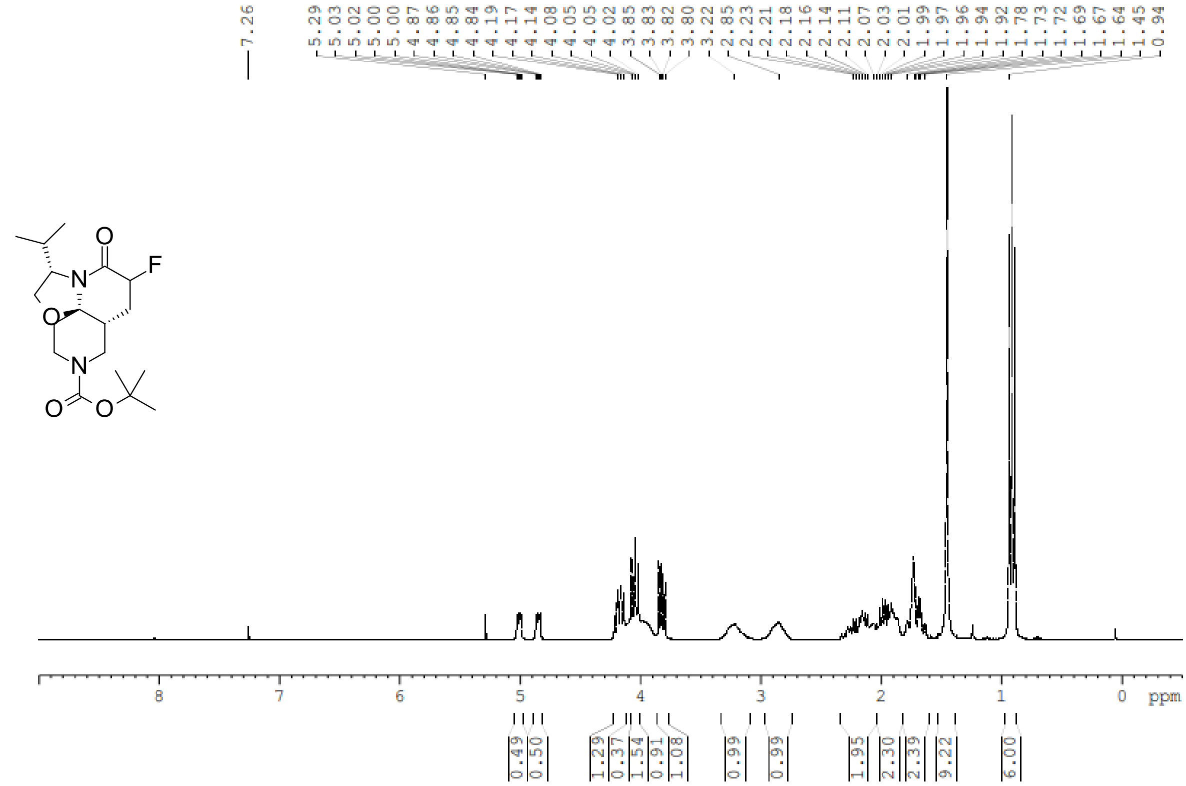

42, ^{13}C NMR (75 MHz, $CDCl_3$)

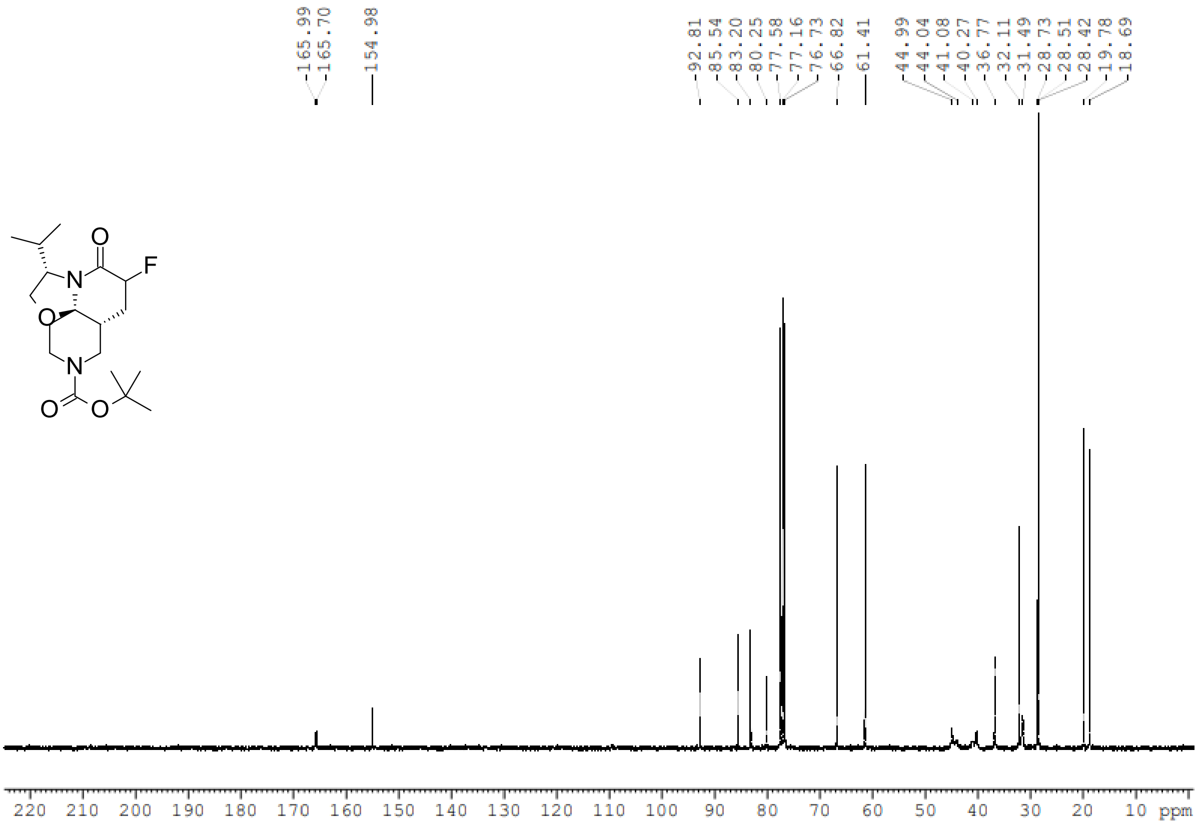

43, ^{1}H NMR (300 MHz, CD_3OD)

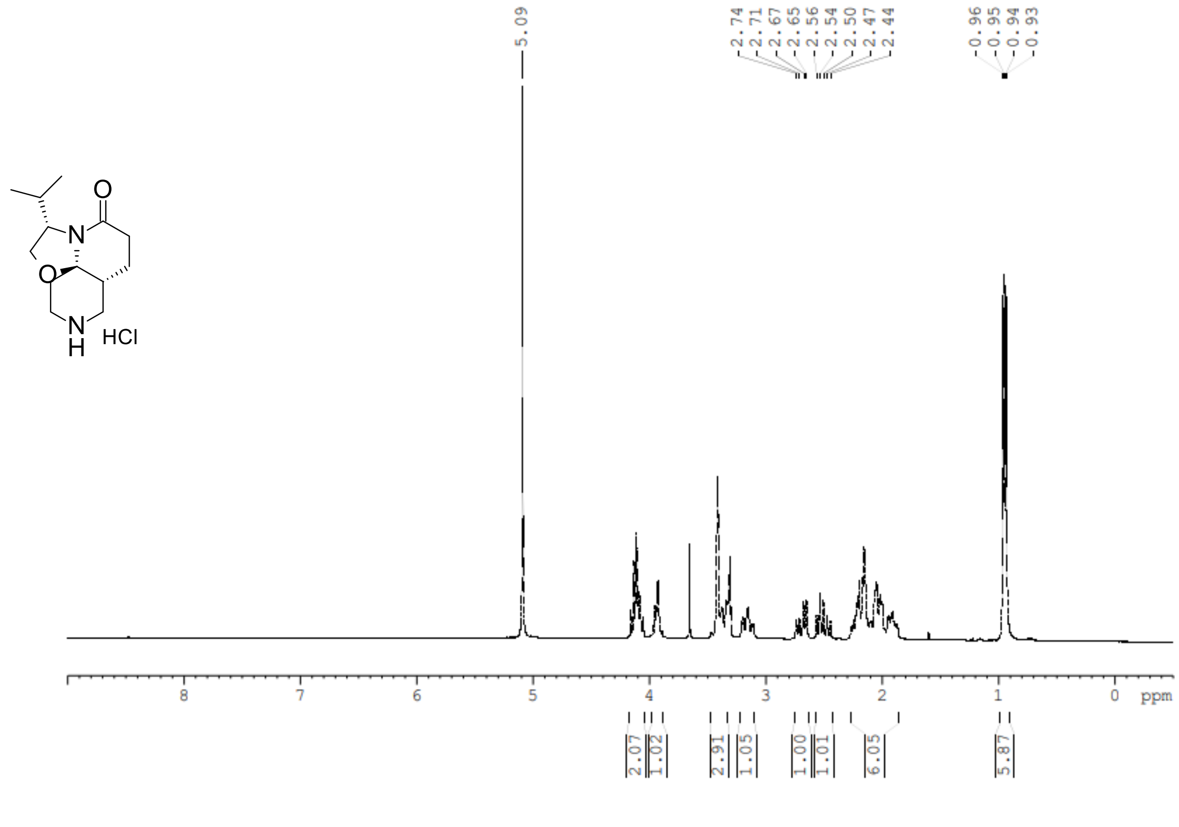

43, ^{13}C NMR (75 MHz, CD_3OD)

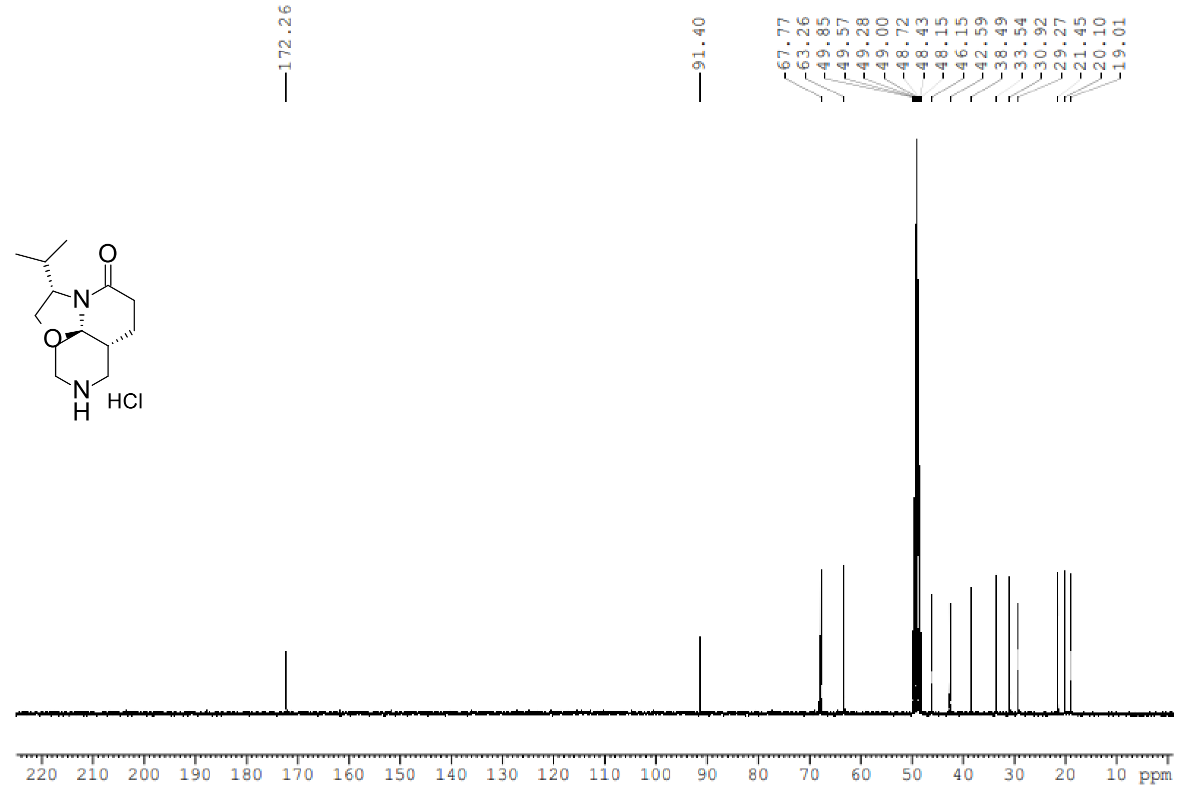

44, ^{1}H NMR (300 MHz, CD_2Cl_2)

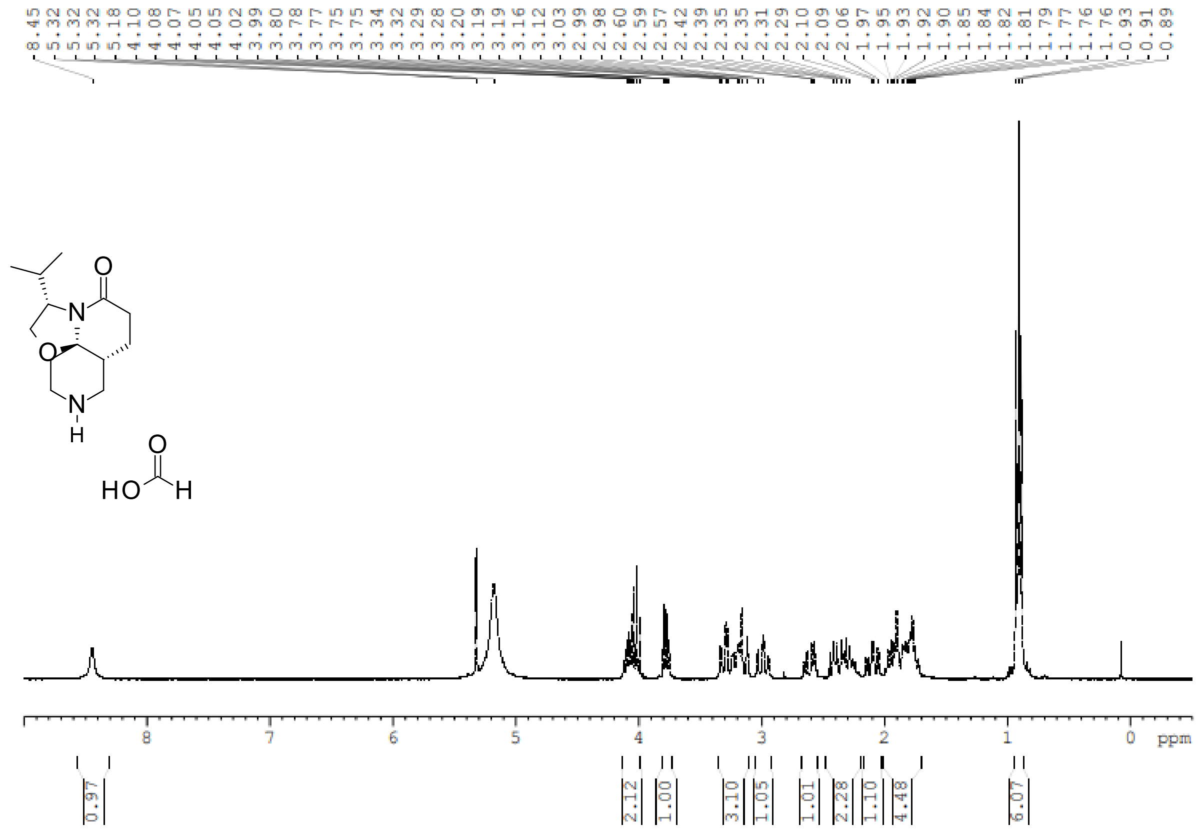

45, ^{1}H NMR (300 MHz, CD_2Cl_2)

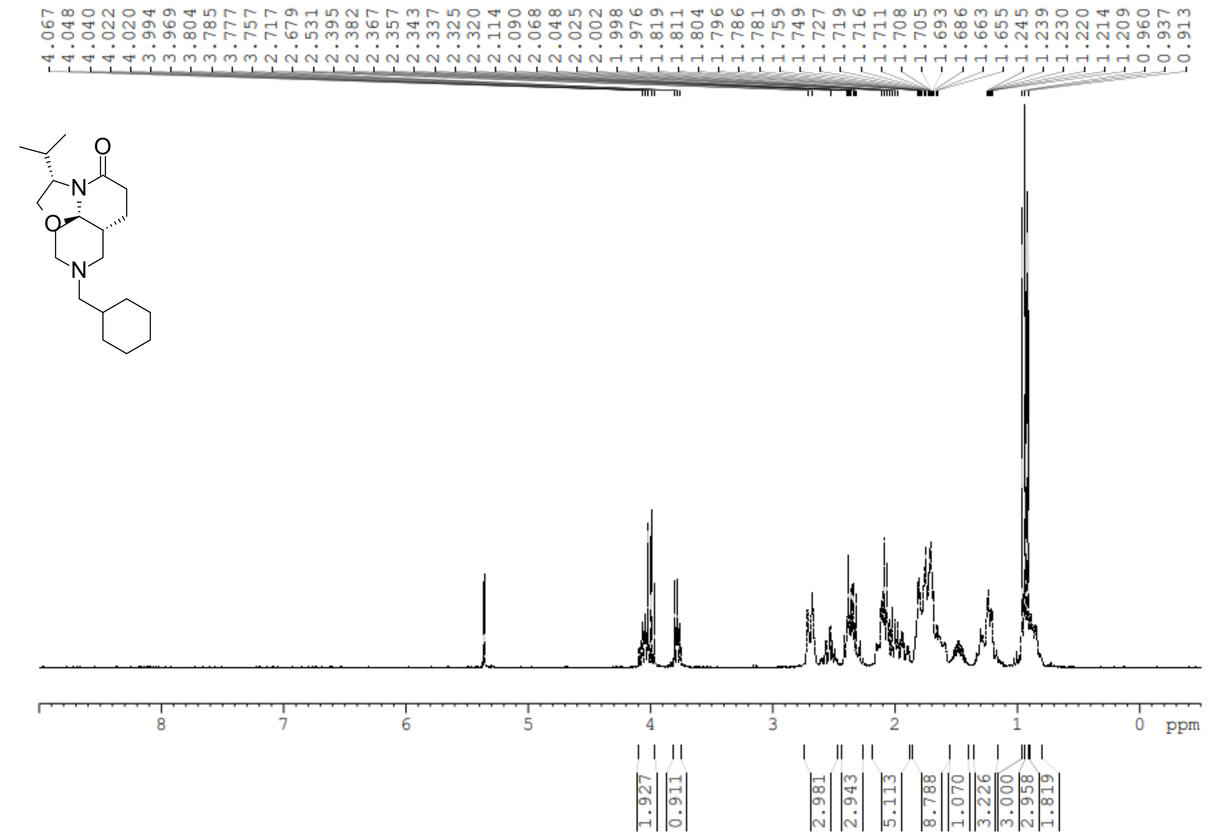

45, ^{13}C NMR (75 MHz, CD_2Cl_2)

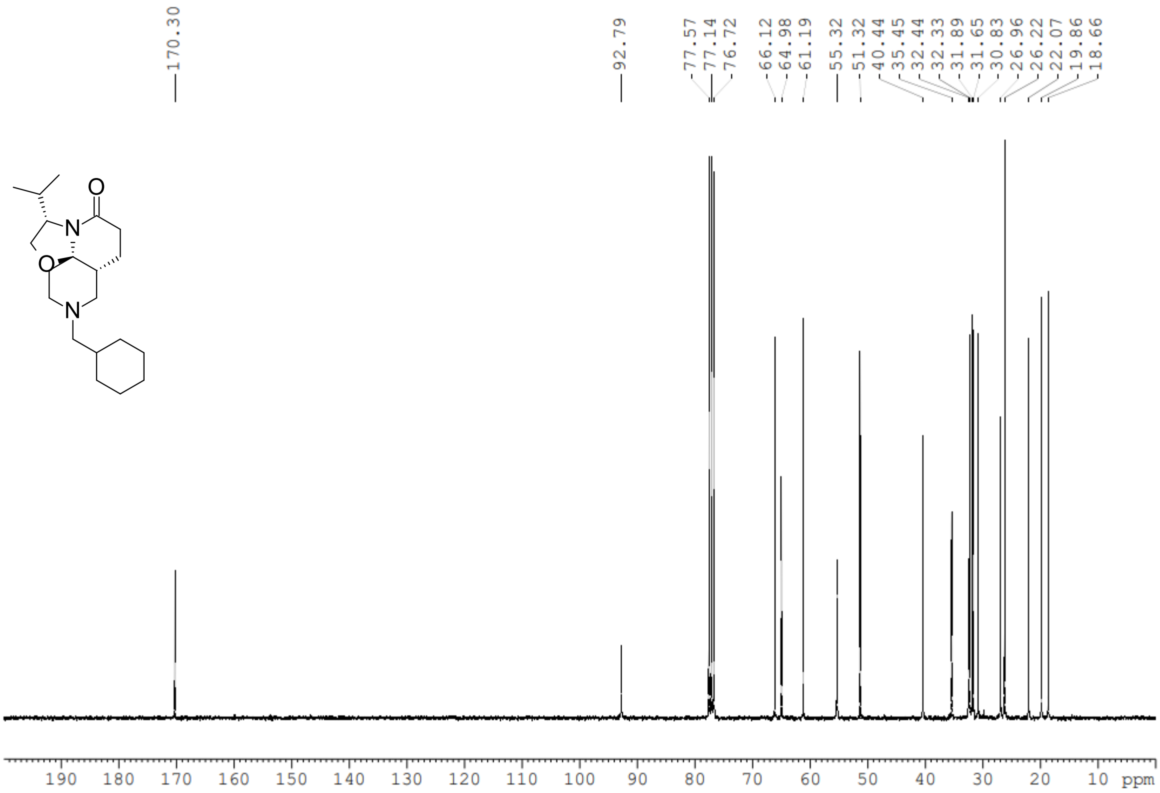

46, ^{1}H NMR (300 MHz, $CDCl_3$)

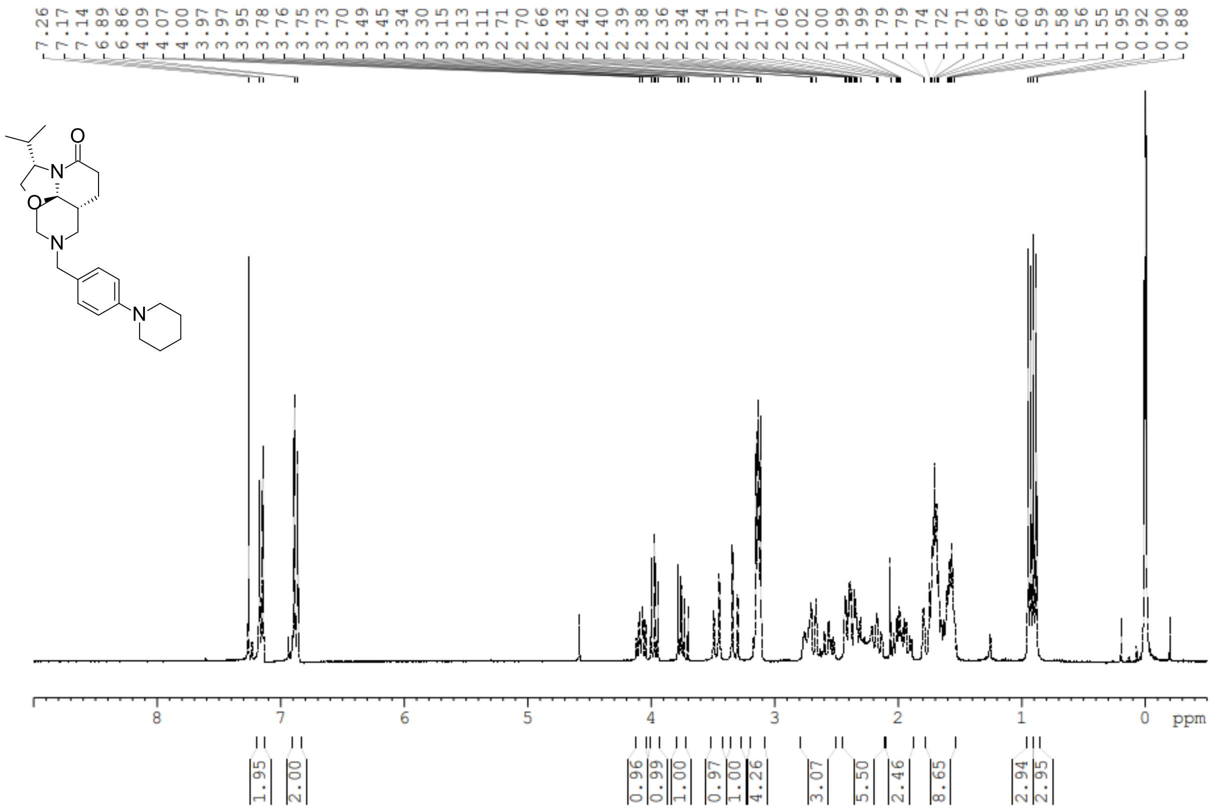

46, ^{13}C NMR (75 MHz, $CDCl_3$)

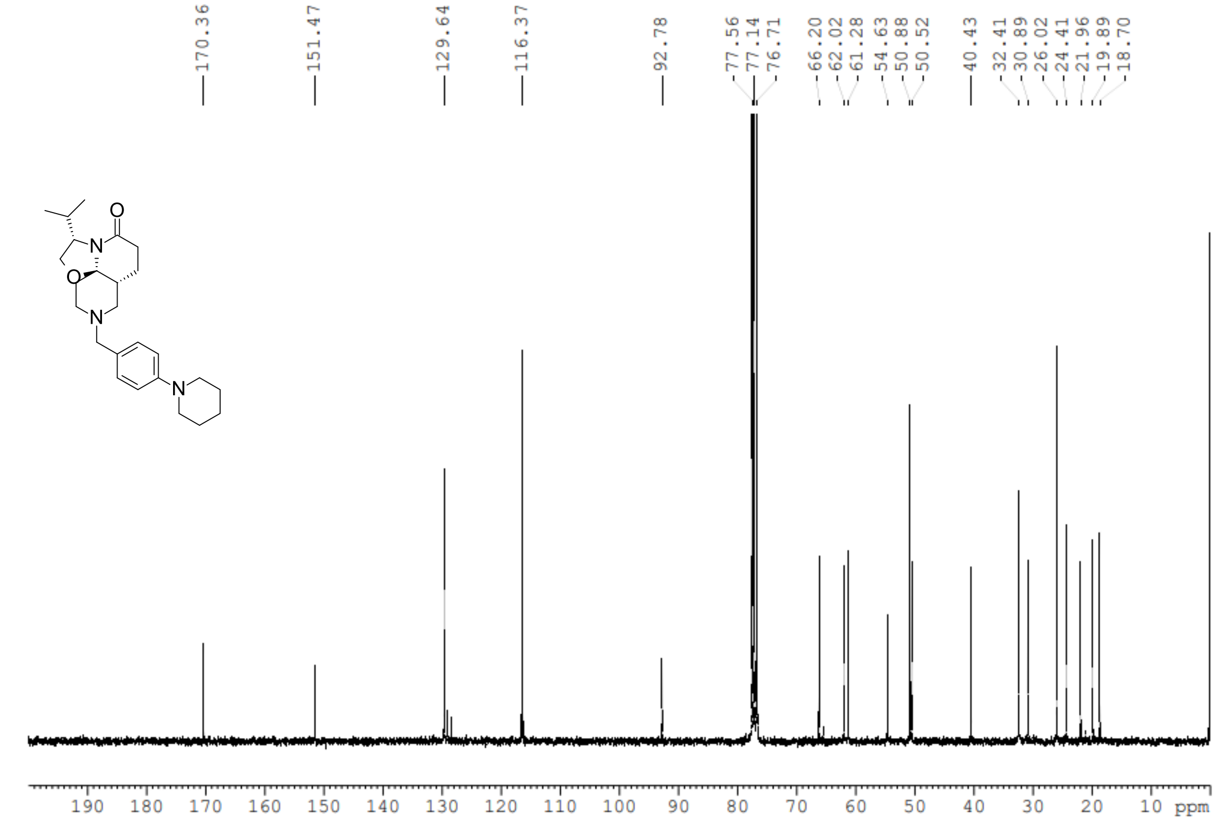

47, ^{1}H NMR (300 MHz, $CDCl_3$)

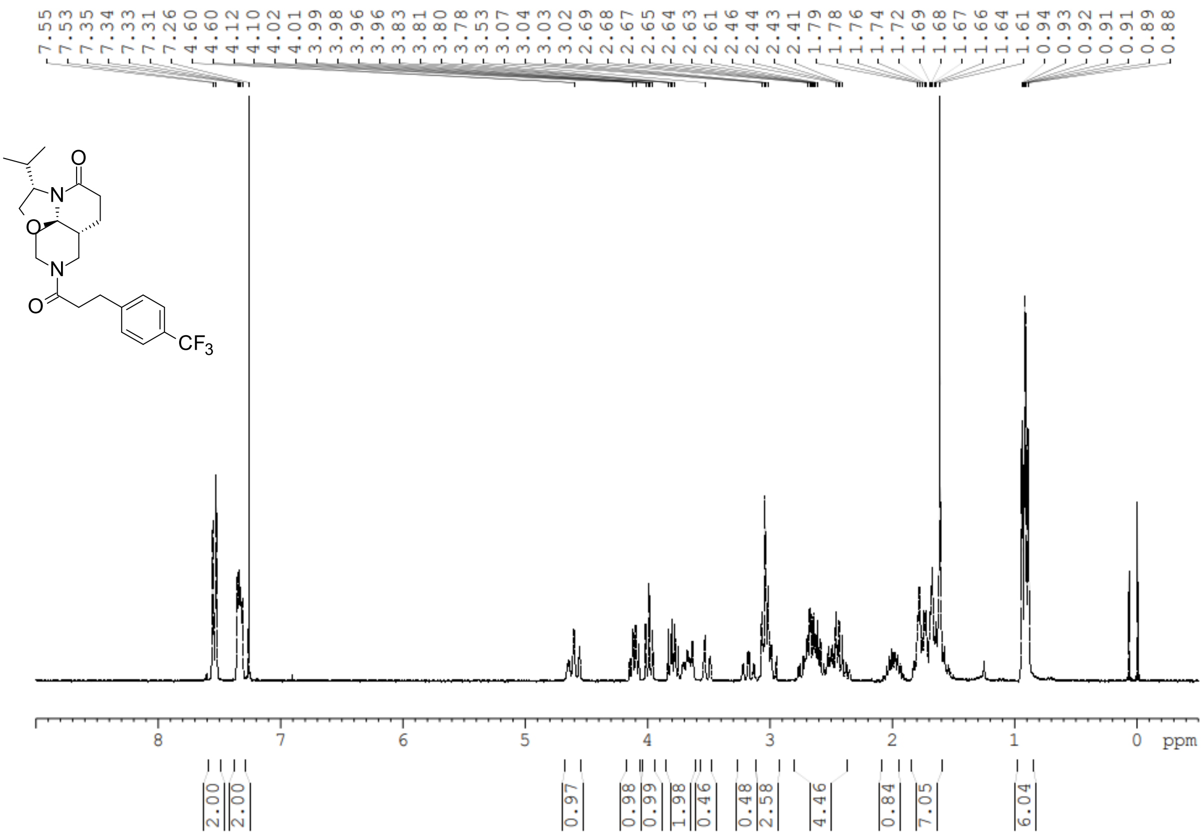

47, ^{13}C NMR (75 MHz, $CDCl_3$)

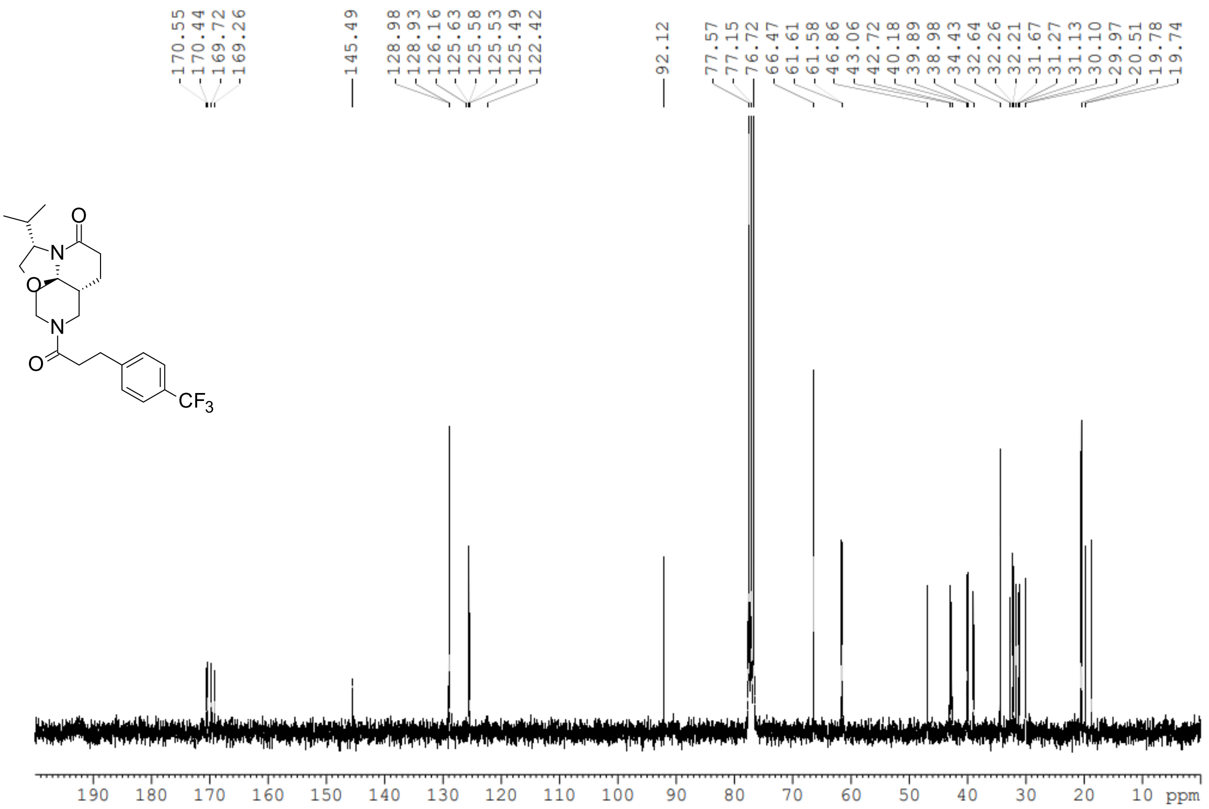

48, ^{1}H NMR (300 MHz, $CDCl_3$)

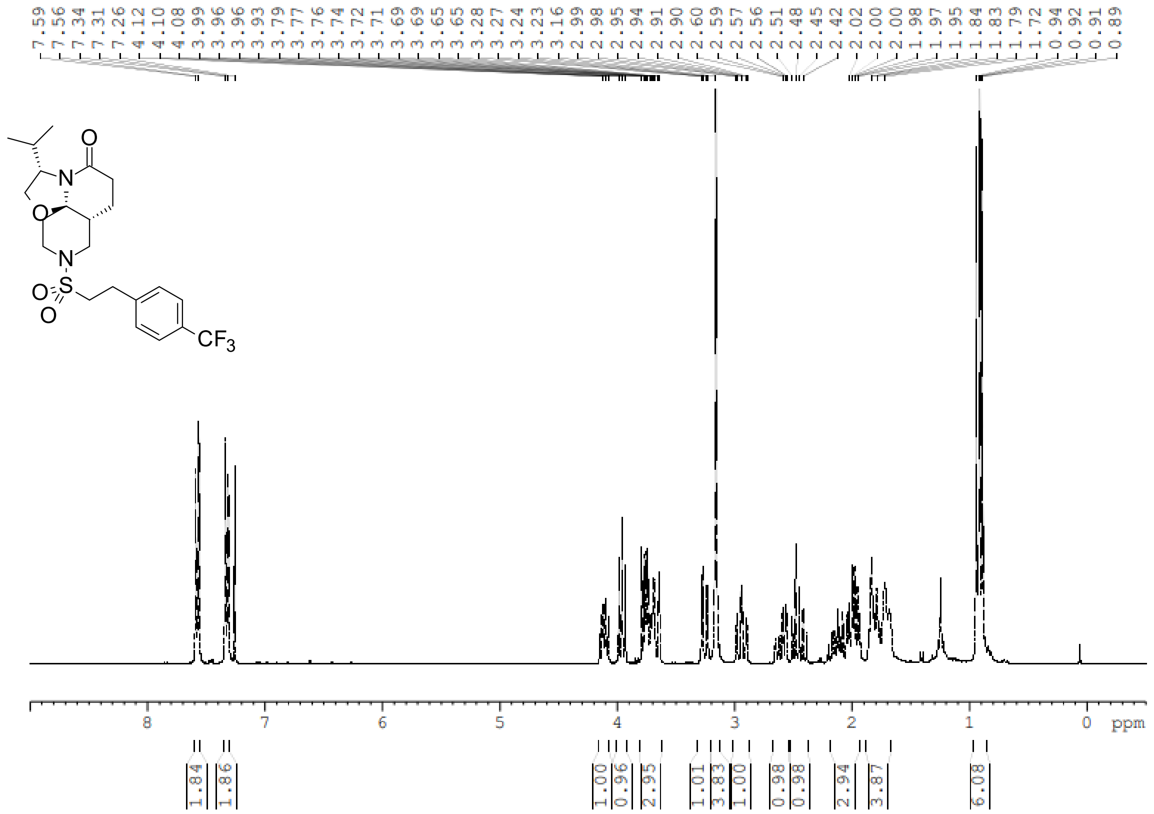

48, ^{13}C NMR (75 MHz, $CDCl_3$)

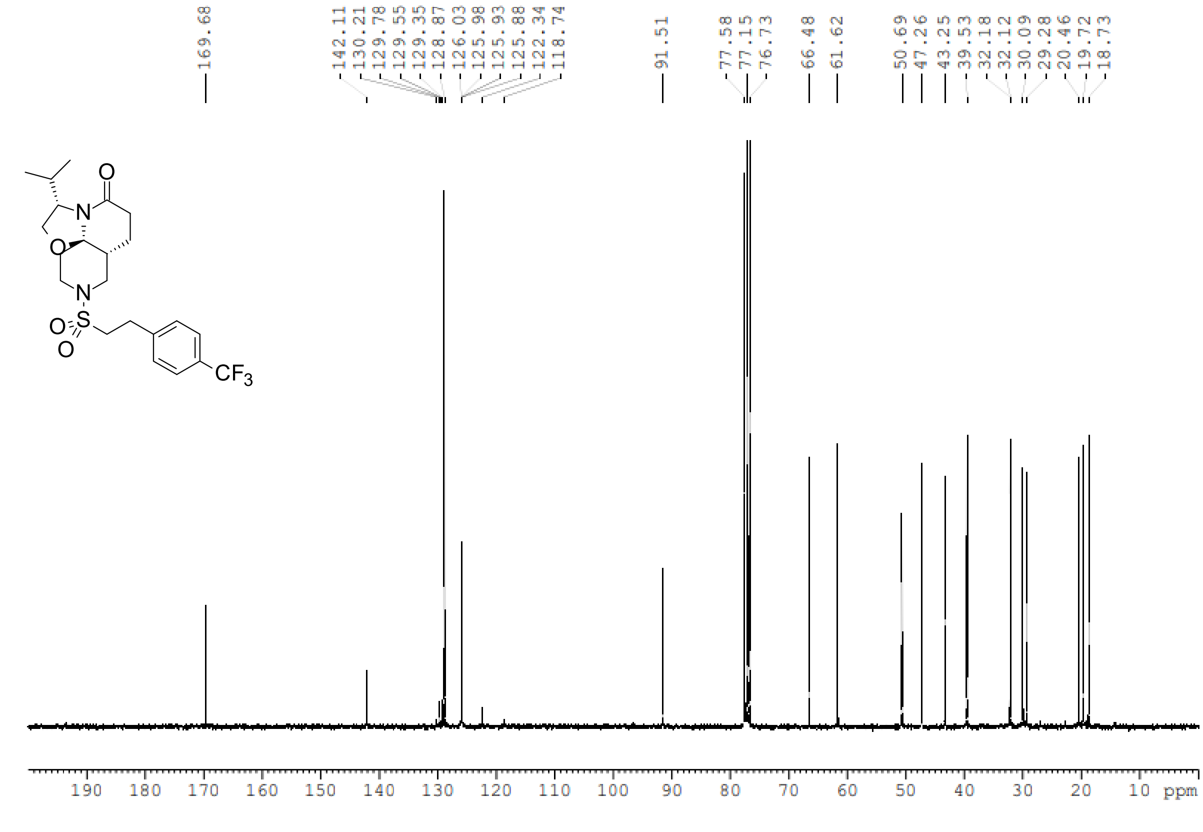

Supplement: Supplementary file 1 [file pharmaceuticals-16-00413-s001.zip › pharmaceuticals-2264540-supplementary.pdf]
